# Mitochondrial-generated G-quadruplex-forming lncRNAs regulate heme homeostasis

**DOI:** 10.1016/j.isci.2026.116425

**Published:** 2026-06-17

**Authors:** Vinodh J. Sahayasheela, Ryohei Noizumi, Manendra B. Lankadasari, Takumi Terada, Mitsuharu Ooga, Atharv Kulkarni, Shinjiro Suzuki, Takuya Hidaka, Li Cai, Ganesh N. Pandian, Toshikazu Bando, Amit R. Reddi, Hiroshi Sugiyama

**Affiliations:** 1 Department of Chemistry, Graduate School of Science, Kyoto University, Kitashirakawa-Oiwakecho, Sakyo-Ku, Kyoto 606-8502, Japan; 2 Institute for Integrated Cell-Material Sciences (WPI-iCeMS), Kyoto University, Yoshida-Uninomiyacho, Sakyo-Ku, Kyoto 606-8501, Japan; 3 Human Biology Microbiome Quantum Research Center (WPI-Bio2Q), Keio University, Tokyo 160-8582, Japan; 4 Thoracic Service, Department of Surgery, Memorial Sloan Kettering Cancer Center, New York, NY 10065, USA; 5 Department of Biomedical Engineering, Rutgers University, Piscataway, NJ 08854, USA; 6 School of Chemistry and Biochemistry, Georgia Institute of Technology, Atlanta, GA, USA; 7 Parker Petit Institute for Bioengineering and Biosciences, Atlanta, GA, USA

**Keywords:** biochemistry, molecular biology, biophysics

## Abstract

Heme is an essential but potentially toxic prosthetic group synthesized in mitochondria. Maintaining mitochondrial heme homeostasis requires precise regulation of labile heme availability. Here, we show that mitochondria-generated long non-coding RNAs (mt-lncRNAs) are enriched in G-quadruplex-forming sequences and that these RNA G-quadruplex (rG4) structures bind and buffer heme. Using G4-specific pull-down and bio-orthogonal imaging, we demonstrate rG4 formation in mt-lncRNAs inside cells and show that mt-lncRNA rG4s bind hemin *in vitro*. Using orthogonal chemical perturbations—a mitochondria-targeted pyrrole-imidazole polyamide (MITO-PIP), which depletes mt-lncRNAs by inhibiting L-strand transcription, and MITO-pyridostatin derivative (MITO-PyPDS), which competitively displaces heme from rG4 structures—combined with genetically encoded heme sensors and ρ0 cells, disrupting mt-lncRNA rG4s increased labile mitochondrial heme, elevated nuclear and cytoplasmic heme, induced reactive oxygen species, and upregulated heme oxygenase 1 (HMOX-1). These findings establish an RNA structure-based mechanism for organellar metabolite buffering, with implications for heme-related disorders and mitochondrial disease.

## Highlights

• Mitochondrial lncRNAs are enriched in G-quadruplex-forming sequences
• Mitochondrial lncRNA G-quadruplex structures directly bind and buffer heme
• MITO-PIP and MITO-PyPDS orthogonally disrupt lncRNA-mediated heme buffering
• Mitochondrial lncRNA G-quadruplexes regulate heme homeostasis and oxidative stress

## Graphical abstract

**Figure:**
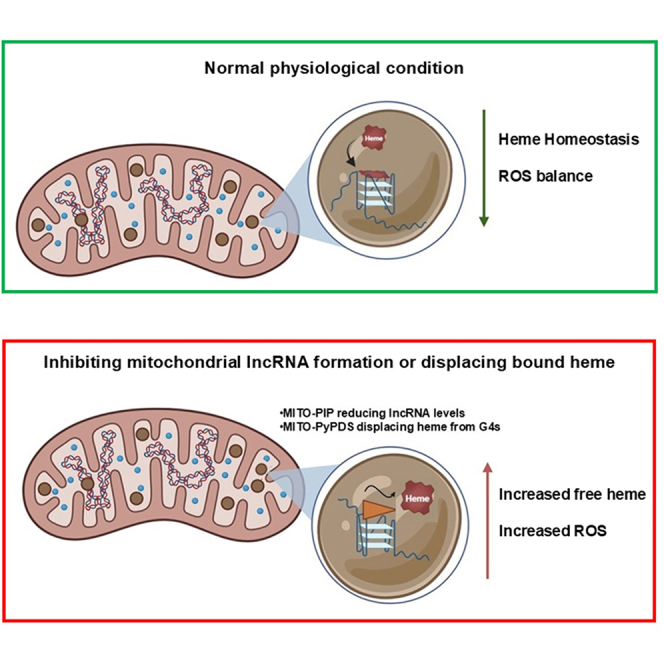

## Introduction

G-quadruplexes (G4s) are nucleic acid secondary structures composed of stacked “G-quartets” by guanine Hoogsteen-type hydrogen bonding and stabilized by ions such as potassium (K^+^) and sodium (Na^+^).^1^^,^^2^ The existence of G4 in the cell has been demonstrated using G4-specific probes, antibodies,^3^ and G4-sequencing,^4^ and G4 was found to be prevalent in the genome and transcriptome. The functional roles of nuclear DNA G4s have undergone substantial research over the past few decades and are thought to play a role in various cellular processes not limited to gene transcription,^5^ replication,^6^ genome stability,^7^ chromatin architecture,^8^ and phase separation.^9^ Compared to DNA G4s, RNA G4s (rG4s) are relatively less explored but are reported to play a role in translation,^10^ granule formation,^11^ and cell cycle progression.^12^ Most of the rG4s are identified in the open reading frame (ORF) and untranslated regions (UTRs) of protein-coding mRNA.^13^^,^^14^ The human genome is also extensively transcribed and produces non-coding RNA sequences longer than 200 nucleotides, termed “long non-coding RNAs” (lncRNAs).^15^ These lncRNAs have been implicated as important players in a range of biological processes, such as gene regulation, epigenetic control of chromatin structure, and membrane-less organelles.^16^ Recent studies reported the formation of rG4s in lncRNAs that have a regulatory role in gene expression and protein recognition.^17^^,^^18^^,^^19^

Mitochondria are best known for their classical roles in energy production but have other cellular functions such as calcium homeostasis, apoptosis, and stem cell generation.^20^^,^^21^ Unlike other organelles, mitochondria have their own genome with a size of nearly 17 kb and encode specific rRNAs, tRNAs, and mRNAs necessary for the synthesis of key respiratory complexes.^22^ Mitochondrial DNA (mtDNA) consists of two strands, heavy (H) and light (L), which are distinguished by their nucleotide composition. The guanine-rich H-strand acts as a template strand transcribing most of the mitochondrial transcripts. mtDNA analysis reveals many putative G4-forming sequences (putative quadruplex-forming sequences [PQSs]) with a density higher than that of nuclear DNA per kb.^23^^,^^24^ mtDNA G4 has also been observed using G4-targeting ligands in the cell and is thought to play an important role in mtDNA instability,^25^ mtDNA replication, transcription,^26^ and glycolysis.^27^ Despite studies reporting that most of the total lncRNA is of mitochondrial origin,^28^^,^^29^ their G4-forming abilities and function have remained heretofore unknown.

Heme *b* (iron protoporphyrin IX) is an essential molecule that is required for the majority of life and functions as an important protein cofactor and signaling molecule.^30^^,^^31^^,^^32^ However, since heme can also be cytotoxic, its concentration and bioavailability are tightly regulated in heme-requiring cells and organisms.^33^ The biosynthesis and degradation of heme are well understood,^34^ and in most metazoans, heme is synthesized in a highly conserved eight-step process in which the first reaction and the last three reactions take place in the mitochondria, and the remaining four take place in the cytosol.^35^ Once synthesized in the matrix side of the mitochondrial inner membrane, the mechanism and regulation of heme distribution, including its transport and trafficking, are poorly understood.^30^ Most studies on heme homeostatic factors have been centered on proteins, largely neglecting the potential role of nucleic acids. Indeed, nucleic-acid secondary structures, especially G4s, are known to interact with porphyrins such as heme with high affinity, exhibiting nanomolar dissociation constants for heme.^36^^,^^37^^,^^38^ Previous studies have reported heme sequestration by G4s within cells^39^ and heme buffering by human ribosomal rG4s.^40^ Recent studies have further expanded the toolkit for probing mitochondrial G4 structures, reporting small molecules capable of targeting G4s within mitochondria with improved selectivity.^41^^,^^42^ These advances underscore the growing recognition of mitochondrial G4s as druggable targets and the need to understand their functional roles. In this study, we provide evidence to show that heme is sequestered within mitochondria by a G4-forming lncRNA that originates from the mitochondria, thereby providing an explanation for how mitochondria handle large amounts of a potentially toxic but essential metabolite.

In this study, we first report the prevalence of rG4-forming sequences in mitochondria-generated lncRNAs (mt-lncRNAs) and thus investigate their potential biological function in regulating heme homeostasis. Using strand-specific qPCR, we found lncND5 and lncCytB to have high expression levels and utilized various spectroscopic methods to confirm the formation of rG4 structures. We then confirmed mitochondrial rG4 formation using RNA pull-down and bio-orthogonal imaging. To understand the functional role of mt-lncRNA, we investigated its potential role in buffering cellular heme; *in vitro* experiments demonstrated the binding of hemin to mt-lncRNA, and heme affinity agents demonstrated that heme interacts with mt-lncRNA *in vivo*. Further, inhibition of L-strand transcripts inside the mitochondria or heme displacement using G4 ligands targeted toward these organelles both resulted in elevated levels of buffered free heme, as assessed by genetically encoded heme sensors. The subsequent elevation in heme resulted in the induction of oxidative stress and heme oxygenase 1 (HMOX1), a key enzyme required for the detoxification and degradation of excess heme. Together, these results indicate that mitochondrial G4-rich mt-lncRNA binds and buffers heme, thereby potentially limiting heme toxicity inside the cell.

## Results

### Increased rG4-forming lncRNAs are generated from the L-strand in the mitochondrial genome

**Figure 1:**
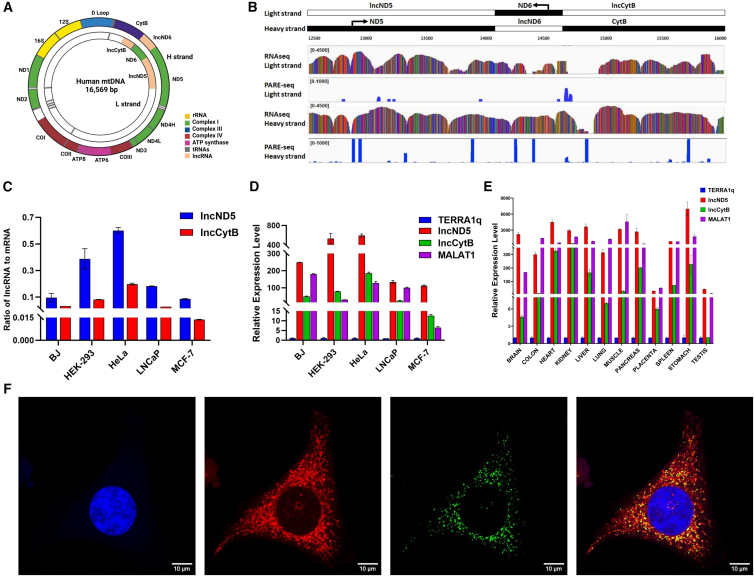
Expression of lncRNAs generated from the L-strand of the mitochondrial genome (A) Map of human mitochondrial DNA showing the presence of lncRNAs in both H- and L-strands. (B) Deep-sequencing analysis of RNA transcripts generated from H- and L-strands. (C) Ratio of lncND5 and lncCytB generated from the L-strand to their corresponding H-strand ND5 and CytB transcripts in various cell lines. (D) Relative expression levels of nuclear-encoded lncRNAs TERRA1q and MALAT1 and mitochondrial-encoded lncRNAs lncND5 and lncCytB in different cell lines. (E) Relative expression levels of nuclear-encoded lncRNAs TERRA1q and MALAT1 and mitochondrial-encoded lncRNAs lncND5 and lncCytB from total RNA of 12 different human tissues. (F) RNA fluorescence *in situ* hybridization (FISH) showing the localization of lncND5 in HeLa cells imaged by structured illumination microscopy. Nuclei stained with DAPI (blue), lncND5 (green), and mitochondria (red). Scale bars, 10 μm.

In the mitochondrial genome, the H-strand encodes most of the transcripts and has few noncoding sequences, and the L-strand is known to generate several lncRNAs along with ND6 mRNA and tRNAs (Figures 1A and 1B) We previously reported that the number of PQSs in the mitochondrial genome increases with organismal complexity.^24^ In this study, we sought to investigate the contribution of guanine-rich lncRNA to total mitochondrial G4s and their physiological roles. There is a considerable variation in the expression of lncRNA derived from the mitochondrial L-strand.^43^ Among them, lncND5 and lncCytB are among the most abundant mitochondrial lncRNAs^43^ (Table S2) and are arranged in a tail-to-tail manner with respect to their mRNA counterparts, suggesting a full-length transcript (Figure 1B). They also represent the most abundant fraction of G4 formation among the mitochondrial-generated RNA (Table S2), so we used them as a model in our study. To identify the PQS in lncND5 and lncCytB, we used the web-based server QGRS Mapper, which predicts quadruplex-forming G-rich sequences (QGRSs) in nucleotide sequences.^44^ It predicted 21 PQSs in lncND5 and 16 PQSs in lncCytB above the threshold levels required to form G4 structures, i.e., a minimum of two tetrads (Tables S3 and S4). We further evaluated the conservation of the G4-forming sequences in other species through comparative sequence analysis and found that the PQS was conserved with a high G4-forming threshold, (Figure S1), indicating a likelihood of its function.

To quantify the levels of lncND5 and lncCytB, we performed strand-specific RT-qPCR across various cell lines to discriminate against the H-strand transcripts. As shown in Figure 1C, in all tested cell lines, we found that lncND5 was more abundant than lncCytB, and the amount of lncND5 roughly corresponded to the amount of its antisense ND5 mRNA. We sought to investigate the levels of lncND5 and lncCytB in the cell relative to other highly expressed nuclear lncRNAs, including TERRA and MALAT1.^45^ As shown in Figure 1D, we found lncND5 to be more enriched compared to MALAT1 and lncTERRA. It is also noteworthy to mention that both TERRA and MALAT1 are reported to form rG4 structures.^17^^,^^46^ We continued the experiment across 12 different human tissues and generally found that mt-lncRNAs were relatively more enriched than both MALAT1 and TERRA (Figure 1E). More specifically, the relative ratio of mt-lncRNAs to nuclear-encoded lncRNAs was highest in cells from tissues demanding higher energy, such as heart, brain, and muscle. This led us to postulate that higher levels of mt-lncRNAs can be attributed to the higher mitochondrial copy number in these cells or their higher rate of transcription.

To identify the localization of lncND5, the highly abundant mt-lncRNA (Figures 1C and 1E), we performed fluorescence *in situ* hybridization (FISH) on HeLa cells using MitoTracker Deep Red and DAPI to visualize mitochondria and nucleus, respectively. Fluorescence was concentrated on the mitochondria, consistent with its mitochondrial origin. A more diffuse, lower level signal was also observed across the cytoplasm and nucleus (Figure 1F). To confirm that the apparent colocalization of lncND5 FISH signal with MitoTracker Deep Red does not arise from z-projection artifacts, we acquired full 3D z stacks and generated orthogonal (x-z and y-z) views. As shown in Figure S2, lncND5 FISH puncta co-occupy the same z-planes as the mitochondrial signal in both orthogonal projections, confirming true 3D colocalization. Quantitative Coloc2 analysis across *n* = 10 cells yielded a mean Pearson R of 0.66 ± 0.05, confirming significant mitochondrial enrichment. While recent evidence suggests that mt-lncRNAs may act as messengers between mitochondria and the nucleus,^47^ and while this observation could suggest that mt-lncRNAs may be exported from mitochondria, we acknowledge that these extra-mitochondrial signals were not validated with specificity controls, such as an lncND5 knockdown. Therefore, we cannot formally exclude the possibility that this diffuse signal represents a low level of off-target probe binding.

**Figure 2:**
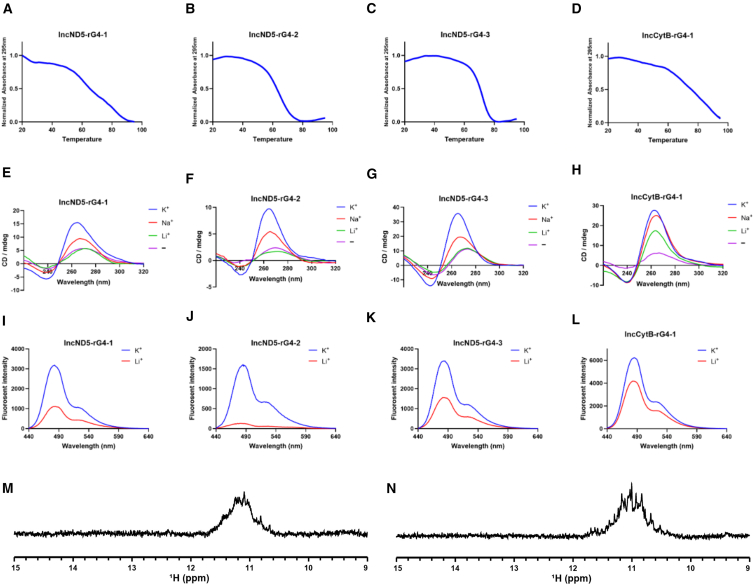
Biophysical studies of selected lncND5 and lncCytB rG4-forming sequences (A–D) UV melting of lncND5 rG4-forming sequences to determine thermal stability. The hypochromic shift observed indicates the formation of rG4 structures. (E–H) CD spectra of respective lncND5 rG4 sequences depicting characteristic parallel rG4 formation with absorption maximum at 264 nm and minimum at 240 nm. The signal strength was mostly in the order of K^+^ > Na^+^ >> Li^+^ > −, where − denotes RNA without metal ion. (I–L) Fluorescence intensity of rG4 sequences with Thioflavin T. The intensity of K^+^ was higher compared to Li^+^, indicative of rG4 formation. (M and N) ^1^H-NMR spectra of lncND5-rG4-1 (M) and lncND5-rG4-3 (N) in 20 mM potassium phosphate buffer (pH 6.5) containing 100 mM KCl. Imino proton signals in the 10.5–11.5 ppm region are characteristic of G-tetrad hydrogen bonding, confirming rG4 formation.

To evaluate the rG4 folding potential of lncND5 and lncCytB, we performed biophysical analysis on four selected PQS-forming RNA sequences (Table 1) based on their high G4-forming scores from the QGRS software. Using UV spectroscopy, we performed thermal denaturation of all four RNA oligonucleotides containing 150 mM KCl (G4-favoring environment) and LiCl (G4-non-favoring environment) at 295 nm. As shown in Figures 2A–2C, we observed a well-formed hypochromic sigmoidal curve with 150 mM KCl, a characteristic of G4,^48^ while LiCl displayed no characteristic curve at 295 nm. Our data further show high thermal stability, with T_m_ values indicating stable rG4 formation at standard physiological K^+^ concentration. Next, we performed circular dichroism (CD) spectroscopy to verify and determine the topology of rG4 secondary structures. The CD spectrum showed a characteristic positive peak at 264 nm and a negative peak at 240 nm (Figures 2E–2H), a characteristic signature of parallel G4 topology.^49^ The signal intensity of the peak also depended on the stability of the rG4 structure with ion and in the order of the K^+^ > Na^+^ >> Li^+^ > RNA alone as expected. Finally, we performed fluorescence light-up using Thioflavin T (ThT), which exhibits increased fluorescence intensity upon binding to G4 structures.^50^ As shown in Figures 2I–2L, we found an increased fluorescent intensity in all the sequences at 150 mM KCl compared to the same concentration of LiCl, as we expected, confirming the formation of rG4. We also confirmed that the rG4-mutant sequence cannot fold into rG4 using CD spectroscopy (Figure S3). To further validate G-tetrad formation, we performed ^1^H-NMR spectroscopy on selected lncND5-1 and lncND5-3 sequences. Characteristic imino proton signals in the 10.5–11.5 ppm region, characteristic of G-tetrad hydrogen bonding, were observed in both sequences under K^+^-containing buffer conditions (Figures 2M and 2N), providing direct spectroscopic evidence for rG4 formation. Together, all these results validated the stable formation of rG4 structures in the lncND5 and lncCytB sequences.

**Table 1:** Oligonucleotide sequences of lncND5 and lncCytB used for biophysical studies

Name	Length	Sequence	G4 score	T_m_
lncND5-rG4-1	20	GGAGUAGGGGCAGGUUUUGG	21	65°C
lncND5-rG4-2	16	GGGUGGUAAGGAUGGG	20	64°C
lncND5-rG4-3	26	GGGAAUUAGGGAAGUCAGGGUUAGGG	39	69°C
lncCytB-rG4-1	16	GGAUGGGGUGGGGAGG	20	77°C

### rG4 formation in the mitochondrial compartment in human cells

**Figure 3:**
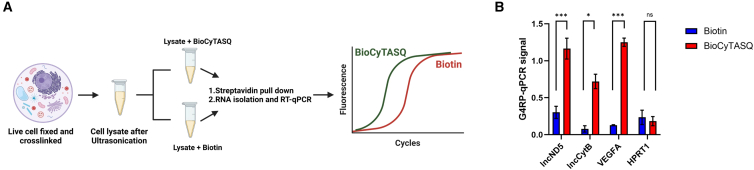
Mitochondrial lncRNAs form rG4 structures *in vivo* (A) Schematic workflow of BioCyTASQ pull-down. (B) G4RP-qPCR signal of BioCyTASQ versus biotin control for lncND5, lncCytB, VEGFA, and HPRT1 in HEK293 cells normalized to input control. Data are shown as mean ± SD (*n* = 3 biological replicates). ∗*p* < 0.05, ∗∗*p* < 0.01, ∗∗∗*p* < 0.001 by unpaired Student’s *t* test.

To further investigate the rG4 formation in the mitochondria, we performed both confocal microscopy and rG4 pull-down using BioCyTASQ, a biomimetic quadruplex ligand tagged with biotin.^51^ We used BioCyTASQ as it strongly prefers parallel rG4 topology and is expected to selectively enrich and transiently identify rG4 owing to its parallel-conformational constraints.^51^ Initially, we analyzed the G4-RNA-specific precipitation (G4RP) performed earlier using BioTASQ in the MCF7 cell line that is publicly available.^52^ As shown in Figure S4, we could see the enrichment of both lncND5 and lncCytB compared to control MALAT1, confirming the formation of rG4 *in vivo*. To further confirm the rG4 formation, we did the pull-down assay using BioCyTASQ in HEK293 cells using the workflow summarized in Figure 3A. We used VEGFA as a positive control as it was previously reported to form rG4 structures, and our enrichment was also in accordance with the earlier report.^52^ In agreement with the previous analysis, we found enrichment around (∼1- to 1.5-fold) in both lncND5 and lncCytB compared to the biotin control (Figure 3B). The negative control, HPRT1, which exhibits low G4-forming potential, did not show a significant change in the BioCyTASQ enrichment compared to biotin, confirming that the enrichment was indeed attributable to the rG4 structures.^51^ Together, our analysis and pull-down experiments confirm the rG4 formation by the lncRNAs from mitochondria.

Taking advantage of the biotin appendage, we used a bio-orthogonal approach to directly visualize the rG4 using AF488-conjugated streptavidin.^53^ After post-fixation with paraformaldehyde to interlock the rG4 and permeabilization using Triton X-100, the cells were incubated with BioCyTASQ for an hour followed by staining and visualization using confocal microscopy. This technique provides the landscape of G4 formation inside the cell. Most of the rG4-targeting chemical compounds are restricted to the nucleus in live-cell imaging.^54^ We observed a distinct pattern across the nuclear region (nucleoli, perinuclear) and a diffused signal in the cytoplasm. Importantly, we also found localization within the mitochondrial compartment (Figure S5). More recently, mitochondrial-penetrating G4 ligands indicated the formation of DNA G4s, suggesting a G4-favorable environment in these organelles.^27^ In order to gain further insight into the nature of the G4s, we fixed the HeLa cells and subjected them to both DNase and RNase treatments before BioCyTASQ labeling. As shown in Figure S5, while DNase I treatment did not affect the cytoplasmic and mitochondrial compartments, RNase A treatment induced a notable reduction in total fluorescence with only nucleoli sites remaining weakly fluorescent. Our observation was also similar to previously reported NaphthoTASQ (N-TASQ), a different class of TASQ series in MCF-7 cells.^54^ More specifically, we find that ∼20–30% of the total fluorescence signal localizes within mitochondria and the rest is presumably associated with the ribosomal rG4 fraction.^40^^,^^53^ Furthermore, RNase T1, which targets guanines in unfolded RNA regions, did not diminish the BioCyTASQ signals, demonstrating that the signal originates from rG4 folding (Figure S6). Overall, these results provide evidence of rG4 formation in mitochondria.

### Mitochondrial lncRNA rG4 structures bind hemin

**Figure 4:**
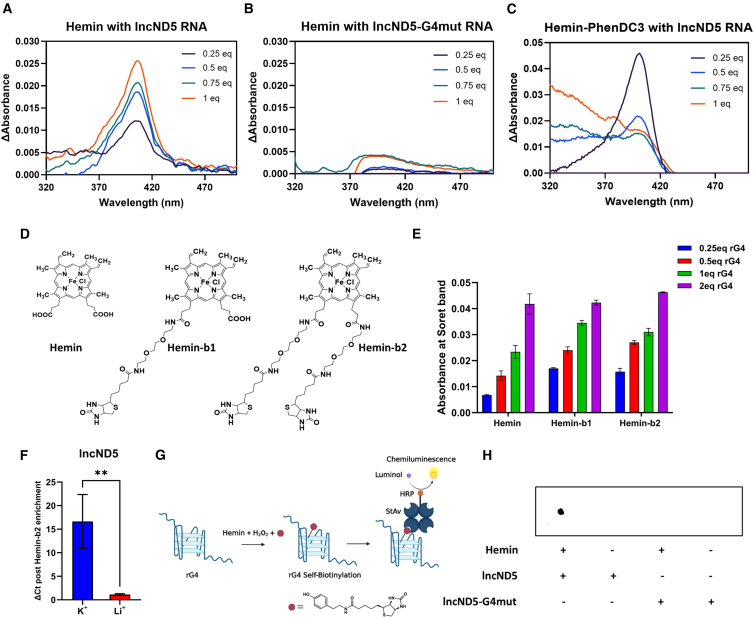
Mitochondrial lncRNA rG4 structures bind hemin (A) UV-vis ΔAbsorbance of hemin titrated with different equivalents of full-length lncND5 showing concentration-dependent Soret band enhancement indicating G4-hemin coordination. Wavelength (nm) is indicated on the *x* axis. (B) UV-vis ΔAbsorbance of hemin with lncND5 G4-mutant sequences showing minimal Soret band enhancement. Wavelength (nm) is indicated on the *x* axis. (C) UV-vis ΔAbsorbance of hemin with lncND5 in competition with Phen-DC3 showing concentration-dependent decrease in Soret band enhancement. Wavelength (nm) is indicated on the *x* axis. (D) Structures of hemin and its biotinylated derivatives Hemin-b1 and Hemin-b2. (E) UV-vis absorbance of Hemin, Hemin-b1, and Hemin-b2 with lncND5-rG4-1. (F) Post-enrichment of lncND5 after hemin-biotin pull-down experiment quantified by RT-PCR. (G) Schematic of self-biotinylation-based chemiluminescence of the hemin-G4 complex. (H) Dot blot showing signal from hemin and lncND5 RNA, indicating self-biotinylation of the G4-hemin complex. Data are shown as mean ± SD (*n* = 2 for E; *n* = 3 for F). ∗∗*p* < 0.01 by unpaired Student’s *t* test (F).

G4s are generally known to interact tightly with porphyrins like N-methylmesoporphyrin IX (NMM) and heme (Fe^3+^) through end-to-end stacking of the 3′ terminal G-quartets.^37^^,^^38^^,^^55^ Given that G4 structures were previously found to interact with and buffer heme *in vivo*,^39^^,^^40^ and our discovery that rG4 structures can form inside mitochondria, we investigated if mt-lncRNA binds heme. We first evaluated lncND5 binding interactions with hemin (Fe^3+^) using UV-visible (UV-vis) spectroscopy. We generated the full-length lncND5 using *in vitro* transcription (IVT) and confirmed its length by bioanalyzer. The RNA was annealed and then titrated into an aqueous buffered solution of hemin. As shown in Figure 4A, we observed a concentration-dependent enhancement of the Soret band (∼408 nm) arising from the Heme-G4 coordination. Further, we tested G4-mutant lncND5 RNA, which cannot fold into rG4 structures, and accordingly, absorption at the Soret band was not observed (Figure 4B), confirming that the Soret-band absorption is specific to the rG4-heme complex. Competition with Phen-DC3, a high-affinity G4-selective ligand that ends stacks similarly to hemin,^56^ resulted in a decrease in the Soret-band absorbance, consistent with hemin displacement from the G4-binding site (Figure 4C). Quantitative analysis of the ΔAbsorbance at 408 nm as a function of RNA concentration revealed a concentration-dependent Soret band enhancement that fitted well to a 1:1 specific binding model, yielding an apparent K_D_ of 1.73 μM (95% confidence interval [CI]: 0.78–4.29 μM; Bmax = 0.039; R^2^ = 0.993; Figure S7). In contrast, the G4-mutant control showed near-zero Soret band enhancement across all RNA concentrations (ΔA_408_ max = 0.004), approximately 7-fold lower than lncND5 rG4 at equimolar conditions, confirming strict G4-structure dependence of hemin coordination. We also observed the same phenomena of hemin interaction toward lncCytB, another G4-enriched RNA (Figure S8), while titration of hemin with the H-strand mRNA (ND5 and CytB) and 12s rRNA did not show any Soret band, indicating that the binding of hemin is mediated only by G4-enriched lncRNAs (Figure S9). Finally, we evaluated if the presence of the complementary mRNA can compromise the heme scavenging effect of lncND5 and lncCytB using competition experiments. As shown in Figure S10, in the presence of increasing equivalents of the complementary ND5 and CytB sequences, no major difference in the Soret band absorption was found. Overall, these results strongly support the binding of hemin with rG4 structures in lncND5 and lncCytB.

To further validate the ability of mt-lncRNA to bind hemin, we synthesized a biotinylated version of hemin with a linker length of 20 Å to provide sufficient space to interact with RNA. Our synthesis yielded two derivatives of hemin, one with a single-biotinylated hemin (Hemin-b1) and another with a double-biotinylated hemin (Hemin-b2) (Figure 4D). To confirm whether these biotinylated versions do not compromise hemin’s binding toward rG4 structures, we performed UV-vis spectroscopy. We used the rG4-forming oligonucleotide sequence lncND5-rG4-1 and evaluated its binding toward hemin and its biotinylated derivatives. As shown in Figure 4E, both Hemin-b1 and Hemin-b2 showed closer Soret-band absorbance compared to their unmodified counterparts, and we further proceeded with Hemin-b2 for pull-down experiments using total RNA. The total RNA was allowed to equilibrate with Hemin-b2 in both G4-favorable (K^+^) and G4-unfavorable (Li^+^) conditions for 2 h, and then each was subjected to enrichment using streptavidin beads. RNA was then eluted, and the relative abundance of each was quantified by RT-qPCR. As shown in Figure 4F, higher enrichment was observed in the G4-favorable condition, confirming that lncND5 can bind hemin within a sample cellular total RNA.

The G4-hemin complex is previously reported to self-biotinylate in the presence of biotin-tyramide and H_2_O_2_ via its intrinsic peroxidase activity.^57^ We took advantage of this technique to further evaluate the binding of hemin toward the lncND5 RNA. As illustrated in Figure 4G, a dot blot with a streptavidin-HRP conjugate was performed to test the covalent biotinylation of rG4-hemin. The biotinylation reaction was performed with both lncND5 and lncND5-mutant, each in the presence and the absence of hemin. After RNA cleanup, an equal concentration of RNA from each of the four conditions was loaded onto a nylon membrane; then, after crosslinking and development using streptavidin-HRP-mediated luminol oxidation, the result was inspected for chemiluminescence. As shown in Figure 4H, only full-length lncND5 in the presence of hemin showed chemiluminescence, whereas the G4-mutated RNA showed none regardless. Overall, our three independent approaches confirm the binding of hemin toward the mt-lncRNA rG4 structure.

### Heme regulation by lncRNA inside mitochondria

**Figure 5:**
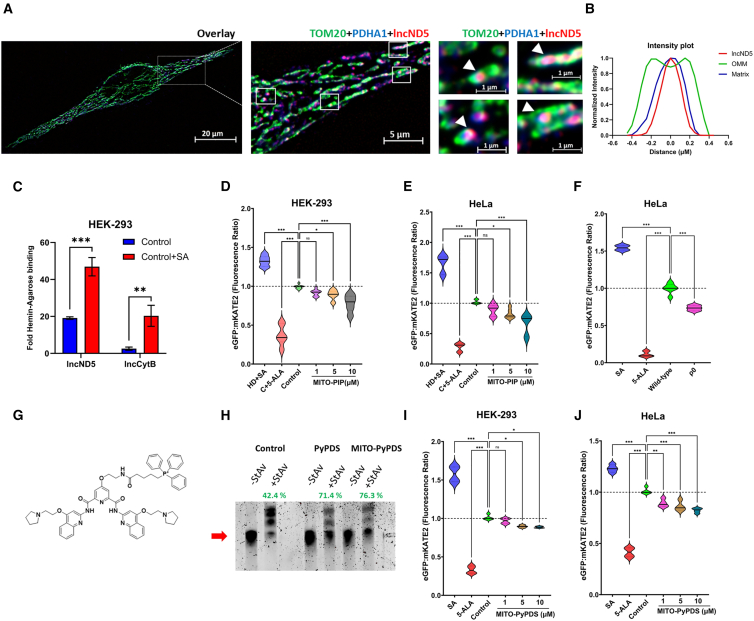
Mitochondrial lncRNA regulates heme inside the cell (A) Representative super-resolution structured illumination microscopy (SR-SIM) images of HeLa cells showing lncND5 localization. lncND5 was detected via RNA fluorescence *in situ* hybridization (FISH) (red), co-stained with the mitochondrial outer membrane protein TOM20 (green) and the mitochondrial matrix protein PDHA1 (blue) using a Zeiss Elyra 7 system. Left: whole-cell overview; scale bars, 20 μm. Middle: magnified regional view of the cropped network area (dashed box); scale bars, 5 μm. Right: high-magnification sub-mitochondrial zoomed views of selected areas (solid white boxes) highlighting localized puncta (white arrowheads); scale bars, 1 μm. (B) Normalized fluorescence intensity profiles (in arbitrary units) for each channel plotted over distance (*n* = 5 cells). (C) RT-qPCR of lncND5 and lncCytB in control and heme-depleted HEK293 cells after hemin-agarose binding; fold change is relative to Sepharose. HD + SA denotes heme-depleted fetal bovine serum (FBS) supplemented with 0.5 mM SA. (D and E) Flow cytometry analysis of the mitochondria-targeted heme sensor in HEK293 (D) and HeLa (E) cells. The *x* axis indicates treatment condition (C+5-ALA and MITO-PIP at indicated concentrations) for 24 h (48 h for HD + SA). The median sensor ratio was calculated as the fluorescence ratio of eGFP to mKATE2. (F) Flow cytometry analysis of the mitochondria-targeted heme sensor in HeLa and HeLa ρ0 cells (lacking mitochondrial DNA). The *x* axis indicates treatment condition (C+5-ALA) for 24 h. The median sensor ratio was calculated as the fluorescence ratio of eGFP to mKATE2. (G) Structure of MITO-PyPDS. (H) Native gel showing displacement of hemin by PyPDS and MITO-PyPDS, inhibiting biotinylation of the hemin-G4 complex in lncND5 partial-length RNA (red arrow). The upper band indicates the biotinylated G4 complex with added streptavidin (StAv). (I and J) Flow cytometry analysis of the mitochondria-targeted heme sensor in HEK293 (I) and HeLa (J) cells. The *x* axis indicates treatment condition (C+5-ALA and MITO-PyPDS at indicated concentrations) for 24 h (48 h for HD + SA). The median sensor ratio was calculated as the fluorescence ratio of eGFP to mKATE2. Data are shown as mean ± SD (*n* = 3 for C, F, I, and J; *n* = 4 for D and E). ∗*p* < 0.05, ∗∗*p* < 0.01, ∗∗∗*p* < 0.001, n.s. not significant (*p* > 0.05) by Student’s *t* test (C) or ordinary one-way ANOVA (D, E, F, I, and J).

In metazoans, heme is synthesized in a conserved eight-step biosynthetic pathway, with the final step being the insertion of ferrous iron into protoporphyrin IX on the matrix side of the mitochondrial inner membrane.^58^ Although mitochondria contain the most heme in the cell and have the highest demand for heme, very little is known about mitochondrial heme homeostatic mechanisms.^59^ Since our *in vitro* experiments indicate that heme can bind rG4 in mt-lncRNA and that mt-lncRNA is highly abundant, we investigated the role of rG4 mt-lncRNA in regulating mitochondrial heme. We assessed the distribution of lncND5 in the sub-mitochondrial structures using super-resolved structured illumination microscopy (SIM).^60^ To this end, after fixing HeLa cells, we targeted TOM20, a known outer-mitochondrial-membrane (OMM) protein, with anti-TOM20 for staining, and pyruvate dehydrogenase-α subunit 1 (PDHA1), which localizes to the mitochondrial matrix with anti-PDHA1 antibody, along with probes that target lncND5 (Figure 5A). The TOM20 staining appeared to be an elongated cylindrical-like structure enclosing each mitochondrion outer membrane and the PDHA1 signal localized to the matrix side, while the lncND5 appeared to be in a granule-like structure mostly localized to the matrix side of mitochondria colocalizing with PDHA1 (Figure 5B). Our observation of the lncND5 morphology was consistent with prior research, indicating that RNA granules were present in the matrix and inner mitochondrial membrane (IMM) fractions.^61^ Heme synthesis occurs in the IMM and the matrix fraction, and considering its localization, lncND5 has a considerable likelihood of interacting with heme.

To confirm the binding of heme with the lncRNA rG4 inside mitochondria, we took an approach using hemin-agarose, which has previously been used to show the interaction of ribosomal rG4 with heme inside cell.^40^ Within intact cells and cell lysates, biomolecules that are bound with heme would not interact with hemin-agarose resin since its heme-binding site is already occupied. Thus, the ability of a biomolecule to interact with hemin-agarose is inversely proportional to the amount of heme that is bound to it in cells.^62^ To confirm that rG4s can bind heme in cells, we depleted heme in HEK293 cells with 0.5 mM succinylacetone (SA), a specific inhibitor of the heme biosynthetic enzyme δ-aminolevulinic acid dehydratase,^63^ and accordingly observed a ∼3-fold reduction in total heme (Figure S14). The lysate of the control and the heme-depleted cells was quantified using bicinchoninic acid assay (BCA assay), with equal amounts being incubated with hemin-agarose and then subjected to strand-specific RT-qPCR to quantify the levels of lncND5 and lncCytB. Consistent with previous reports, we observed a nearly 2-fold increase within the heme-depleted cells, showing that a large amount of rG4 in the lncND5 and lncCytB became available to bind hemin-agarose (Figure 5C), while in the case of rG4-poor ND5 and CytB mRNA, there was no significant enrichment (Figure S15). These results confirm the interaction of rG4 with endogenous heme, specifically by the mt-lncRNA.

To systematically investigate the regulation of heme inside mitochondria by rG4, we deployed HS1, a ratiometric-fluorescent-based genetically encoded heme sensor. HS1 is a tri-domain fusion protein consisting of heme-binding cytochrome *b*_562_ (Cyt *b*_562_) fused with a pair of fluorescent proteins, eGFP and mKATE2. Heme is a fluorescence resonance energy transfer (FRET) acceptor of eGFP; hence, when it is bound to the Cyt *b*_562_ domain in HS1, eGFP fluorescence is quenched. Since the fluorescence of mKATE2 is unaltered by heme,^64^^,^^65^ the eGFP/mKATE2 fluorescence ratio of HS1 provides a readout of bioavailable heme. Using an HS1 variant targeted to mitochondria (HS1-mito), we found that fluorescence of eGFP increased as heme levels decreased (HD + SA). Furthermore, we observed decreased eGFP fluorescence when cells were given 5-aminolevulinic acid (5-ALA), a precursor of the heme synthesis pathway (Figures S16 and S17).

To investigate whether mt-lncRNA regulates heme, we used compounds developed in our lab: MITO-PIP, a mitochondria-penetrating peptide, conjugated with pyrrole-imidazole polyamides (PIPs), a sequence-recognition molecule that can selectively suppress promoter-specific transcription inside mitochondria.^66^ These compounds were designed to suppress the L-strand promoter (LSP) transcripts^66^ and also selectively alkylate mutant adenine in mtDNA.^67^ If mitochondrial G4-rich lncRNA buffers heme in the mitochondria, we would expect that inhibition of mt-lncRNA synthesis using L-strand transcriptional inhibitors would result in an increase in sensor-detectable heme due to disruption of a component of the mitochondrial heme buffer. To verify this hypothesis, we quantified the HS1-mito eGFP/mKATE2 fluorescence ratios using flow cytometry after treating the cells with different concentrations of MITO-PIP for 24 h. Before proceeding, we first confirmed the strand-specific inhibition of LSP transcripts using MITO-PIP by quantifying the lncND5, lncCytB with its respective mRNA in H-strand by strand-specific RT-qPCR (Figure S18). As indicated in Figures 5D and S19, the analysis of a population of ∼10,000 HEK293 cells shows a characteristic high median eGFP/mKATE2 fluorescence ratio in heme-depleted condition and low median eGFP/mKATE2 fluorescence ratio in heme-excessive condition compared to control cells. In the case of cells treated with MITO-PIP, the fluorescence ratio was quenched in a concentration-dependent manner, and these results were also reproduced in HeLa cells (Figures 5E and S20). We also evaluated the eGFP/mKATE2 fluorescence ratio using a control MITO-PIP compound as a negative control (that is not intended to bind the L-strand promoter) and found no significant changes with heme levels, further validating that the increased free heme is specific to inhibition of L-strand transcriptional (Figures S21–S23). To genetically validate that mitochondrially encoded transcripts, rather than other mitochondrial factors, are responsible for the observed heme regulation, we examined ρ0 cells that lack mitochondrial DNA and consequently lack all mt-lncRNA expression (Figures S24, S25, and S26). Heme sensors targeted toward wild-type and ρ0 cells indicated increased free heme inside the mitochondrial compartment (Figure 5F), and the ratio was consistent with the MITO-PIP, supporting a predominant role for mitochondrial RNA-based G4s in this regulatory network. Although ρ0 cells lack both mtDNA and mt-lncRNA, the predominant effect is attributed to lncRNA based on (1) higher abundance of lncRNA vs. mtDNA G4s and (2) the parallel topology of RNA G4s, which favors heme binding over DNA G4s. The predominant effect of lncRNA G4s was further verified using IMT1B, an allosteric mitochondrial RNA polymerase inhibitor.^68^ Treatment with various concentrations of IMT1B showed increased free heme (Figures S27 and S28), which was consistent with our MITO-PIP treatment.

Moreover, the change in heme occupancy of HS1-mito in HEK293 cells due to MITO-PIP is consistent with lncND5 rG4s being the primary heme buffer in mitochondria. Treatment with MITO-PIP increases HS1-mito heme occupancy from ∼35% to ∼70%, corresponding to a change in free heme from ∼1 to ∼7 nM (Figure 5D). The increase in free heme is expected based on the equilibrium model described in Figure S30 that assumes (1) a concentration of lncND5 rG4s spanning 20–800 μM, which is derived from estimates of lncND5 cellular copy number being 1,000 (Figure S29), mitochondrial volume spanning 0.04–0.08 fL,^69^ lncND5 having the potential to form up to 20 rG4s (Table S3), and the assumption that most lncND5 is mitochondrial; (2) total mitochondrial heme being ∼100 μM; (3) free and labile heme in HEK293 cells being largely oxidized^70^; (4) and the lncND5 rG4 heme dissociation constant being similar to other rRNA G4s^40^ (∼10 nM). As can be seen in Figure S30, which includes a plot of free heme as a function of total lncND5 rG4 concentration, a change in lncND5 rG4 from 1 to 0.2 mM results in an increase in free heme from ∼1 to 10 nM, which is on the order of estimates of the change in free heme associated with perturbations of lncND5 rG4 concentration. Together, these results are consistent with a role for G4-rich mt-lncRNA in buffering free heme levels since depletion of mt-lncRNA results in excess free heme. We further investigated heme levels in the nucleus and cytosol, using the HS1 heme sensors. First, we used a confocal microscope to confirm the localization of nuclear and cytosolic HS1 (Figures S31 and S32). As shown in Figure S33, upon inhibiting mt-lncRNA, we found an increased free heme in both the cytosol and the nucleus. We presume that this may be due to increased heme efflux from the mitochondria.

To further validate the heme buffering by mt-lncRNA, we synthesized MITO-PyPDS (Figure 5G), a version of small-molecule G4-binder, pyridostatin derivative (PyPDS) conjugated with lipophilic cation triphenylphosphonium (TPP) that can deliver the compound inside mitochondria. To validate the stabilization of G4 structures by MITO-PyPDS, we initially monitored the *T*_*m*_ profiles of the RNA using CD in the presence or absence of MITO-PyPDS (Figure S36). The comparison of melting temperature increase (Δ*T*_*m*_) demonstrated higher values (Δ*T*_*m*_ = 6.1°C) in the presence of the conjugate, confirming the stabilization of G4 structure by MITO-PyPDS. Subsequently, we evaluated the impact of the conjugate on rG4 recognition, and utilizing the ThT displacement assay, we verified that MITO-PyPDS could indeed bind to the G4 structures (Figure S37). To systematically investigate the MITO-PyPDS affinity toward the rG4 structures, we performed a biotinylation competition experiment as depicted in Figure S38. Since PyPDS is known to bind G4 structures by end-stacking at the terminal G-quartets like hemin, the addition of MITO-PyPDS is anticipated to displace the hemin. As expected, we found reduced streptavidin (StAv)-shifted gel bands upon the addition of PyPDS and MITO-PyPDS at 20 μM compared to the control without the addition of any ligand (Figure 5H), confirming the superior binding of MITO-PyPDS toward the rG4 structures. To provide direct evidence of the subcellular distribution of MITO-PyPDS, we synthesized a fluorescently tagged analog, TAMRA-MITO-PyPDS (Figure S35). Confocal microscopy of live HeLa cells treated with 1 μM TAMRA-MITO-PyPDS for 6 h, co-stained with MitoTracker Deep Red and Hoechst-33342, revealed preferential accumulation of the probe within the mitochondrial network, with a mean mitochondria-to-nucleus fluorescence ratio of 6.3 ± 2.4 (*n* = 9 fields; Figure S39). Near-zero colocalization with Hoechst-33342 (Pearson R = 0.08 ± 0.08) confirmed nuclear exclusion of the probe, indicating that nuclear G4 structures are not significantly perturbed at the concentrations used. Partial lysosomal accumulation was also observed, consistent with prior reports on PyPDS-based probes.^71^ Treatment of HEK293 (Figures 5I and S40) and HeLa (Figures 5J and S41) cells with MITO-PyPDS for 24 h resulted in a concentration-dependent decrease in the HS1-mito eGFP/mKATE2 fluorescence ratio, indicating an increase in mitochondrial free heme. Since MITO-PyPDS has high affinity toward G4 structures, we attribute the increase in free heme to displacement of heme from G4s inside mitochondria, which is also in line with our biotinylation competition experiment. The TPP moiety alone did not show any changes with the eGFP/mKATE2 fluorescence ratio, further validating the effect mediated by the G4 functional group (Figures S42, S43, and S44). Together, our results, using two independent chemical probes, provide strong evidence for a role of lncRNA rG4 structures in regulating heme. While these orthogonal chemical approaches provide robust convergent validation, future studies employing additional perturbation methods, such as antisense oligonucleotides targeting specific mt-lncRNAs, would further strengthen these conclusions.

While both mtDNA and mt-lncRNAs contain G4-forming sequences, multiple lines of evidence support the predominant role of RNA-based G4s in heme buffering. First, the RNase sensitivity of G4 signals in cellular imaging (Figure S5) demonstrates that the majority of detectable G4 structures are RNA dependent. Second, the convergent results from MITO-PIP (which specifically depletes mt-lncRNAs through transcriptional inhibition) and MITO-PyPDS (which competitively displaces heme from G4 structures) strongly implicate mt-lncRNA rG4s as the primary functional species. Third, comparison between wild-type and ρ0 cells, which lack all mitochondrial transcripts, reveals elevated free heme consistent with mt-lncRNA depletion by MITO-PIP and IMT1B. While we cannot completely exclude a minor contribution from mtDNA G4s, these combined observations establish that RNA-based G4 structures constitute the major functional pool for heme buffering in mitochondria. Overall, our three independent approaches confirm the binding of hemin toward the mt-lncRNA rG4 structures. While these experiments confirm a direct interaction, further quantitative studies, such as isothermal titration calorimetry or surface plasmon resonance, are needed to determine the precise binding affinity and kinetics of this interaction.

**Figure 6:**
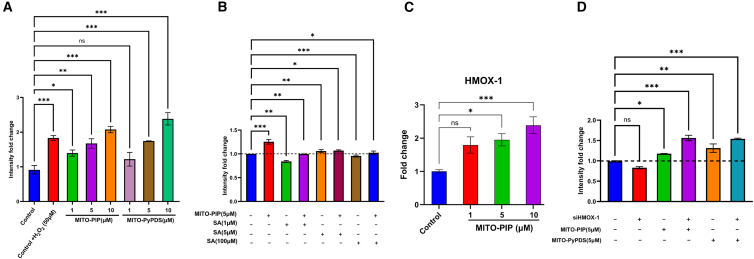
Functional significance of heme sequestration by mitochondrial lncRNA (A) ROS intensity fold change upon treatment with H_2_O_2_ at indicated concentrations; MITO-PIP and MITO-PyPDS at various concentrations in HEK293 cells for 24 h, measured by flow cytometry (1 h treatment for H_2_O_2_). (B) ROS intensity fold change in HEK293 cells treated for 24 h with various concentrations of SA and MITO-PIP, individually and in combination. (C) HMOX1 levels in HEK293 cells after treatment with different concentrations of MITO-PIP for 24 h. (D) ROS intensity fold change in the presence or absence of HMOX1 small interfering RNA (siRNA) and MITO-PIP at indicated concentrations. Data are shown as mean ± SD (*n* = 3 biological replicates). ∗*p* < 0.05, ∗∗∗*p* < 0.001, n.s. not significant (*p* > 0.05) by ordinary one-way ANOVA (A–D).

To investigate the potential consequences of elevated buffered free heme due to the perturbations to G4-rich mitochondrial lncRNA, we quantified the production of reactive oxygen species (ROS) using fluorogenic probe 2',7'-dichlorodihydrofluorescein diacetate (DCFDA). Indeed, hemin is known to catalyze the production of hydroxyl radicals through Fenton’s reaction.^72^ As anticipated, treatment with MITO-PIP at varying concentrations significantly elevated ROS levels in a concentration-dependent manner, and a similar effect was observed with treatment with MITO-PyPDS (Figures 6A and S45). To confirm that the observed ROS generation was attributable to increased free heme levels, we treated cells with varying concentrations of the heme synthesis inhibitor SA or depleted the heme-degrading enzyme HMOX1 using RNA interference. As shown in Figure 6B, the ROS levels induced by MITO-PIP were reduced in the presence of SA. In contrast, ROS levels induced by MITO-PIP were significantly elevated when HMOX1 was silenced (silencing was confirmed using qPCR; Figure S47). Treatment with MITO-PIP at 5 and 10 μM resulted in increased ROS levels in the HMOX-1 knockdown cells compared to the wild type (Figures 6D and S48), indicating the significance of HMOX-1 in controlling the ROS mediated by elevated free heme. These findings validate the role of free heme in driving ROS production upon inhibition of mitochondrial L-strand transcripts or altering the G4 environment inside mitochondria.

Since elevated heme levels are expected to induce the expression of the heme-degrading enzyme HMOX1, we also sought to investigate if inhibition of mt-lncRNA synthesis and the subsequent elevation in free heme would induce HMOX1. Using qPCR, we found a concentration-dependent increase in HMOX1 levels in HEK293 cells treated with MITO-PIP for 24 h (Figure 6C). These data are consistent with a previous study reporting that heme displacement from G4s in genomic DNA resulted in an upregulation of genes associated with heme degradation and iron homeostasis.^39^ To determine if the increase in free heme due to inhibition of lncRNA was due to changes in total heme, we quantified the total heme levels. As shown in Figure S49, MITO-PIP at different concentrations did not increase total heme; rather, if anything, it decreased total heme. By contrast, treatment with 5-ALA increased total heme levels by up to 3-fold in both HEK293 and HeLa cells as expected. We also evaluated the heme levels inside the mitochondria after purifying those organelles and found no increase in total heme (Figure S50), which is consistent with feedback inhibition of heme synthesis by elevated free heme or increased heme degradation by HMOX1.^73^ Altogether, the data indicate that the physiological consequences of perturbing the heme buffering capacity of mitochondrial G4-rich lncRNA are an elevation in free heme, which leads to an increase in oxidative stress and induction of heme detoxification mechanisms.

## Discussion

The physiological role of rG4s was unclear, primarily due to the lack of tools to perturb or visualize them. Indeed, most studies focused on its *in vitro* biochemical characterization, and their formation *in vivo* remained a debate.^74^ Recent advancements in G4-detection techniques suggest their transient formation inside the cell^13^^,^^75^^,^^76^ and further implicate its role in physiological and pathological conditions.^11^^,^^19^ While some nuclear-encoded lncRNA are reported to form rG4 structures that participate in transcription and protein regulation in cells,^17^^,^^18^^,^^77^^,^^78^ little is known about lncRNAs generated by mitochondria, even though mt-lncRNA contributes most of the total lncRNA.^28^^,^^29^ Herein, we report the abundance of mt-lncRNA compared to highly expressed nuclear-encoded MALAT1 and TERRA (Figure 1). We identified the high number of rG4 folding sequences in L-strand mt-lncRNA. Further, we confirmed the stable rG4 formation both *in vitro* using biophysical assays and *in vivo* using pull-down and bio-orthogonal imaging (Figures 2 and 3).

Our study demonstrates that lncRNAs with G4-rich sites may play important roles in heme homeostasis. Since lncND5 and lncCytB are highly expressed, we focused on them and confirmed their *in vitro* binding to hemin (Figure 4). Mitochondria, being the site of heme synthesis, must also protect itself from the toxicity of heme. While proteins such as progesterone receptor membrane component 2 (PGRMC2),^79^ TANGO2,^80^ and feline leukemia virus subgroup C receptor 1 (FLVCR1)^81^ are reported to traffic or export heme out of mitochondria, very little is known about what could buffer heme within the mitochondria or otherwise regulate its availability. In accordance with previous studies that report cellular RNA/DNA G4s sequestering heme,^39^^,^^40^^,^^82^ we also observed the same phenomenon within mitochondria, facilitated through mt-lncRNA rG4 structures. Inhibiting the L-strand from generating G4-rich lncRNA increased free heme primarily in mitochondria but also in other compartments in the cell. The importance of mt-lnc rG4 in heme buffering was further confirmed by treating cells with the mitochondrial-targeted G4 ligand, MITO-PyPDS, which resulted in elevated free heme inside those organelles, likely as a result of displacing the occupied heme sites in G4 structures (Figure 5). While our FISH imaging suggested that lncND5 may be present outside the mitochondria, we acknowledge, as noted in the results, that these extra-mitochondrial signals were not validated with specificity controls. Therefore, while the possibility of export is intriguing, these data must be interpreted with caution. Alternatively, it could be that mitochondrial rG4s regulate free heme levels in the mitochondria to gate its flow into other compartments through multiple parallel pathways of heme export from mitochondria.^83^ The elevated free heme further induced oxidative stress and increased the level of HMOX-1 to counteract the toxicity of free heme by degrading it (Figure 6).

Mitochondria descend from a bacterial ancestor more than a billion years ago and, during long-term evolution, the majority of the genes from the bacterial progenitor were lost or incorporated into the nuclear genome.^84^ However, during this evolution, the mitochondria genome went from being G4-poor to G4-rich,^23^ and given its deleterious effect on mtDNA stability, its increase raises important questions.^23^^,^^24^^,^^85^ Based on the demonstrated heme binding of G4s, we propose that it, at least in part, evolved to regulate mitochondrial heme homeostasis and availability. G4 evolution in mitochondria is also consistent with the evolution and conservation of rG4 sequences in rRNA, which was previously shown to regulate heme in the cytosol.^86^ Aside from lncRNA G4s binding and buffering heme, there may be roles for hemin-G4 complexes in biocatalysis. For instance, hemin-G4s exhibit peroxidase^36^ and catalase activities,^87^ but their physiological relevance remains unexplored. While the peroxidase activity of the hemin-G4 complex could potentially oxidize oxidation-sensitive guanine nucleotides,^36^ the catalase activity of the hemin-G4 scaffold inside the mitochondria could be beneficial for peroxide detoxification. In many cell types, mitochondria, which are major sites of peroxide generation due to electron leakage from the respiratory pathway, lack the peroxide-detoxifying enzyme catalase, thereby making it tempting to speculate that G4-hemin complexes can compensate for catalases.^88^^,^^89^

Another question remains as to what factors promote rG4 formation inside mitochondria. Currently, only one factor has been shown to regulate rG4 inside the mitochondria, the quasi-RNA recognition motif (qRRM) protein GRSF1. Interestingly, it is present only in vertebrates, which evolved to be G4-rich.^90^ The factors governing the formation of mitochondrial granules are not fully understood.^61^ While rG4 structures are shown to form a phase transition gel,^91^ it is conceivable the G4 structures in mt-lncRNA can contribute to granule formation as well. Recently, cellular response factors such as stress have been reported to increase rG4 formation in human cells.^92^ Additionally, K^+^ concentration inside the mitochondria was found to regulate mtDNA G4 formation.^27^ Proteins and other factors that can bind and/or regulate rG4 formation within the mitochondria should be the focus of future studies.

Mitochondrial heme regulation is critically influenced by the sequence and structure of RNA and DNA G4s. While both mtDNA and rRNA G4s can bind heme, rG4s typically adopt a parallel conformation, which facilitates heme binding due to its accessible ends, unlike the varied topologies of DNA G4s.^93^ Recent research further revealed that two-tetrad RNA G4s can form stable structures through dimerization, a capability not observed in DNA G4s.^94^ Furthermore, NMM, a compound structurally similar to hemin, was also shown to induce G4 formation.^95^ Together, these findings, in conjunction with our experimental evidence derived from heme sensor experiments comparing wild-type and ρ0 cells, strongly support the predominant role of lncRNA G4s in heme scavenging within mitochondria. It is also important to note that the stable three-tier G4 folding regions in the mitochondrial promoter region are critical for the transcription-replication switch,^96^ suggesting that heme may also potentially regulate mitochondrial transcription and replication. Finally, our findings are based on experiments in HEK293 and HeLa cells. While the consistency of the results between these two distinct cell lines suggests a potentially conserved mechanism, further studies in diverse primary cell types and, ultimately, *in vivo* models will be crucial to establish the broader physiological relevance and universality of this mt-lncRNA G4-mediated heme buffering pathway. The disease relevance of this mt-lncRNA rG4-mediated heme buffering mechanism is potentially broad. Dysregulated mitochondrial heme homeostasis is implicated in heme-related disorders including X-linked sideroblastic anemia and erythropoietic protoporphyria, as well as in neurodegenerative diseases where mitochondrial dysfunction and oxidative stress converge.^97^ Furthermore, given the elevated mitochondrial activity and heme demand in proliferating cancer cells, mt-lncRNA rG4 structures may represent promising therapeutic targets.

**Figure 7:**
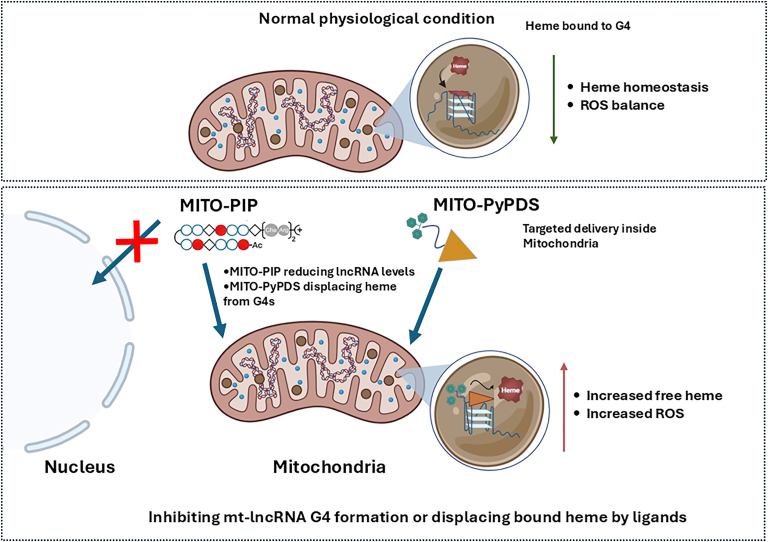
Schematic model of mt-lncRNA rG4-mediated heme buffering Under basal conditions, mt-lncRNA generated from mitochondrial L-strand transcription folds into rG4 structures that buffer labile hemin within the mitochondrial matrix, maintaining heme homeostasis and ROS balance. MITO-PIP, a mitochondria-targeted pyrrole-imidazole polyamide, selectively inhibits L-strand transcription, depleting mt-lncRNA rG4 structures and releasing buffered hemin into the labile pool. MITO-PyPDS, a mitochondria-targeted G4-binding ligand, competitively displaces hemin from rG4 structures through end-stacking competition. Both perturbations converge on elevated labile mitochondrial heme, increased ROS, and HMOX1 upregulation, validating the mt-lncRNA rG4 heme buffering model. Figure created using BioRender (BioRender.com).

In conclusion, we establish that mitochondrial-generated lncRNAs form rG4 structures both *in vitro* and in cells. Through orthogonal chemical perturbations, transcriptional depletion via MITO-PIP, and competitive displacement via MITO-PyPDS (Figure 7) combined with validation using ρ0 cells, we demonstrate that mt-lncRNA rG4s regulate heme homeostasis within mitochondria. The hemin binding we observed, together with the cellular experiments showing increased free heme, oxidative stress, and HMOX-1 induction upon rG4 disruption, reveals an RNA secondary structure-based mechanism for metabolite buffering. While direct visualization of these interactions in intact cells remains a technical challenge, our convergent evidence from *in vitro* binding assays and cellular functional studies strongly supports this model. This finding extends our understanding of G4 function beyond gene regulation to include direct metabolite sequestration, with implications for mitochondrial homeostasis, cellular metabolism, and diseases involving heme dysregulation.

### Limitations of the study

Several limitations of this study should be acknowledged. While bio-orthogonal imaging with BioCyTASQ and G4-specific pull-down provides cellular evidence for mt-lncRNA rG4 formation, these approaches report on bulk rG4 content and cannot distinguish individual G4-forming species or confirm that the observed signal arises specifically from lncND5 and lncCytB rather than other G4-forming transcripts or mtDNA. Although bioinformatic analysis identified G4-forming sequences across mt-lncRNAs, this study focused on lncND5 and lncCytB, and the full repertoire of functionally relevant rG4-forming sequences within the mitochondrial transcriptome remains to be characterized. The hemin-binding experiments were performed *in vitro*, and while cellular heme sensor experiments support the buffering model, direct quantification of rG4-heme interactions in the mitochondrial matrix *in vivo* awaits further methodological development. Selective knockdown of individual mt-lncRNAs was not achievable with current mitochondrial RNA interference tools, precluding loss-of-function validation at the single-transcript level. Finally, the present study was conducted in HEK293 and HeLa cell lines, and whether mt-lncRNA rG4-mediated heme buffering operates similarly in primary cells, tissue-specific contexts, or *in vivo* models remains to be established.

## Resource availability

### Lead contact

Further information and requests for resources and reagents should be directed to and will be fulfilled by the lead contact, Hiroshi Sugiyama (sugiyama.hiroshi.3s@kyoto-u.ac.jp).

### Materials availability

All unique/stable reagents generated in this study are available from the lead contact without restriction.

### Data and code availability

• NMR spectral data reported in this paper have been deposited at Mendeley data and are publicly available at https://doi.org/10.17632/7bg4nknfsk.1. NMR chemical shift and spectral data have also been deposited in the Biological Magnetic Resonance DataBank (BMRB) under accession number 53784 and are publicly available (https://doi.org/10.13018/BMR53784). Raw UV-vis absorbance data for Figures 4A–4C have been deposited at Mendeley data and are publicly available at https://doi.org/10.17632/7bg4nknfsk.1. All other data reported in this paper will be shared by the lead contact upon request.
• This paper does not report original code.
• Any additional information required to reanalyze the data reported in this paper is available from the lead contact upon request.

## Acknowledgments

This work was supported by 10.13039/100009619AMED (JP20am0101101 to H.S.), 10.13039/501100001691JSPS Grants-in-Aid for Scientific Research (23H02607 to H.S.), 10.13039/100000002NIH award GM145350 to A.R.R., a US National Science Foundation grant 1552791 to A.R.R., the 10.13039/100005200New Jersey Commission on Spinal Cord Research award (15IRG006) and NIH R21EY031439 to L.C., and the 10.13039/100020107Hirose Foundation for the Promotion of Science Research Grant (12th, FY2025) and 10.13039/100013328ROIS 10.13039/501100012311NII Open Collaborative Research 2026 (261S08-24153) to V.J.S. We acknowledge Fumiyoshi Ishidate, Dr. Takahiro Fujiwara, Dr. Takayuki Homma, Dr. Qiumei Akiyama, and Kumi Hidaka at Kyoto University for their support and technical assistance. We thank Jeffrey Friedl for proofreading the manuscript and Dr. Therese Solberg, Dr. Scott Behie, and Dr. Kaoru Kida Leong for their critical advice. We thank Kaori Hashiya for synthesizing the MITO-PIP for the research. We thank Dr. Yudai Yamaoki, Dr. Takashi Nagata, and Dr. Masato Katahira for NMR support and assistance. We thank Dr. Shingo Hirashima, Dr. Giordano Mattoni, Dr. Sefan Asamitsu, Dr. Yuki Hirose, and other lab members for discussions during the work. This work was supported by the World Premier International Research Center Initiative (WPI), 10.13039/501100001700MEXT, Japan. We acknowledge Prof. Kenya Honda for facilitating the research environment. Figures were created using BioRender (BioRender.com). BioCyTASQ was a kind gift from Dr. David Monchaud.

## Author contributions

Conceptualization, V.J.S. and H.S.; methodology, V.J.S., A.R.R., and H.S.; investigation, V.J.S., R.N., M.O., and T.T.; formal analysis, V.J.S., R.N., M.B.L., T.T., M.O., and S.S.; data curation, V.J.S., R.N., M.B.L., and T.T.; software, V.J.S., A.R.R., A.K., and L.C.; validation, V.J.S., M.B.L., and T.H.; resources, V.J.S., A.R.R., T.B., and T.H.; visualization, V.J.S.; organic synthesis, V.J.S., M.O., and T.B.; project administration, V.J.S.; writing – original draft, V.J.S.; writing – review and editing, V.J.S., A.R.R., and H.S.; funding acquisition, V.J.S. and H.S.

## Declaration of interests

The authors declare no conflicts of interest.

## Declaration of generative AI and AI-assisted technologies in the writing process

During the preparation of this work, the authors used ClaudeAI and Paperpal in order to enhance the readability and language of this work. After using this tool or service, the authors reviewed and edited the content as needed and take full responsibility for the content of the publication.

## STAR★methods

### Key resources table

**Table:** 

REAGENT or RESOURCE	SOURCE	IDENTIFIER
**Antibodies**		

Anti-TOMM20 primary antibody	Abcam	Cat# ab186734; RRID: AB_2716623
Anti-PDHA1 primary antibody	Abcam	Cat# ab110330; RRID: AB_10858459
Anti-rabbit AF488 secondary antibody	Abcam	Cat# ab150077; RRID: AB_2630356
Anti-mouse AF647 secondary antibody	Abcam	Cat# ab150115; RRID: AB_2687948

**Bacterial and virus strains**		

*E*. *coli* DH5α	Takara	Cat# 9057

**Chemicals, peptides, and recombinant proteins**		

DMEM (Dulbecco’s Modified Eagle’s Medium)	Gibco	Cat# 11965092
Fetal bovine serum (FBS)	Sigma-Aldrich	Cat# F2442
FastGene RNA premium kit	Nippon Genetics	Cat# FG-82302
PrimeScript II 1st strand cDNA Synthesis Kit	Takara	Cat# 6210A
PrimeScript RT reagent Kit	Takara	Cat# RR037A
THUNDERBIRD SYBR qPCR Mix	Toyobo	Cat# QPS-201
Human organ-specific total RNA (Premium Total RNA)	TAKARA	N/A
RNA oligonucleotides	Sigma-Aldrich/FASMAC	N/A (see Tables 1 and S1)
Nuclease-free distilled water	Ambion/Invitrogen	Cat# AM9937
Thioflavin T (ThT)	TCI	Cat# T0215
DSS (4,4-Dimethyl-4-silapentane-1-sulfonic acid)	Sigma-Aldrich	Cat# 613398
Hemin chloride	TCI	Cat# H0008
Hemin-Agarose	Sigma-Aldrich	Cat# H6390
Sepharose CL-4B beads	Sigma-Aldrich	Cat# CL4B200
EZ-Link Amine-PEG2-Biotin	Thermo Scientific	Cat# 21346
Hemin-b1 (mono-biotin-conjugated hemin)	This paper	N/A
Hemin-b2 (di-biotin-conjugated hemin)	This paper	N/A
BioCyTASQ	Gift from Dr. David Monchaud	N/A
MITO-PIP	This paper; Hidaka et al.^66^	N/A
Control-PIP	This paper; Hidaka et al.^66^	N/A
MITO-PyPDS	This paper	N/A
TAMRA-MITO-PyPDS	This paper	N/A
PyPDS	Synthesized per literature; Ooga et al.^98^	N/A
Biotinyl Tyramide	Sigma-Aldrich	Cat# SML2135
H2O2 (hydrogen peroxide)	Wako	Cat# 081-04215
Streptavidin-AF488	Thermo Fisher Scientific	Cat# S11223
Streptavidin-peroxidase conjugate	TCI	Cat# S0972
Streptavidin magnetic beads	Promega	Cat# Z5481
GelGreen nucleic acid stain	Biotium	Cat# 41004
5-Aminolevulinic acid (5-ALA)	Sigma-Aldrich	Cat# A3785
Succinylacetone (SA)	TCI	Cat# A0627
ROS Assay Kit -Highly Sensitive DCFH-DA- (H2DCFDA)	Dojindo	Cat# 340-09811
MitoTracker Deep Red	Thermo Fisher Scientific	Cat# M22426
Hoechst-33342	Thermo Fisher Scientific	Cat# H3570
DAPI Staining Solution	Abcam	Cat# ab228549
ProLong Gold Antifade Mountant	Thermo Fisher Scientific	Cat# P36930
PolyJet transfection reagent	SignaGen	Cat# SL100688
RNAiMAX	Thermo Fisher Scientific	Cat# 13778150
HMOX-1 siRNA	Thermo Fisher Scientific	N/A
Control siRNA	Thermo Fisher Scientific	N/A
Nylon membrane (Hybond-N+)	Cytiva/Amersham	Cat# RPN303B
Chemi-Lumi One Super	Nacalai	Cat# 02230
Oxalic acid	Wako	Cat# 151-00155
Pierce BCA Protein Assay Kit	Thermo Fisher Scientific	Cat# 23225
TRIZOL	Thermo Fisher Scientific	Cat# 15596026
MEGAscript T7 Transcription Kit	Thermo Fisher Scientific	Cat# AM1334
Quick Spin Columns (RNA purification)	Roche	Cat# 11274015001
DNase I	Takara	Cat# 2270A
RNase A	Thermo Fisher Scientific	Cat# EN0531
RNase T1	Thermo Fisher Scientific	Cat# EN0541
RNase inhibitor cocktail	Thermo Fisher Scientific	Cat# N8080119
Protease inhibitor cocktail	Nacalai	Cat# 25955-11
Qproteome Mitochondria Isolation Kit	Qiagen	Cat# 37612

**Critical commercial assays**		

Stellaris RNA FISH probes (lncND5, Quasar 570, 48 probes)	LGC Biosearch Technologies	Custom; Table S5
Stellaris RNA FISH Hybridization Buffer	LGC Biosearch Technologies	Cat# SMF-HB1-10
Stellaris RNA FISH Wash Buffer A	LGC Biosearch Technologies	Cat# SMF-WA1-60
Stellaris RNA FISH Wash Buffer B	LGC Biosearch Technologies	Cat# SMF-WB1-20
RNA Bioanalyzer High Sensitivity RNA Analysis kit	Agilent	N/A

**Deposited data**		

NMR spectral data of lncND5-rG4-1 and lncND5-rG4-3	Mendeley Data and BMRB	https://doi.org/10.17632/7bg4nknfsk.1. NMR chemical shift and spectral data have been deposited in the Biological Magnetic Resonance DataBank (BMRB) under accession number 53784.
Raw UV-Vis absorbance data (Figures 4A–4C)	Mendeley Data	https://doi.org/10.17632/7bg4nknfsk.1

**Experimental models: Cell lines**		

Human: HEK-293; female	JCRB	JCRB: JCRB1819
Human: HeLa; female	JCRB	JCRB: JCRB0011
Human: BJ; male	JCRB	N/A
Human: MCF-7; female	JCRB	JCRB: JCRB0134
Human: LnCaP; male	ATCC	ATCC: CRL-1740
Human: HeLa rho0 (mtDNA-depleted HeLa)	This paper; Spadafora et al.^99^	N/A

**Oligonucleotides**		

Primers and RNA oligo sequences used in this study	Sigma-Aldrich/FASMAC; see Table S1	Table S1
gBlocks gene fragment DNA templates (lncND5, lncND5-G4mut, lncCytB-G4mut)	Integrated DNA Technologies (IDT)	N/A

**Recombinant DNA**		

HS1-mito (mitochondria-targeted heme sensor)	Yuan et al.^100^	N/A
HS1-cyto (cytoplasm-targeted heme sensor)	Yuan et al.^100^	N/A
HS1-nuc (nucleus-targeted heme sensor)	Yuan et al.^100^	N/A
pMA3790 (mUNG1-EGFP, for rho0 cell generation)	Spadafora et al.^99^	N/A

**Software and algorithms**		

GraphPad Prism 9.5.1	GraphPad Software	https://www.graphpad.com
FlowJo v10.9	BD Biosciences	https://www.flowjo.com
ZEN Blue 3.4/ZEN 2014 (v9.1)	Zeiss	https://www.zeiss.com
Fiji/ImageJ with Coloc2 plugin	Schindelin et al.^101^	https://fiji.sc
QGRS Mapper	Kikin et al.^44^	http://www.bioinformatics.ramapo.edu/QGRS/
MEGA11	Tamura et al.^102^	https://www.megasoftware.net
IGV (Integrated Genome Browser)	Broad Institute	https://software.broadinstitute.org/software/igv/
Spectra Manager Suite	JASCO	N/A
ChemEQL v3.2.1	Muller, Eawag	https://www.eawag.ch/en/department/surf/projects/chemeql/

**Other**		

ZEISS Elyra 7 LSM SIM2 (super-resolution microscope)	Zeiss	N/A
ZEISS LSM 980 (confocal microscope)	Zeiss	N/A
JASCO J-805LST CD spectrometer	JASCO	N/A
JASCO FP-8300 spectrofluorometer	JASCO	N/A
JASCO V-650 UV-Vis spectrophotometer	JASCO	N/A
Bruker BioSpin AVANCE III HD 600 NMR spectrometer	Bruker	N/A
BD FACS Aria II flow cytometer	BD Biosciences	N/A
SpectraMax plate reader	Molecular Devices	N/A
LightCycler 480	Roche Diagnostics	N/A
μ-Slide 8 Well chamber	ibidi	Cat# 80826
Bioanalyzer (Agilent 2100)	Agilent	N/A
LAS 3000 imaging system	Fujifilm	N/A
GelDoc Go Gel Imaging System	Bio-Rad	N/A
Countess Automated Cell Counter	Invitrogen	N/A
MALDI-TOF/MS	Shimadzu	N/A

### Experimental model and study participant details

#### Cell lines

HeLa, HEK-293, BJ, MCF-7, and LnCaP cell lines were used in this study. HeLa, HEK-293, BJ, and MCF-7 were obtained from JCRB and LnCaP from ATCC. All cell lines were maintained in DMEM supplemented with 10% FBS at 37°C with 5% CO_2_. HeLa ρ0 cells lacking mitochondrial DNA were maintained in DMEM supplemented with 10% FBS, 50 μg/mL uridine, and 1 mM sodium pyruvate. This study did not involve human participants, vertebrate animals, or clinical samples. The cell line was not independently authenticated in our laboratory. All cell lines are tested for mycoplasma contamination.

### Method details

#### Deep-sequencing analysis

For the RNA-seq analysis, the sequencing data from HeLa cells was downloaded from the ENCODE project (http://genome.ucsc.edu/ENCODE/index.html) GSE22068 as fastq files. The fastq files were mapped with bowtie2 using the reference genome version hg18 index. The mapping required an exact match and was stored as a SAM file. The files were then converted to BAM files and further sorted and split based on the strand to get mitochondrial H and L-strand results. The files were then inspected and viewed in the Integrated Genome Browser https://software.broadinstitute.org/software/igv/using the human hg38 mitochondrial genome.

#### Strand-specific quantitative RT-PCR

RNA was isolated from the cell using the FastGene™ RNA premium kit (Nippon Genetics) according to the manufacturer’s manual. Human organ-specific total RNA (Premium Total RNA) was purchased from TAKARA. To quantify the level of coding and noncoding RNAs generated from a specific strand of mitochondrial DNA adapter sequence was incorporated with the strand-specific primer. After denaturation of RNA, approximately 200–300 ng at 65 °C, followed by reverse transcription using PrimeScript™ II 1st strand cDNA Synthesis Kit (Takara) using adaptor sequence containing primers at 42 °C for 60 min, followed by 70 °C for 15 min to inactivate the enzyme. The reverse transcribed cDNA was further quantified using qRT-PCR with a gene-specific primer designed to anneal the complementary sequence of the additional adapter sequence incorporated during reverse transcription. The primer sequences used for RT-qPCR are listed in Table S1. Quantitative PCR was performed in a LightCycler 480 (Roche Diagnostics) containing the reaction mixture along with SYBR Green mastermix (Toyobo) Subsequently, ^ΔΔ^CT was calculated after normalizing with the 18s rRNA control gene. The copy number of lncND5 was estimated by using a standard curve prepared from cDNA of determined concentration serially diluted after performing reverse transcription, as reported.^103^

#### G4-prediction

To predict the putative G4-forming sequences (PQS) in mitochondrial generated coding and non-coding RNA we used the QGRS mapper.^44^ The full-length sequence of the RNA sequence was entered to predict the G4-forming sequences with parameters that include a minimum G groups of 2, maximum length of 30, and a loop size from 0 to 36. The output sequences based on high G-score which tends to form G4 in lncND5 RNA were selected for *in-vitro* experiments. (Table 1) (Table S3 and S4). We acknowledge that G4 prediction algorithms have inherent limitations and that reliance on a single tool may introduce bias. However, QGRS Mapper is a widely-used algorithm for this purpose, and our subsequent *in vitro* biophysical experiments (Figure 2) were performed to experimentally validate the G4-forming potential of these predicted sequences.

#### G4-conservation analysis

The G4 conservation was performed after downloading the respective lncND5 mitochondrial sequences of different organisms from the UCSC genome browser. (https://genome.ucsc.edu/) The sequences were then aligned using ClustalW2 by MEGA11^102^ (https://www.megasoftware.net/) To determine the conservation in other organisms, the PQS from the human lncND5 sequences with the highest score served as a reference. Finally, the G4 score of the corresponding sequences of organisms was predicted using the QGRS mapper as mentioned in the G4-prediction method.

#### Fluorescent *in situ* hybridization

The fluorescent probes for lncND5 were designed using the online program Stellaris probe designer 2.0 (https://www.biosearchtech.com/support/education/stellaris-rna-fish) LGC Biosearch Technologies using the full-length RNA sequence as input. The output set of probes is designed for optimal binding properties to the target RNA sequence with a masking level of 4 with 48 probes of length 20 nucleotides conjugated with Quasar 570. The sequence of probes is given in Table S5. The single molecule FISH was performed as per the protocol suggested by Stellaris. Briefly, the HeLa cells are seeded in a μ-Slide 8 Well (ibidi), and after reaching the desired confluence the cells are stained using 500 nM MitoTracker Deep Red for 45 min at 37 °C. The cells are then fixed using 3.7% paraformaldehyde for 10 min in phosphate-buffered saline (PBS) after washing with 1 × PBS (Nacalai) two times. Further, the fixed cells were permeabilized using 0.3% Triton X-100 for 10 min and washed with 1 × PBS three times. For hybridization, the cells are initially washed with Stellaris RNA FISH Wash Buffer A after 5 min of incubation. The FISH probes were then added at a final concentration of 200 nM in Stellaris RNA FISH Hybridization Buffer and formamide (Thermo fisher) in each slide and the probes were left to hybridize at 37 °C overnight in the dark. After incubation, the hybridization buffer was removed at next morning and each well was incubated with 200 μL Stellaris RNA FISH Wash Buffer A for 30 min. The wash buffer was further removed, and the cells were stained with 100 μL of the nuclear stain DAPI (4,6-diamidino-2-phenylindole, 100 ng/mL) was added to each slide and left to incubate at 37 °C for 30 min in dark. Following DAPI removal the cells are incubated with 200 μL Stellaris RNA FISH Wash Buffer B for 5 min and removed. Finally, the cells are mounted with ProLong Gold Antifade Mountant (Thermo Fisher) The images were acquired using ZEISS Elyra 7 equipped with a PlanApo ×40/1.4 NA oil immersion objective with support of ZEN 2014 (version 9.1) and further processing was done using ZEN Blue 3.4.

#### RNA oligonucleotides

The RNA oligos used for the *in-vitro* experiments were purchased from Sigma and were dissolved into 100 μM concentration with nuclease-free distilled water (Ambion).

#### UV melting experiment

For UV melting measurement 5 μM oligonucleotides were prepared in 150 mM LiCl buffer and 150 mM KCl containing a total volume of 250 μL in Nuclease Free-H_2_O. The sample was then vortexed and heated to 95 °C for 5 min and allowed to cool at 25 °C at a rate of 1.0 °C/min for renaturation. After annealing the samples were loaded into a cuvette and the melting profiles were assessed by measuring the absorbance at 295 nm recorded from 15°C to 95 °C at a rate of 1.0 °C/min using a spectrophotometer V-650 (JASCO) with a thermo-controlled PAC-743R cell changer (JASCO) and a thermal circulator F25-ED (Julabo). The data was then blanked and smoothed using GraphPad Prism.

#### CD spectroscopy

The CD experiments were performed using a JASCO J-805LST spectrometer in a 1-cm path-length quartz cuvette. The RNA (5 μM) samples for CD titration were prepared in 10 mM phosphate buffer (pH 7.5) and 150 mM KCl/NaCl/LiCl, with a total volume of 250 μL in Nuclease-Free H2O. The sample was then vortexed, heated to 95 °C for 5 min, and allowed to cool at 25 °C at a rate of 1.0 °C/min in a thermo-controlled dry bath incubator for renaturation. CD spectra were then recorded at 25 °C over the range of 220–320 nm at a 1 nm interval using Spectra Manager Suite Spectroscopy Software. The data were blanked and normalized to mean residue ellipticity before being smoothed over 10 nm using Spectra Manager™ Suite and plotted using GraphPad Prism.

#### Fluorescence spectroscopy

The Fluorescence spectroscopy was performed using a JASCO spectrofluorometer FP-8300 in a 3 mm path-length quartz cuvette. The RNA (1 μM) samples were prepared in 10 mM phosphate buffer (pH 7.5) and 150 mM KCl/LiCl containing a total volume of 100 μL in Nuclease Free-H_2_O. The sample was then vortexed, heated to 95 °C for 5 min, and allowed to cool at 25 °C at a rate of 1.0 °C/min in a thermo-controlled dry bath incubator for renaturation. Thioflavin T (ThT) (TCI) at a final concentration of 1 μM was added to the mixture. After adding, the emission spectra were collected from 440 to 700 nm with an excitation wavelength of 425 nm at every 1 nm interval using Spectra Manager Suite Spectroscopy Software. A solution containing 10 mM phosphate buffer (pH 7.5) and 150 mM KCl/LiCl was used as a control, and a similar setup of the experiment was performed. The data was further analyzed and plotted using GraphPad Prism.

#### Nuclear magnetic resonance (NMR) measurements

RNA oligonucleotides were dissolved at a final concentration of 200 μM in 20 mM potassium phosphate buffer (pH 6.5) containing 100 mM KCl, 5% D2O and 10 μM 4,4-Dimethyl-4-silapentane-1-sulfonic acid (DSS). DSS was used as an internal chemical shift reference. Each sample solution was incubated at 95 °C for 5 min and cooled to 25 °C in 70 min. NMR spectra were recorded at 25 °C using a Bruker BioSpin AVANCE III HD 600 spectrometer equipped with a cryogenic probe with a Z-gradient.

#### G4RP-seq analysis

G4RP-seq analysis was performed according to protocol.^52^ Dataset GSE21975 was generated from the G4RP-seq from MCF-7 cells as fastq files were obtained from the NCBI portal.^51^ The fastq files were then mapped to the human reference genome (hg38) using hisat2 with default settings. The resulting sam files were then converted to bam files followed by bigwig files. The files were then inspected in the Integrated Genome Browser using the human mitochondrial genome (hg38) and viewed. The BioTASQ enrichment scores for the mt-lncRNAs and controls were identified by comparing the BioTASQ-treated sample versus the input control.

#### G4RP-pull down

G4RP-pull-down was performed as per G4RP.v2.^104^ Briefly, HEK-293 cells were seeded in a 175 cm^2^ dish, and after 70–80% confluence, the cells were washed with PBS followed by cross-linking with 1% formaldehyde for 5 min. After quenching the crosslinking with 0.125 M glycine for 5 min, the cells were trypsinized and resuspended in G4RP buffer (150 mM KCl, 25 mM Tris pH 7.4, 5 mM EDTA, 0.5 mM DTT, 0.5% NP40, RNase inhibitor cocktail (Thermo Fisher). The cells were then lysed using a needle-equipped syringe, followed by incubation with 100 μM BioCyTASQ or 100 μM biotin for negative controls overnight at 4 °C. The input control consisted of 5% of the sonicate, and the rest of the sample was incubated with 10 μg of streptavidin-magnetic beads (Promega) for 2 h at 4 °C. The beads were then washed with G4RP buffer and further incubated at 70 °C for 1 h to reverse crosslinking. The RNA was isolated using TRIZOL using a column according to the manufacturer’s protocol. The extracted RNA was reverse transcribed using PrimeScript™ RT reagent Kit (Takara) using an adaptor sequence containing primers in case of the mitochondrial transcript or random hexamer for VEGFA and performed as mentioned earlier for cDNA synthesis and qPCR-based quantification. The C(t) values of pull-down with BioCyTASQ or Biotin were normalized to the input control, and the fold change was calculated.

#### G4 bio-orthogonal imaging

G4 bio-orthogonal Imaging was done as reported earlier.^53^ Briefly HeLa cells are seeded in a μ-Slide 8 Well (ibidi) and after reaching desired confluence, the cells were stained using 500 nM MitoTracker Deep Red for 45 min at 37 °C. Next, the cells are fixed using 3.7% paraformaldehyde for 10 min in phosphate-buffered saline (PBS) after washing with 1 × PBS (Nacalai) for two times. Further, the fixed cells were permeabilized using 0.3% Triton X-100 for 10 min and washed with 1 × PBS three times. After fixation and permeabilization, the cells were treated with 1 μM BioCyTASQ for 1 h and washed 3 times with PBS, followed by incubation with 1 μg/mL Streptavidin-AF488 (Thermo) and 100 ng/mL DAPI. After 1 h, the stain was removed and cells were washed three times and mounted with ProLong Gold Antifade Mountant. The cells were then imaged using ZEISS LSM 980 equipped with a PlanApo 63×/1.4 NA oil immersion objective with support of ZEN 2014 (version 9.1) and further processing was done using ZEN Blue 3.4. For RNase and DNAse treatment the cells were incubated with DNase I (Takara) RNAse A (Thermo) for 5 h RNase T1 (Thermo) for 30 min at 37 °C before BioCyTASQ treatment. Fiji was used to determine fluorescence intensity.^101^

#### *In-vitro* transcription (IVT)

gBlocks gene fragment DNA templates for full-length lncND5 RNA, lncND5 G4-mut and lncCytB-G4-mut sequence were purchased from Integrated DNA Technologies (IDT) containing T7 promoter sequence. The gBlocks were centrifuged briefly and incubated at 50 °C for 20 min with Tris-EDTA buffer (pH 7.4) reaching a final concentration of 10 ng/μL. For the rest of the RNA IVT, DNA template strand was made by PCR using forward and reverse primers amplifying the target RNA sequence with the forward primer incorporated with T7 promoter sequence. The RNA was then transcribed using T7 polymerase MEGA script™ T7 Transcription Kit (AM1334) according to the manufacturer protocol. The fully transcribed RNA was further DNase treated and purified using Quick Spin Columns (Roche, 11274015001). The RNA product was verified with Bioanalyzer (Agilent) using RNA Bioanalyzer High Sensitivity RNA Analysis kit according to their protocol or by agarose gel. The RNA was stored in −30 °C for short term or lyophilised and stored in −80 °C for long term.

#### UV-vis spectral assays for heme binding

The full-length wild type and G4-mutant RNA (0.25, 0.5, 0.75, 1 equivalent concentration to hemin) were annealed by heating at 95 °C for 5 min and cooled to 25 °C at 1 °C/min containing 10 mM Tris-HCl (pH 7.5) and 150 mM KCl in Nuclease Free-H2O. After annealing hemin chloride was added to a final concentration of 3 μM bringing to a final volume of 20 μL and incubated for 30 min at room temperature. The samples were then added to a 384-well flat clear bottom microplate (PerkinElmer) and the absorbance was recorded from 200 to 700 nm using a Spectramax plate reader (Molecular Devices). Spectra in Figures 4A–4C are presented as ΔAbsorbance, calculated by subtracting the hemin-alone spectrum (3 μM hemin in assay buffer, no RNA) from each sample spectrum. Raw blanking spectra are provided in the Mendeley Data repository (see Data and Code Availability). No smoothing was applied to the data shown. For quantitative analysis of hemin–rG4 binding, the ΔAbsorbance at 408 nm was extracted for each RNA concentration and fitted to a 1:1 specific binding model (Y = Bmax × X/(Kd + X)) using GraphPad Prism (version 10). RNA concentrations were calculated from the molar equivalents relative to the fixed hemin concentration of 3 μM. The apparent dissociation constant (Kd) and maximum absorbance change (Bmax) were determined from the best-fit values with 95% confidence intervals reported.

#### Synthesis and characterization of biotin-conjugated hemin

The synthesis of biotinylated hemin is shown in Figure S11. Hemin (TCI) and Amine-PEG2-Biotin (Thermo Fisher) were dissolved in dimethylformamide (DMF) and conjugated using a combination of pentafluorophenyl diphenylphosphinate (FDPP) and N, N-diisopropylethylamine (DIEA) at room temperature overnight. The reaction was monitored an analytical high-performance liquid chromatography (HPLC) on a PU-2089 plus series system (JASCO) using COSMOSIL 150 × 4.6 mm 5C18-MS-II Packed Column (Nacalai Tesque, Inc.) in 0.1% TFA in water with acetonitrile as the eluent at a flow rate of 1.0 mL/min and a linear gradient elution of 0–100% acetonitrile in 40 min with detection at 254 nm. Accordingly, two biotin-conjugate hemin-b1 and hemin-b2 were formed, and their corresponding molecular weights were confirmed using MALDI-TOF/MS (Shimadzu), as shown in Figures S12 and S13. Finally, the biotin conjugates were purified using preparative HPLC (Jasco), and its purity was confirmed using HPLC and stored in dark until further use.

#### Quantification of hemin

The concentration of hemin and its conjugates was determined at 385 nm using an extinction coefficient of 5.84 × 10^4^ M^−1^ cm^−1^ .^105^

#### Hemin-G4 absorption assay

The lncND5-rG4-1 at different equivalents was annealed by heating at 95 °C for 5 min and cooled to 25 °C at 1 °C/min containing 10 mM Tris-HCL (pH 7.5) and 150 mM KCl in Nuclease Free-H_2_O. After annealing hemin chloride and its biotinylated conjugates were added to a final concentration of 3 μM and incubated for 30 min at room temperature. The samples are measured at 300–700 nm using a spectrophotometer V-650 (JASCO).

#### Biotinylated hemin pull-down

The biotinylated hemin pull-down experiments were performed using the total RNA from BJ cells. Briefly, the pre-folded RNA isolated from the BJ cells were incubated with 100 μM biotin hemin conjugates in G4-favorable (150 mM KCl, 25 mM Tris pH 7.4, 5 mM EDTA) and G4-unfavorable conditions (150 mM LiCl, 25 mM Tris pH 7.4, 5 mM EDTA) for 2 h at 4 °C with gentle rotation. After 2 h’ streptavidin magnetic beads were incubated together with the mixture at 4 °C. The supernatant was then removed by placing it in the magnetic stand. The pellet was then washed three times with a buffer with 5 min’ incubation between each wash. The hemin-bound RNA was extracted using TRIZOL and quantified using RT-qPCR. The C(t) values were used to calculate the relative RNA abundance in G4-favorable or G4-unfavorable conditions.

#### Self-biotinylation of rG4-hemin

Self-biotinylation of the rG4-hemin complex was performed with a slightly modified protocol reported earlier by Sen and colleagues.^106^ Briefly, 5 μg of full-length lncND5 or G4-mutant sequences were denatured for 3 min at 95°C followed by cooling to 25 °C in a buffer containing a final concentration of 150 mM KCl and 40 mM HEPES. Hemin (5 μM) was added to the mixture and incubated for 15 min to form a complex. 100 μM Biotinyl Tyramide (Sigma) was added and incubated for 15 min at room temperature followed by the addition of 1 mM H_2_O_2_ (Wako) for 5 min to biotinylate the complex. The reaction was stopped using quenching buffer (10 mM Ascorbate, 5 mM Trolox, and 10 mM Sodium azide) followed by purification using a column.

#### Dot blot

For dot blot, 50 ng of RNA samples were spotted on the Nylon membrane (Amersham Hybond™-N^+^, Cytiva), and after drying the membrane was cross-linked by baking at 80 °C for 2 h and washed two times in TBST (Tris-buffered saline with 0.1% Tween® 20 detergent) buffer. The membrane was blocked with 5% BSA for 1 h at 37 °C for 1 h and washed 2 times with TBST. Further, the membrane was incubated with streptavidin−peroxidase conjugate (TCI) at 1:2000 for 1 h at 37 °C, followed by 4 times TBST wash with 5 min’ incubation in each washing. Finally, luminol-based chemiluminescence detection was performed using Chemi-Lumi One Super (Nacalai) according to their protocol and imaged using LAS 3000 system. (Fujifilm).

#### Immunofluorescence

For immunofluorescence, the cells are seeded in μ-Slide 8 Well (ibidi) and grown to a confluence of 70–80% and the cells are fixed in 3.7% paraformaldehyde in PBS for 10 min after washing with 1× PBS (Nacalai) two times. The cell permeabilization and blocking was performed together by incubating the fixed cells containing 0.3% Triton X-100 and 10% pre-immune goat serum for 15 min. The cells were further washed with PBS 3 times and finally incubated with Stellaris RNA FISH Wash Buffer A for 5 min. The cells are then incubated with anti-TOMM20 primary antibody (ab186734, 1:200 dilution), anti-PDHA1 primary antibody (ab110330, 1:200 dilution) and 200 nM FISH probe in Stellaris RNA FISH Hybridization Buffer and formamide (Thermo Fisher) in each well, and the probes were left to hybridize at 37°C overnight in the dark. After overnight incubation, the cells were washed with PBST two times, followed by 30 min’ incubation with anti-rabbit AF488 secondary antibody (ab150077, 1:1000 dilution) and anti-mouse AF647 secondary antibody (ab150115, 1:1000 dilution). The secondary antibody conjugates were removed and cells were incubated with 200 μL Stellaris RNA FISH Wash Buffer B for 5 min. Finally, the cells are mounted with ProLong Gold Antifade Mountant (Thermo Fisher) and imaged using ZEISS Elyra 7 equipped with a PlanApo ×40/1.4 NA oil immersion objective with support of ZEN 2014 (version 9.1) and further processing was done using ZEN Blue 3.4.

#### Hemin-agarose binding

Hemin Agarose binding was performed as described earlier.^40^ Briefly HEK-293 cells were cultured in DMEM with 10% FBS in dish, and the media was replaced the next day (DMEM containing 10% heme-depleted FBS along supplemented 0.5 mm succinyl acetone) for heme depleted condition and DMEM with 10% regular FBS for control cells. After 24 h the cells were collected by trypsinization and lysed using 1.5-mm zirconium beads (Benchmark Scientific) according to their protocol and the lysate were quantified using BCA assay (Pierce™ BCA Protein Assay Kit). The Hemin-agarose (Sigma) and Sepharose (Sigma) beads were prepared by washing three times with lysis buffer (0.1% Triton X-100, 10 mm sodium phosphate, 150 mm KCl, 5 mM EDTA, pH 7.5, protease inhibitor cocktail (Nacalai) RNase inhibitor cocktail (Thermo)) After equilibration of beads equal amount of lysates 50 μg were loaded in both beads and allowed to bind for 60 min with gentle rotation. After incubation the beads were washed four times with lysis buffer (15-min incubation between each wash) followed by centrifugation. The final was consisted of 50 μL (1 M imidazole) in lysis buffer to elute the bead followed by centrifugation and the supernatant was collected. The RNA was isolated from the supernatant using TRIZOL according to their protocol. The fold enrichment was calculated by comparing the C(t) values of Hemin-agarose with Sepharose beads eluted fraction after performing the reverse transcription and qPCR as described in the earlier section.

#### Heme sensor transfection

HEK-293 and HeLa cells were seeded in a 6-well plate with 1 × 10^5^ cells in each well containing DMEM medium with 10% FBS. After 24 h the cells were transfected with Heme sensor plasmids targeting mitochondria, cytoplasm and nucleus compartment (HS1-mito, HS1-cyto, HS1-nuc)^70^ The transfection was carried out using PolyJet (SignaGen) with total volume of 100 μL serum-free DMEM containing 1 μg of plasmid DNA and 3 μL PolyJet reagent vortexed all altogether and incubated for 15 min for each well. Master mix was prepared according to the number of wells, and 100 μL of the mixture was then added to each well after changing with fresh media. For the Heme-depleted condition, the media was replaced with DMEM containing 10% heme-depleted FBS along with 0.5 mM succinylacetone. After 12 h the media was replaced and incubated for another 12 h followed by treatment with MITO-PIP/control-PIP at different concentration while control group had only vehicle DMSO (0.5%) The MITO-PIP was synthesised and purified as reported earlier in our lab^66^ and the concentration was measured using Nanodrop as reported earlier.^107^ For the heme excess condition cells were supplemented with 350 μm 5-Aminolevulinic acid (5-ALA) in DMEM followed by 24 h of incubation before carrying flow cytometry.

#### Heme sensor imaging

To confirm the localization of heme sensor in various compartments, HeLa cells were seeded in an μ-Slide 8 Well (ibidi) and the following day the cells were transfected using PolyJet as described in the earlier section with a reduced mixture in each well (0.2 μg DNA +10 μL serum-free DMEM +0.5 μL PolyJet) The media was changed before addition of the transfection mixture and in case of heme-depleted condition, the regular media was replaced with DMEM containing 10% heme-depleted FBS, along with supplemented 0.5 mM succinyl acetone. After 24 h, the media was changed again, and 350 μM 5-Ala was added for the heme excess condition. The cells were fixed with 3.7% paraformaldehyde for 10 min, followed by two PBS washes. The fixed cells were stained with DAPI for 40 min and mounted using ProLong Gold Antifade Mountant (Thermo fisher) and imaged using ZEISS Elyra 7 equipped with a PlanApo ×40/1.4 NA oil immersion objective with support of ZEN 2014 (version 9.1), and further processing was done using ZEN Blue 3.4.

#### Flow cytometry

To quantify the heme levels using heme sensor we performed Flow cytometer using BD FACS Aria II (BD Biosciences). After transfection and treatment of compounds as described in the previous section the cells were washed twice PBS and trypsinized. The cells were then collected after centrifugation and suspended in PBS and the clumps were removed using polystyrene tubes with cell strainer caps (Corning). Forward Scatter and Side Scatter was used for single cell population gating to remove data points obtained from debris and electrical noise. For fluorescent measurement the eGFP was excited using blue laser (ex 488 nm) and measured using a 530/30-nm bandpass filter, and mKate2 was excited using yellow-green laser (ex 561 nm) and measured using 610/20-nm bandpass filter. The data was analyzed using FlowJo v10.9 software using the FCS files exported from the instrument. The median eGFP/mKate2 fluorescence ratio was used to determine the heme levels in the cells. For more quantitative measurements, the fractional heme occupancy of the heme sensor is governed by Equation 1.

(Equation 1) $$\alpha =\%\;Heme\;Occupancy=R-R_{\min}/R_{\max}-R_{\min}$$

where *R* is the median sensor eGFP/mKATE2 fluorescence ratio under a given treatment condition, *R*_*min*_ is the median sensor eGFP/mKATE2 fluorescence ratio when heme is depleted from the sensor using succinylacetone, and *R*_*max*_ is the median sensor eGFP/mKATE2 fluorescence ratio when heme saturates the sensor using 5-ALA to increase heme biosynthesis.

The concentration of free heme can be calculated by Equation 2:

(Equation 2) $$\left[Heme\right]=\alpha \times K_{d}/100-\alpha$$

where *K*_*d*_ is the HS1-heme dissociation constant of 10 nM.

#### Generation of mammalian cells devoid of mitochondrial DNA (ρ0 cells)

The ρ0 HeLa cells were generated through persistent mtDNA damage induced by overexpression of the Y147A mutant uracil-N-glycosylase (mUNG1).^99^ Wild-type HeLa cells were seeded in 6-well plates and, after 24 h, transfected with pMA3790 encoding mitochondrially targeted mUNG1 containing EGFP for selection. After 48 h, the cells were collected via centrifugation, suspended in PBS, and sorted for GFP+ cells, which were subsequently plated on solid media for colony formation. The resulting colonies were isolated and further propagated. The ρ0 phenotype was confirmed in select clones by assessing their inability to grow in the absence of uridine and pyruvate. All tested putative ρ0 clones exhibited auxotrophy for uridine and pyruvate. The absence of mtDNA/mtlncRNA was further validated using qPCR (Figure S24) and confocal imaging (Figures S25 and S26). For mitochondrial DNA visualization, SYBR green was utilized at a 1:20,000 dilution and incubated for 5 min. Images were acquired following three washes in PBS. In the case of lncND5, FISH was performed as described in the “Fluorescent in situ hybridization” section.

#### Total heme quantification

The total heme was quantified using a protoporphyrin IX fluorescence assay.^108^ Heme stripping in FBS was done by treatment using 10 mM ascorbic acid, followed by dialysis against PBS for overnight, and filter sterilization using 0.1 μm syringe filter. The cells were grown in a 10 cm dish. The heme-stripped FBS, along with 0.5 mM succinylacetone (TCI) was used to deplete heme from cells.^100^ The control cells are grown in DMEM supplemented with 10% FBS. Accordingly, the cell pellet with the desired condition was collected after trypsinisation, and the number of cells was counted using Countess Automated cell counter (invitrogen). 2 × 10^6^ were harvested from each treatment condition and incubated with 250 μL of 20 mM oxalic acid at 4 °C, with gentle rotation overnight in the dark. Next, 250 μL 2 M oxalic acid was added, and the samples were split into two, with one incubated at 95 °C for 30 min and the other half left at room temperature. After incubation, the samples were centrifuged for 2 min at 21000g and added to a black 96-well microplate, and the fluorescence was measured at 400 nm excitation and 662 nm emission. (Glomax, Promega) The heme concentrations were calculated based on a standard heme curve over the range of 1–500 nM of hemin and processed as described for the cell suspensions. To identify the total heme level in the cell, the fluorescence of non-heat-treated fluorescence readings was subtracted from the heat-treated ones.

#### Mitochondrial total heme quantification

The mitochondrial total heme was quantified using a slightly modified method reported earlier, based on protoporphyrin IX fluorescence.^109^ To quantify the mitochondrial total heme, first the cytosolic and mitochondrial fractions are isolated using Qproteome Mitochondria Isolation Kit (Qiagen) according to the manufacturer’s protocol. The cells were grown and treated as mentioned in the above total heme quantification methodology, and after isolation of mitochondria from different samples, the mitochondrial fraction was resuspended with 200 μL of PBS containing 1% Triton X-100 and 1X Protease Arrest (Nacalai). Further 10 μL of from each fraction was quantified using the BCA assay to determine the protein concentration. The samples were then quantified for total heme with heme standards as mentioned in the previous section. The data was further normalized by dividing the amount of hemin in the sample by the amount of protein assessed by the BCA method. For instance, 50–100 μg of mitochondrial protein is required to quantify heme levels inside mitochondria.

#### Synthesis and characterization of MITO-PyPDS

The compound PyPDS (4-(2-Aminoethoxy)-N,N′-bis{4-[2-(1-pyrrolidinyl)ethoxy]-2-quinolinyl}-2,6-pyridinedicarboxamide) was synthesized according to literature procedure.^110^ The synthesis of MITO-PyPDS is shown in Figure S34. To A solution of PyPDS (5.1 mg, 7.24 μmol) dissolved in dry DMF (70 μL) (4-carboxybutyl)triphenylphosphonium bromide (5.1 mg, 11.5 μmol), PyBOP (7.7 mg, 14.8 μmol) in dry DMF (50 μL) and DIEA (5 μL, 28.7 μmol) was added. The mixture was kept shaken at room temperature for 2 h and monitored using an analytical HPLC on a PU-2089 plus series system (JASCO) using COSMOSIL 150 × 4.6 mm 5C_18_-MS-II Packed Column (Nacalai Tesque, Inc.) in 0.1% TFA in water with acetonitrile as the eluent at a flow rate of 1.0 mL/min and a linear gradient elution of 0–100% acetonitrile in 40 min with detection at 254 nm for target product. After validating the compound using MALDI-TOF/MS (Shimadzu) as shown in Figure S34 the mixture was poured into diethyl ether (40 mL) to produce an off-white precipitate. Finally, the compound was purified using preparative HPLC (Jasco) and its purity was confirmed using HPLC Figure S34 and stored at −30 °C.

#### ThT-displacement assay

To evaluate the G4 binding of MITO-PyPDS we performed ThT-Displacement Assay.^111^ Briefly the 1 μM RNA in 10 mM phosphate buffer (pH 7.5) and 150 mM KCl of 200 μL in Nuclease Free-H2O was vortexed and heated to 95 °C for 5 min and allowed to cool at 25 °C at a rate of 1.0 °C/min for renaturation with a thermo-controlled dry bath incubator. After annealing ThT was added at a final concentration of 0.5 μM mixed carefully and different concentration of MITO-PyPDS were added to the solution and incubated for 30 min. Finally, after incubation, the emission spectra were collected from 440 to 700 nm with an excitation wavelength of 425 nm at every 1 nm interval using Spectra Manager Suite Spectroscopy Software. A solution containing 10 mM phosphate buffer (pH 7.5) and 150 mM KCl/LiCl was used as a control and a similar setup of the experiment was performed. The data was further analyzed and plotted using GraphPad Prism.

#### CD melting assays

The CD melting was performed according to^98^ with RNA samples (5 μM, 100 μL) prepared in 10 mM Tris-HCl (pH 7.5) and 5 mM KCl buffer. Before analysis, samples were heated to 95 °C and cooled down to 25 °C on a 0.8 °C/min rate. Temperature scans were performed in the presence or absence of compound (15 μM, 0.5% DMSO) by monitoring continuously from 20°C to 95 °C at 267 nm on a 0.5 °C/min rate in a 1-cm quartz cuvette using a J-805LST spectrometer (JASCO) and a refrigerated and heating circulator F25-ME (Julabo). The T_m_ values were determined as the minimum of the first derivative of the sigmoidal approximate curve.

#### Biotinylation competition experiments

Biotinylation competition experiments were performed using a modified method reported earlier based on self-biotinylation of the Hemin-G4 complex.^106^ Briefly, the lncND5 partial-length RNA (1 μM) were denatured for 3 min at 95°C, followed by cooling to 25 °C in a buffer containing a final concentration of 150 mM KCl and 40 mM HEPES. Hemin (5 μM) was added to the mixture and incubated for 30 min to form a complex, followed by the addition of 20 μM PyPDS/MITO-PyPDS in the displacement groups. Followed by 500 μM Biotinyl Tyramide (Sigma) was added and incubated for 15 min at room temperature and a final addition of 1 mM H_2_O_2_ (Wako) for 30 min to biotinylate the complex. The reaction was stopped using catalase followed by purification using a column. After purification, the samples were equally separated in every group, and 50 μM Streptavidin solution was added and incubated for 10 min. The samples are then loaded into 8% native PAGE and run for 1 h at 100 V in 4 °C and stained using GelGreen (Biotium). The image was captured using GelDoc Go Gel Imaging system (Bio-Rad) and analyzed using Image Lab software.

#### Quantification of reactive oxygen species

HEK-293 cells at a density of 1×10^5^ were seeded in 12-well plates and incubated overnight. The cells were subsequently treated with compounds at specified time points and then treated with 5 μM H_2_DCFDA (Nacalai). The cells were incubated for 30 min in the dark and analyzed using flow cytometry. In the case of HMOX-1 silencing, siRNA treatment or control siRNA was transfected using RNAiMAX for 24 h, and the gene silencing was confirmed by RT-qPCR. After validation, cells were treated with HMOX-1 siRNA (10 nM) in the presence or absence of MITO-PIP at the indicated concentration, and ROS levels were measured after 24 h.

### Quantification and Statistical analysis

Statistical analyses were performed using GraphPad Prism (version 10). Data are presented as mean ± s.d. unless otherwise stated. The number of biological replicates (n) for each experiment is indicated in the corresponding figure legend. Statistical significance was assessed using unpaired Student’s *t* test for two-group comparisons and ordinary one-way ANOVA with multiple comparisons for experiments with more than two groups. Significance thresholds are defined as ∗*p* < 0.05, ∗∗*p* < 0.01, ∗∗∗*p* < 0.001; n.s. denotes not significant (*p* > 0.05). Binding isotherm fitting was performed in GraphPad Prism using a 1:1 specific binding model. Colocalization analysis was performed using the Coloc2 plugin in Fiji/ImageJ.
