# Supplementary material for: Mitochondrial-generated G-quadruplex-forming lncRNAs regulate heme homeostasis
Source: iScience. 2026 Jun 17;29(7):116425. doi: 10.1016/j.isci.2026.116425 (PMC13311188; doi:10.1016/j.isci.2026.116425)
Supplement: Document S1. Figures S1–S50 and Tables S1–S5 [file mmc1.pdf]

iScience, Volume *29*

Supplemental information

Mitochondrial-generated G-quadruplex-forming lncRNAs regulate heme homeostasis

Vinodh J. Sahayasheela, Ryohei Noizumi, Manendra B. Lankadasari, Takumi Terada, Mitsuharu Ooga, Atharv Kulkarni, Shinjiro Suzuki, Takuya Hidaka, Li Cai, Ganesh N. Pandian, Toshikazu Bando, Amit R. Reddi, and Hiroshi Sugiyama

Name	**Sequence** (5' -> 3')
hTERRA-RT	CCCTAACCCTAACCCTAACCCTAACCCTAA
h1qTERRA-F	GCATTCCTAATGCACACATGAC
h1qTERRA-R	ACCCTAACCCGAACCCTA
hMALAT1-F	GGGTGTTTACGTAGACCAGAACC
hMALAT1-R	CTTCCAAAAGCCTTCTGCCTTAG
lncND5-RT	GTCGTAAGATTACATAGCCGTCGTAGCCTTCTCCACTTC
lncND5- F	CATTGTTAAGGTTGTGGATGA
lncND5- R	GTCGTAAGATTACATAGCCG
ND5-RT	CAATAGGATAGTGCGCGAGAGTGTGATGCTAGGGTAGAA
ND5- F	CATTACTAACAACATTTCCCCCG
ND5- R	CAATAGGATAGTGCGCGAGA
lncCytB-RT	GTCGTAAGATTACATAGCCGGAGGCCAAATATCATTCTG
lncCytB- F	TAATGAAGGGCAAGATGAAG
lncCytB-R	GTCGTAAGATTACATAGCCG
CytB-RT	CAATAGGATAGTGCGCGAGAGTTGGCTAGGGTATAATTG
CytB-F	CCTATTCTTGCACGAAACGG
CytB- R	CAATAGGATAGTGCGCGAGA
ND5-IVT-F	TAATACGACTCACTATAGGGCCATGCACACTACTATAACCACCC
ND5-IVT-R	TAGGGTTAGGATGAGTGGGAAGA
lncCytB-IVT-F	TAATACGACTCACTATAGGG TGGAAAAAGGTTTTCATCTCCG
lncCytB-IVT-R	CCAATGACCCCAATACGCAAAA
CytB-IVT-F	TAATACGACTCACTATAGGGACCCCAATACGCAAAACTAACC
CytB-IVT-R	AGGCCCATTTGAGTATTTTGTTTTC
12s-IVT-F	TAATACGACTCACTATAGGGGGTTTGGTCCTAGCCTTTCTAT
12s -IVT-R	TGCACTTTCCAGTACACTTACC
h18S-F	AAACGGCTACCACATCCAAG
h18S-R	CCTCCAATGGATCCTCGTTA
HMOX-1-F	CCAGCAACAAAGTGCAAGATTC
HMOX-1-R	TCACATGGCATAAAGCCCTACAG
ATP6-F	GCCGCAGTACTGATCATTC
ATP6-R	CAGGTTCGTCCTTTAGTGTTG
r18S-F	ACGGACCAGAGCGAAAGCAT
r18S-R	GGACATCTAAGGGCATCACAGAC
lncND5 partial RNA	GGGGGAUUUUACAUAAUGGGGGUAUGAGUUUUUUUUGUU AGGGUUAACGAGGGUGGUAAGGAUGGGGGGAAUUAGGGA AGUCAGGGUUAGGGUGGUUAUAGUAGUGUGCAUGGUUAU UACUUUUAUUUGGAGUUGCACCAAAAU

Supplementary Table S1. List of Primers and Oligo sequence used in this study

	Human 143B cells, total RNA, whole mitochondria						
	H-strand			L-strand			
rRNA/mRNA	Read frequency (rpkm)	G4 forming sites	Fraction of G4 formation (%)	rpkm	G4 forming sites	Fraction of G4 formation (%)	antisense/sense expression ratio
12S.rRNA	**292508.4**	**1**	**1%**	**0.0**	**7**	**5%**	**0.000**
16S.rRNA	**355921.3**	**1**	**1%**	**0.0**	**12**	**8%**	**0.000**
ATPase8/6	**5745.9**	**0**	**0%**	**452.1**	**10**	**7%**	**0.079**
CO1	**13495.3**	**1**	**1%**	**287.7**	**15**	**10%**	**0.021**
CO2	**13890.2**	**0**	**0%**	**595.9**	**7**	**5%**	**0.043**
CO3	**6530.3**	**1**	**1%**	**756.1**	**7**	**5%**	**0.116**
Cytb	**3759.4**	**0**	**0%**	**3590.8**	**16**	**11%**	**0.955**
ND1	**4003.9**	**0**	**0%**	**455.5**	**14**	**9%**	**0.114**
ND2	**5343.3**	**0**	**0%**	**377.5**	**11**	**7%**	**0.071**
ND3	**9536.0**	**0**	**0%**	**2234.7**	**2**	**1%**	**0.234**
ND4L/4	**4971.4**	**0**	**0%**	**1209.4**	**13**	**9%**	**0.243**
ND5	**3425.3**	**0**	**0%**	**2141.5**	**22**	**15%**	**0.625**
ND6	**2621.4**	**0**	**0%**	**1395.6**	**7**	**4%**	**0.532**

Supplementary Table S2. Expression of mRNA, lncRNA, and rRNA in 143B cells[1] whole mitochondria (reads per million per kb). The expression levels relative to total mitochondrial gene expression, and the ratio of sense/antisense transcription relative to each gene, G4-forming sites, and their fraction of formation.

Position	Length	QGRS	G-Score
49	25	GGTGATGATGGAGGTGGAGATTTGG	18
126	20	GGAGTAGGGGCAGGTTTTGG	21
156	23	GGCCTAGATAGGGGATTGTGCGG	13
256	23	GGTTAGGTAGTTGAGGTCTAGGG	17
324	23	GGTAGAGGGGGATTGTTGTTTGG	12
349	14	GGGGGATGCGGGGG	18
396	27	GGCTTCCGGCTGCCAGGCGTTTAATGG	18
423	17	GGTTTAGTAGGGTGGGG	14
463	15	GGTGGGGAAGCGAGG	15
648	21	GGCTGCCAATGGTGAGGGAGG	15
732	21	GGTTGTGGATGATGGACCCGG	20
801	20	GGAATGCTAGGT<br>GTGGTTGG	16
984	14	GGGGTGGAAGCGG	18
1038	24	GGAGTAGGGCTGAGACTGGGGTGG	16
1062	28	GGCCTTCTATGGCTGAGGGGAGTCAGGG	19
1145	14	GGGGTGAGGCTTGG	18
1549	26	GGAGAGCTGGGTTGTTTGGGTTGTGG	19
1605	29	GGTCTAGGCACATGAATATTGTTGTGGGG	4
1654	30	GGATGCGACAATGGATTTTACATAATGGGG	9
1713	16	GGGTGGTAAGGATGGG	20
1729	26	GGGAATTAGGGAAGTCAGGGTTAGGG	39

Supplementary Table S3. Potential rG4-forming sequences in the lncND5 RNA

Position	Length	QGRS	G-Score
100	25	GGAGATAGTTGGTATTAGGATTAGG	17
213	17	GGAGGTCTGCGGCTAGG	17
288	11	GGAGGATGGGG	19
309	29	GGATGAGGATGGATAGTAATAGGGCAAGG	16
361	28	GGATCGGAGAATTGTGTAGGCGAATAGG	13
400	30	GGGCTTGATGTGGGGAGGGGTGTTTAAGGG	37
453	19	GGTCGCCTAGGAGGTCTGG	15
546	30	GGTGGAAGGTGATTTTATCGGAATGGGAGG	16
618	20	GGAGGTGGAGTGTTGCTAGG	12
732	27	GGTCTGTCCCAATGTATGGGATGGCGG	7
801	30	GGCCTCACGGGAGGACATAGCCTATGAAGG	9
949	25	GGCGCCATTGGCGTGAAGGTAGCGG	18
1024	18	GGCGGTTGAGGCGTCTGG	17
1080	15	GGAGGATCAGGCAGG	18
1135	16	GGATGGGGTGGGGAGG	20
1185	28	GGTTAGTTTTGCGTATTGGGGTCATTGG	6

Supplementary Table S4. Potential rG4-forming sequences in the lncCytB RNA

Probe #	Probe (5'-> 3')	Percent GC
1	ctagacctaacctgactaga	45.00%
2	cttcttacgagccaaaacct	45.00%
3	aacatactcggattctaccc	45.00%
4	tagacctcaactacctaacc	45.00%
5	ttcctaggacttctaacagc	45.00%
6	tctacctaaaactcacagcc	45.00%
7	ttactaacaacatttccccc	40.00%
8	aagcctattcgcaggatttc	45.00%
9	taaaccccattaaacgcctg	45.00%
10	taacgaaaataaccccaccc	45.00%
11	taacaggtcaacctcgcttc	50.00%
12	cgcctatagcactcgaataa	45.00%
13	ctattactctcatcgctacc	45.00%
14	tcatacacaaacgcctgagc	50.00%
15	ccacatcatcgaaaccgcaa	50.00%
16	tcacaggtttctactccaaa	40.00%
17	gcattagcaggaataccttt	40.00%
18	ggactactcaaaaccatacc	45.00%
19	ccatcatccacaaccttaac	45.00%
20	ccttcttcaaagccatacta	40.00%
21	agttacaatcggcatcaacc	45.00%
22	ttctccacttcaagtcaact	40.00%
23	tgcgcccttacacaaaatga	45.00%
24	tatcaccactctgttcgcag	50.00%
25	ctctaacactatgcttaggc	45.00%
26	ctagcagaaaatagcccact	45.00%
27	ggaatcttcttactcatccg	45.00%
28	tccactcaagcactatagtt	40.00%
29	aaatcagcccaattaggtct	40.00%
30	ctaaacgctaatccaagcct	45.00%
31	gagacccacaacaaatagcc	50.00%
32	tacaaccgtatcggcgatat	45.00%
33	agcagatgccaacacagcag	55.00%
34	tcatcagttgatgatacgcc	45.00%
35	gcgtaggaattatatccttc	40.00%
36	caactgttcatcggctgaga	50.00%
37	ttagttaccgctaacaacct	40.00%
38	actcatcttcctaattacca	35.00%
39	cccaaacattaatcagttct	35.00%
40	cgttacatggtccatcatag	45.00%
41	aatattcatccctgtagcat	35.00%
42	gaactgacactgagccacaa	50.00%
43	gtgcctagaccaagaagtta	45.00%
44	tcttccccacaacaatattc	40.00%
45	gcatccacctttattatcag	40.00%
46	tcatacccccattatgtaaa	35.00%
47	taaccctgacttccctaatt	40.00%
48	catgcacactactataacca	40.00%

Supplementary Table S5. FISH probes designed for the lncND5 RNA used in the study

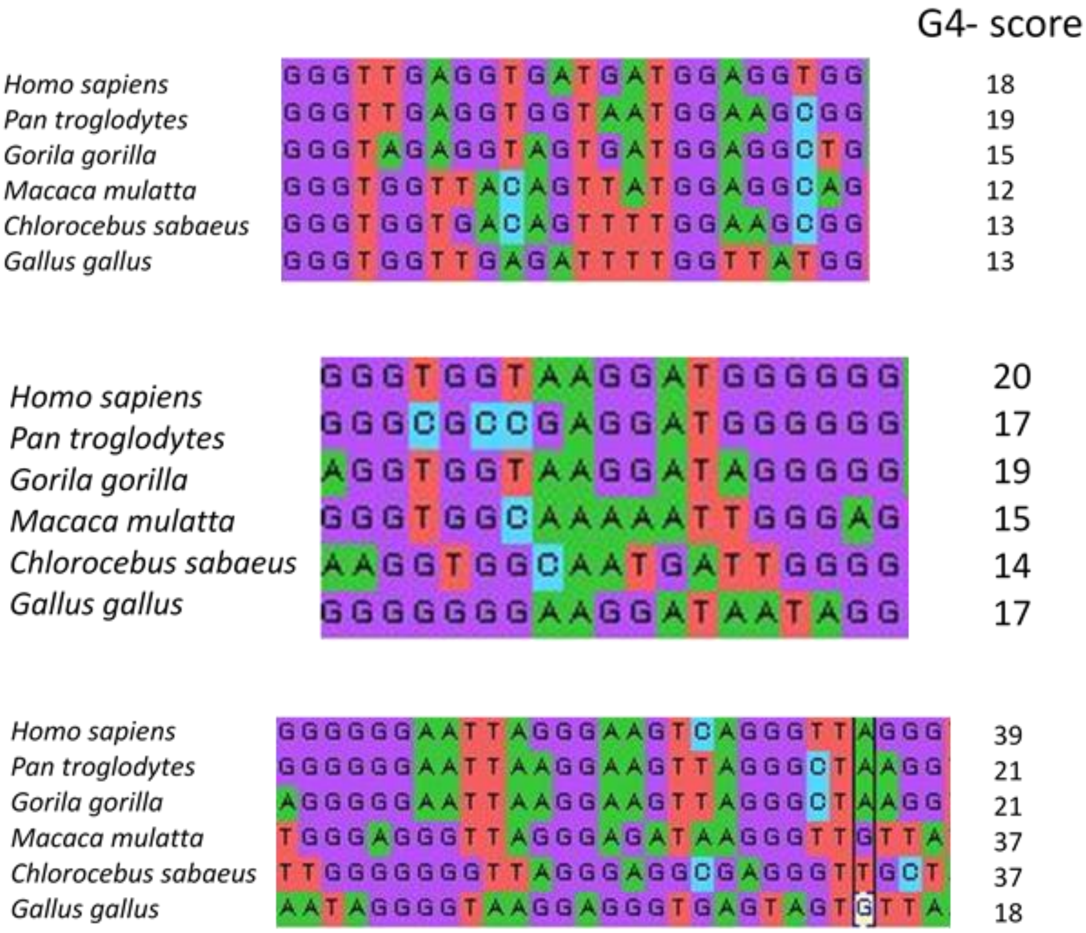

Supplementary Figure S1. Analysis of selected lncND5 sequences in different species showing the rG4 conservation.

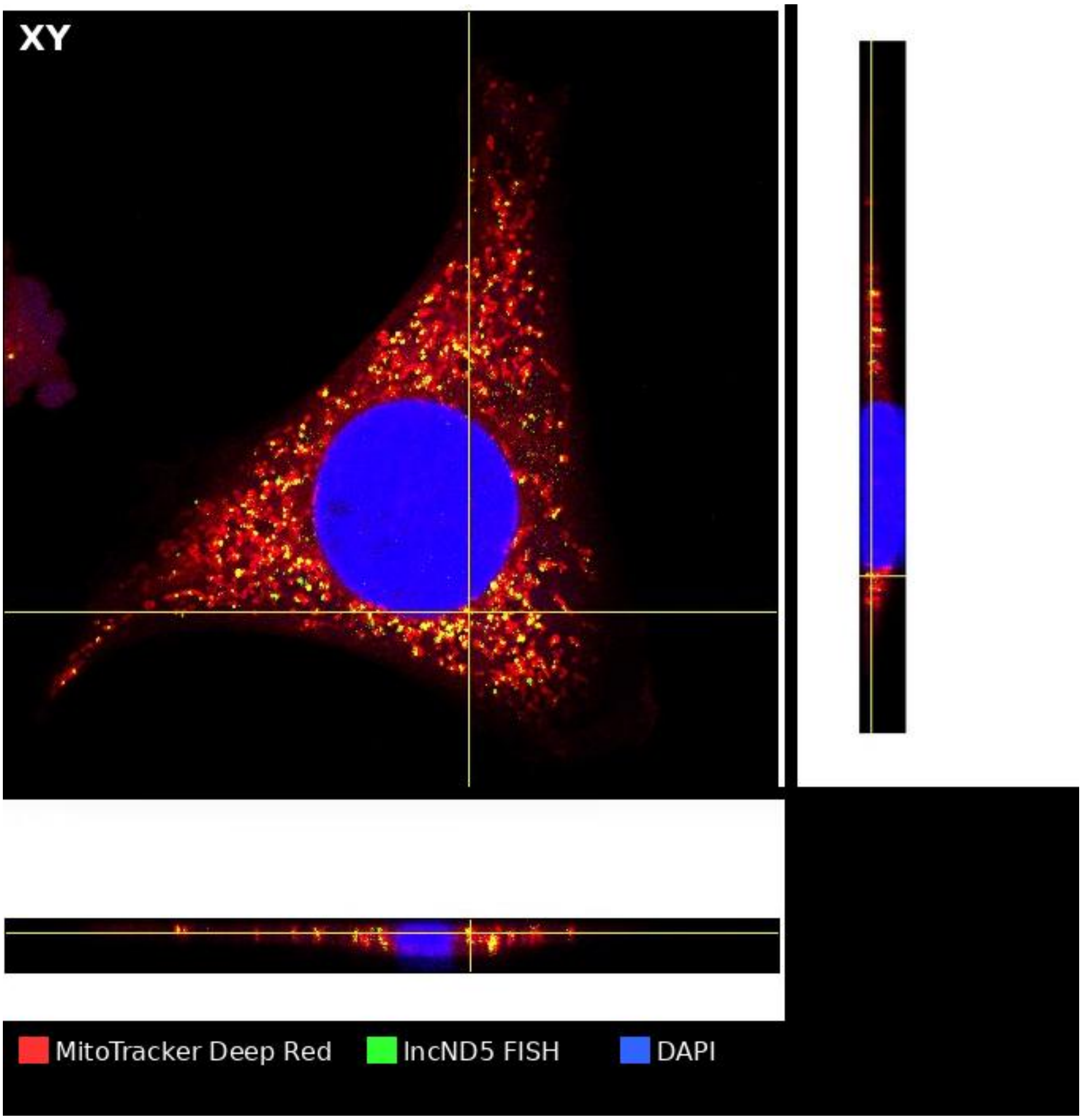

Supplementary Figure S2. Orthogonal views of lncND5 FISH and MitoTracker colocalization. XY (maximum intensity projection), YZ, and XZ orthogonal views of HeLa cells stained with lncND5 FISH probes (Quasar 570, green) and MitoTracker Deep Red (red), with nuclei stained with DAPI (blue). Orthogonal projections confirm true 3D co-occupancy of lncND5 FISH puncta with mitochondrial signal across z-planes, excluding projection artifacts. Images acquired using Zeiss LSM 980 confocal microscope.

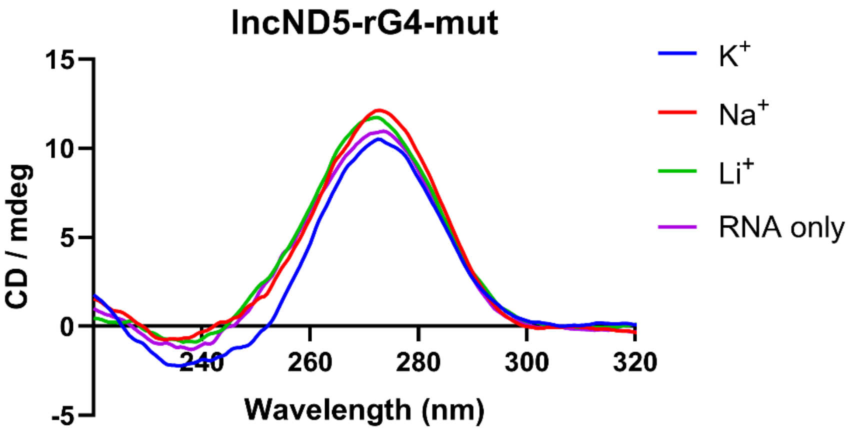

Supplementary Figure S3. The CD spectrum of the lncND5-rG4-mutant (5'-UGAGUAGUGUCAGUUUUUGU-3') sequence showed no characteristic signatures of rG4 formation, lacking the typical absorption maximum at 264 nm and minimum at 240 nm in the rG4-forming environment.

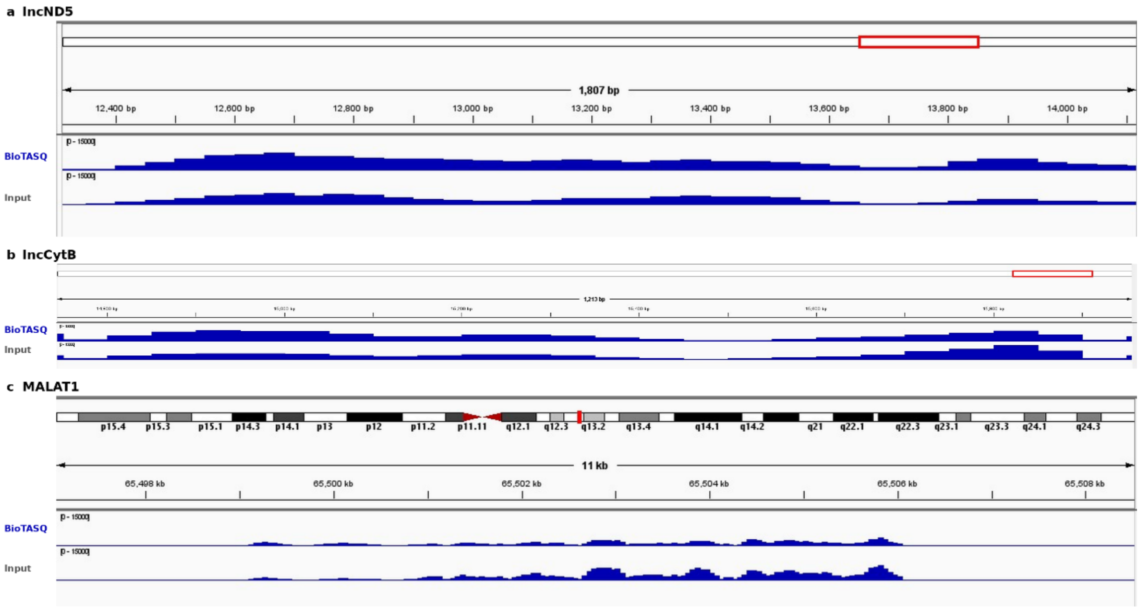

Supplementary Figure S4. G4RP-seq analysis showing rG4 enrichment in mitochondrial lncRNAs. a, IGV browser track at the lncND5 locus (chrM:12,337–14,148) in MCF-7 cells. **b,** IGV browser track at the lncCytB locus (chrM:14,747–15,887). **c,** IGV browser track at the nuclear MALAT1 locus (chr11:65,497,606–65,508,073) shown as a negative control at baseline. In each panel, the upper track represents the untreated BioTASQ-pulldown sample, and the lower track represents the corresponding untreated input control.

A

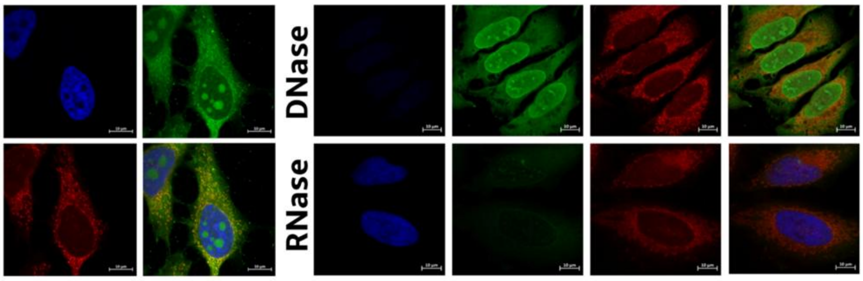

B

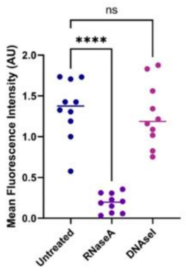

Supplementary Figure S5 A, Bio-orthogonal Imaging of G4 structures inside the HeLa cells treated with BioCyTASQ after fixation, followed by DNase I and RNase A treatment. Nuclei stained with DAPI (blue), AF-SA488 (green), and mitochondria (red). Scale bar, 10µM. **B,** Mean fluorescent intensity between AF-SA488 (green) and mitochondria (red) calculated for each image using ImageJ. The significance is denoted by asterisks using ordinary one-way ANOVA (ns - not significant - $P > 0.05$ and (***) $P < 0.001$

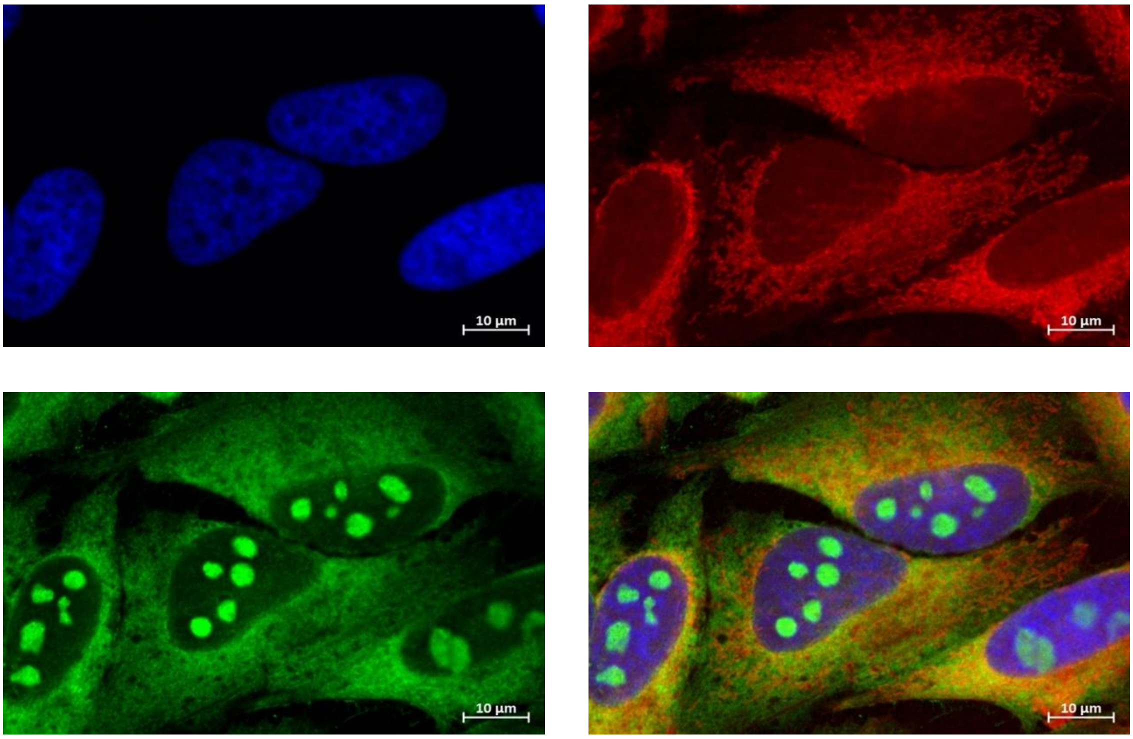

Supplementary Figure S6. Bio-orthogonal Imaging of G4 structures inside the HeLa cells treated with BioCyTASQ after fixation, followed by RNase T1 treatment. Nuclei stained with DAPI (blue), AF-SA488 (green), and mitochondria (red). Scale bar, 10µM.

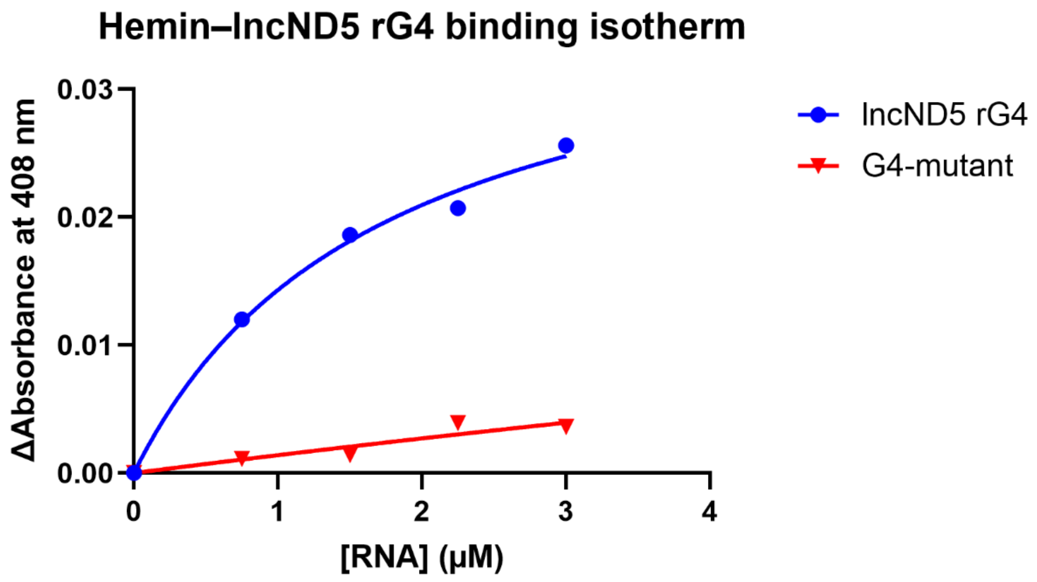

Supplementary Figure S7. Hemin–lncND5 rG4 binding isotherm. ΔAbsorbance at 408 nm (Soret band enhancement) plotted as a function of RNA concentration for lncND5 rG4 (blue circles) and G4-mutant control (red triangles). The lncND5 rG4 data were fitted to a 1:1 specific binding model (solid curve; Kd(app) = 1.73 μM, 95% CI: 0.78–4.29 μM; Bmax = 0.039; R^2 = 0.993). The G4-mutant showed minimal Soret band enhancement across all concentrations tested (ΔA_{408} max = 0.004), approximately 7-fold lower than lncND5 rG4 at equimolar conditions, confirming G4-structure-dependent hemin coordination.

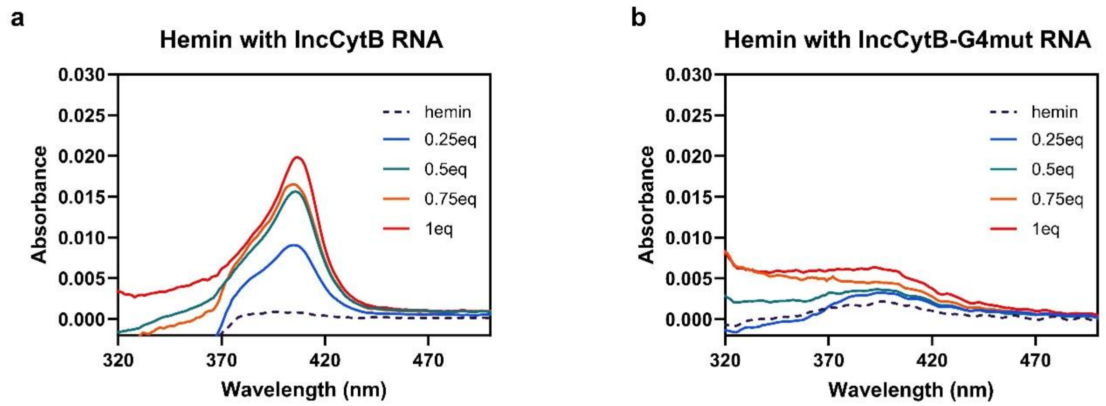

Supplementary Figure S8. Mitochondrial lncCytB G4 structures bind hemin. a, UV-Vis absorbance of hemin titrated with different equivalents of full-length lncCytB showing the Soret band indicating the interaction of G4 with hemin. b, UV-Vis absorbance of hemin with full-length lncCytB G4 mutated sequences

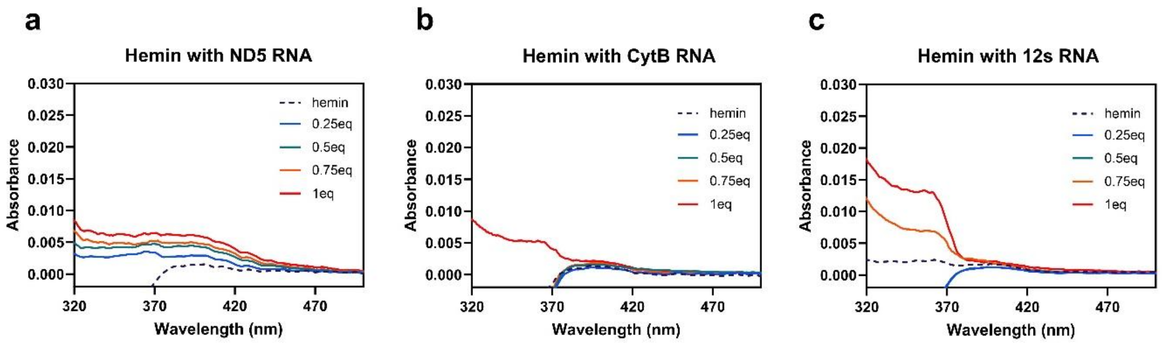

Supplementary Figure S9. Non-G4-forming mitochondrial transcripts lack hemin-binding activity. a,b,c UV-Vis absorbance of hemin titrated with different equivalents of full-length ND5,CytB and 12s RNA showing no significant increase in the Soret band region, indicating the absence of interaction with hemin.

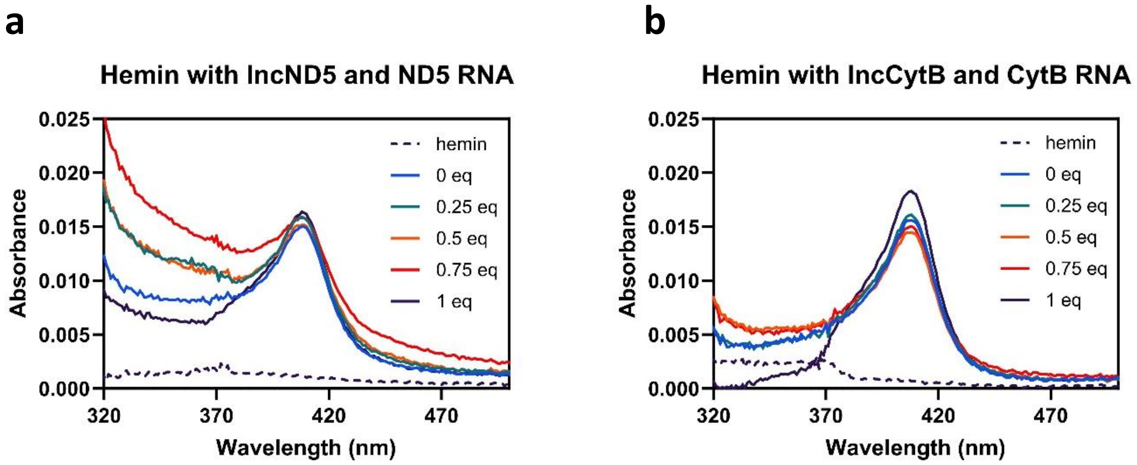

Supplementary Figure S10. Mitochondrial lncND5 and lncCytB G4 structures bind hemin. a, UV-Vis absorbance of hemin together with lncND5 titrated with different equivalents of full-length ND5 showing the Soret band, indicating the interaction of lncND5 with hemin was unaltered in the presence of its complementary strand. b, UV-Vis absorbance of hemin together with lncCytB titrated with different equivalents of full-length CytB showing Soret band indicating the interaction of lncCytB with hemin was unaltered in the presence of its complementary strand.

a

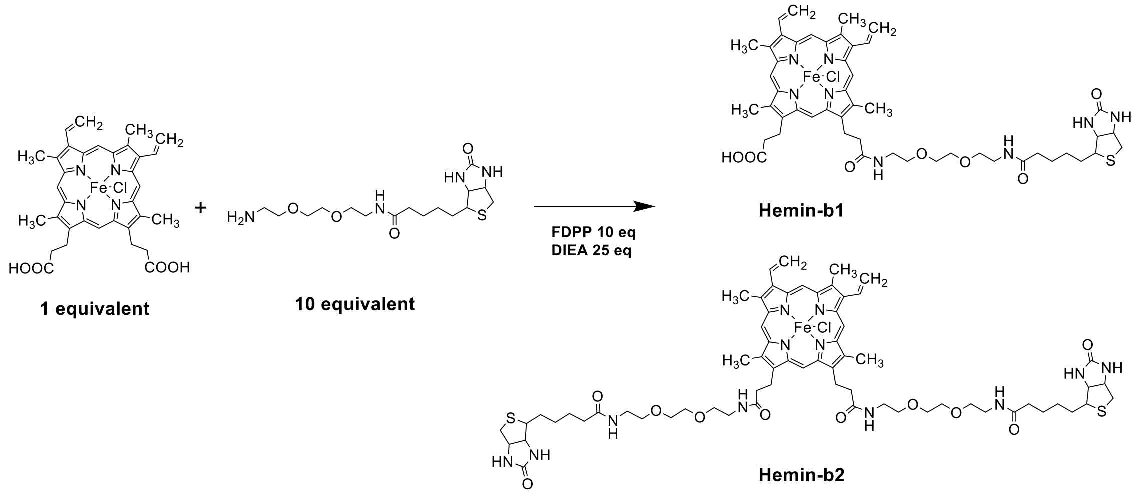

Supplementary Figure S11. Synthesis of biotin-conjugated hemin

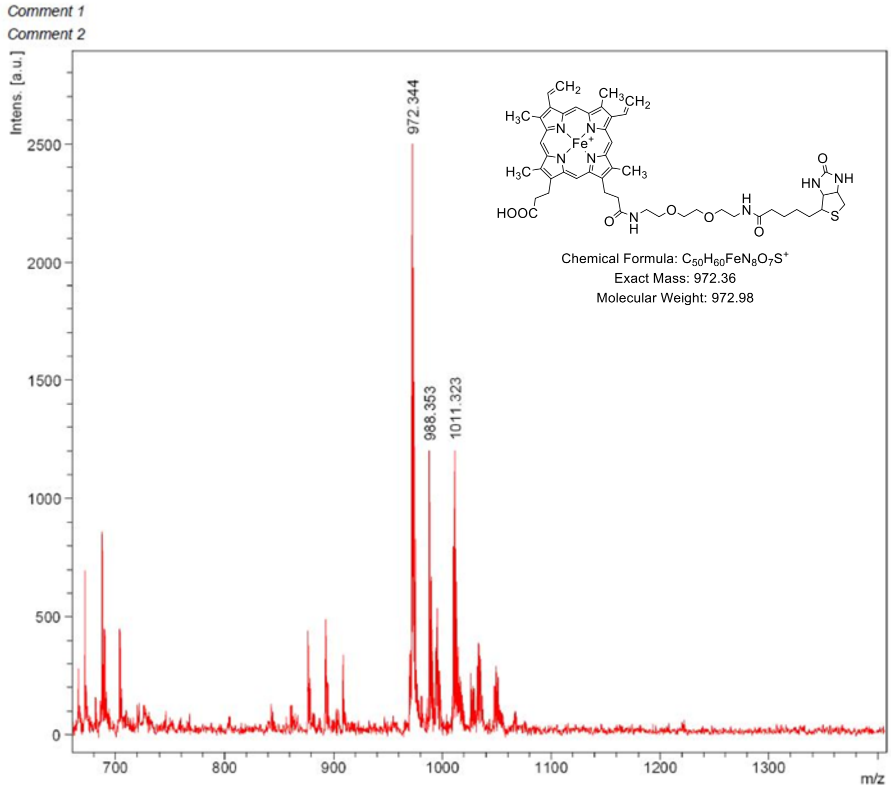

Supplementary Figure S12. MALDI-TOF mass of Hemin-b1

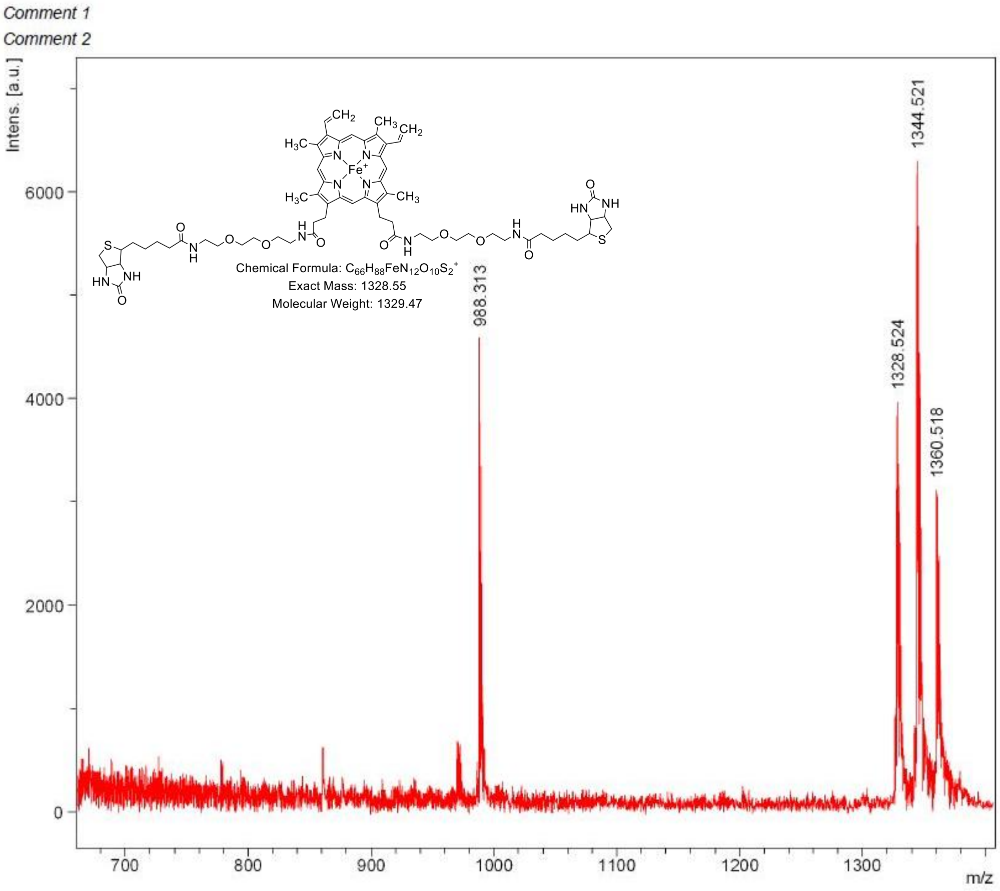

Supplementary Figure S13. MALDI-TOF mass of Hemin-b2

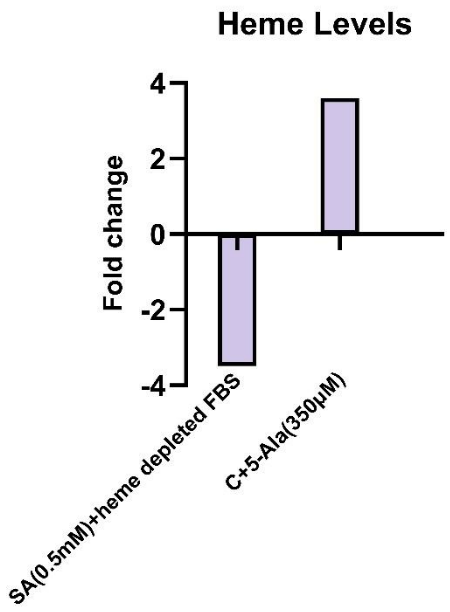

Supplementary Figure S14. Total heme levels in HEK-293 cells upon heme-depletion and 5-Ala treatment

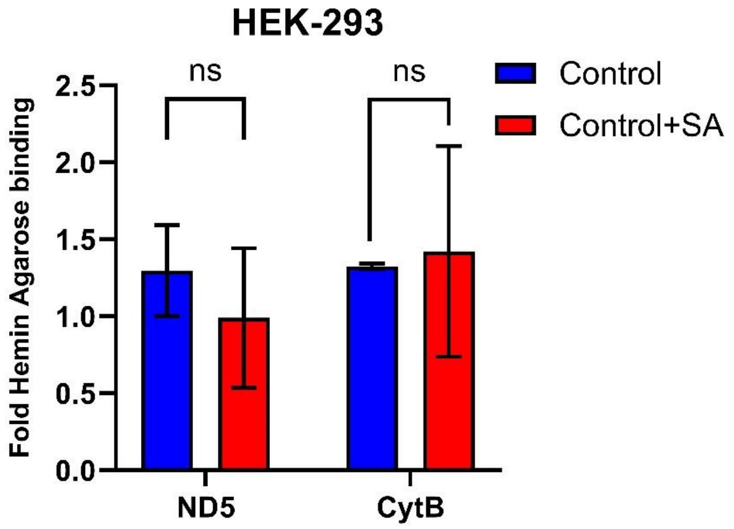

Supplementary Figure S15. ss-RT-qPCR of ND5 and CytB in control and heme-depleted HEK-293 cells after Hemin-Agarose binding, and the fold change is relative to sepharose. (HD+SA denotes Heme-depleted FBS supplemented with 0.5 mM succinylacetone) Data shown as mean ± s.d. (n = 2) and statistical significance are represented by asterisks after performing Student's *t*-test (n.s., not significant)

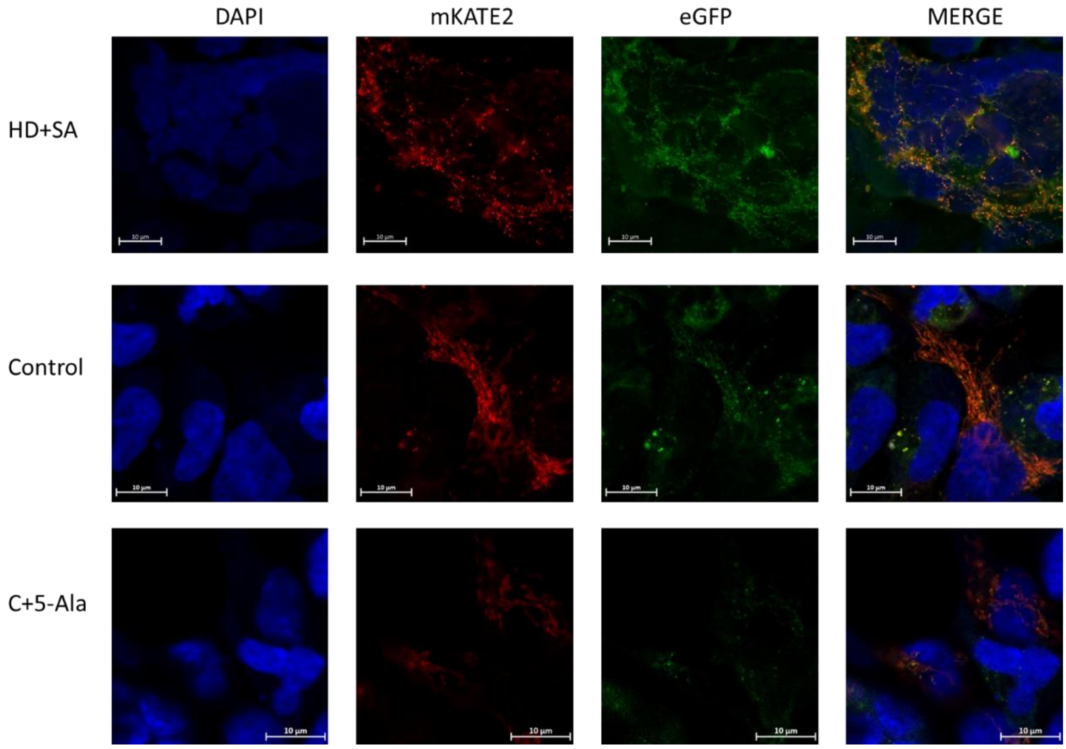

Supplementary Figure S16. Heme sensor targeted towards the mitochondria after transfecting in HEK-293 cells C+5-Ala indicates cells supplemented with 350 µM 5-Aminolevulinic acid and HD+SA denotes Heme-depleted FBS supplemented with 0.5 mM succinylacetone. Scale bar, 10µM.

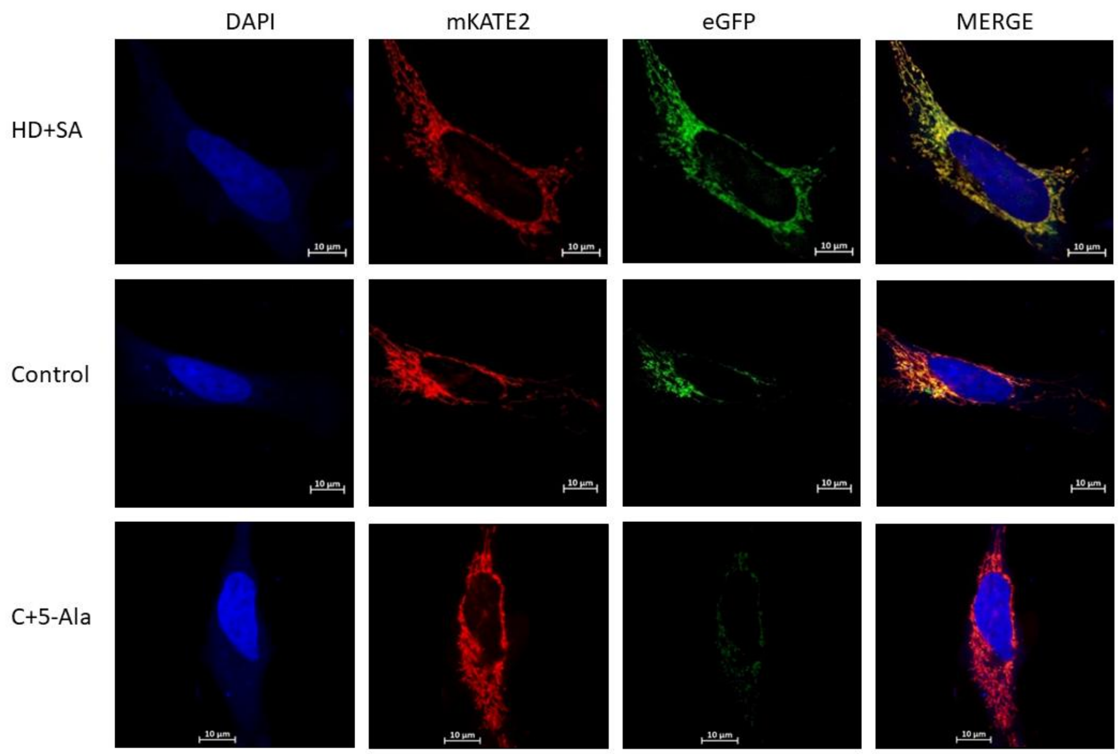

Supplementary Figure S17. Heme sensor targeted towards the mitochondria after transfecting in HeLa cells C+5-Ala indicates cells supplemented with 350 µM 5-Aminolevulinic acid and HD+SA denotes Heme-depleted FBS supplemented with 0.5 mM succinylacetone. Scale bar, 10µM.

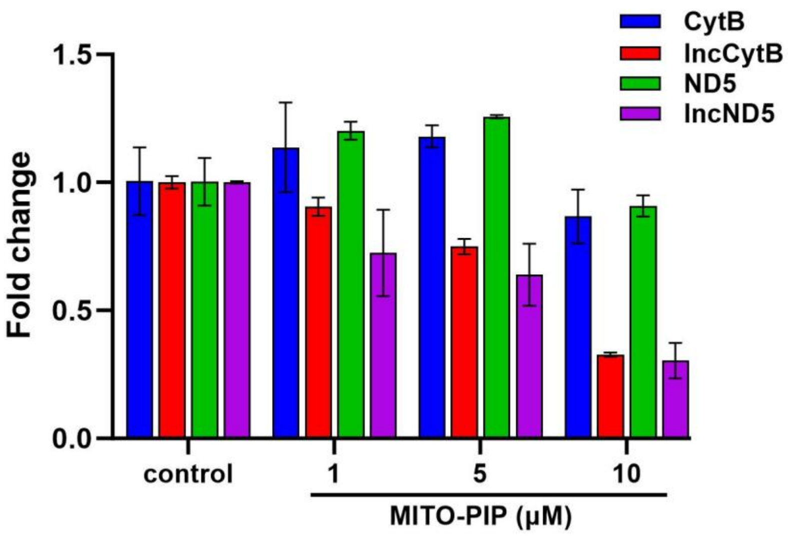

Supplementary Figure S18. ss-RT-qPCR of MITO-PIP treated at various concentration showing the inhibition of lncND5 and lncCytB. Data shown as mean ± s.d.

Gating Strategy

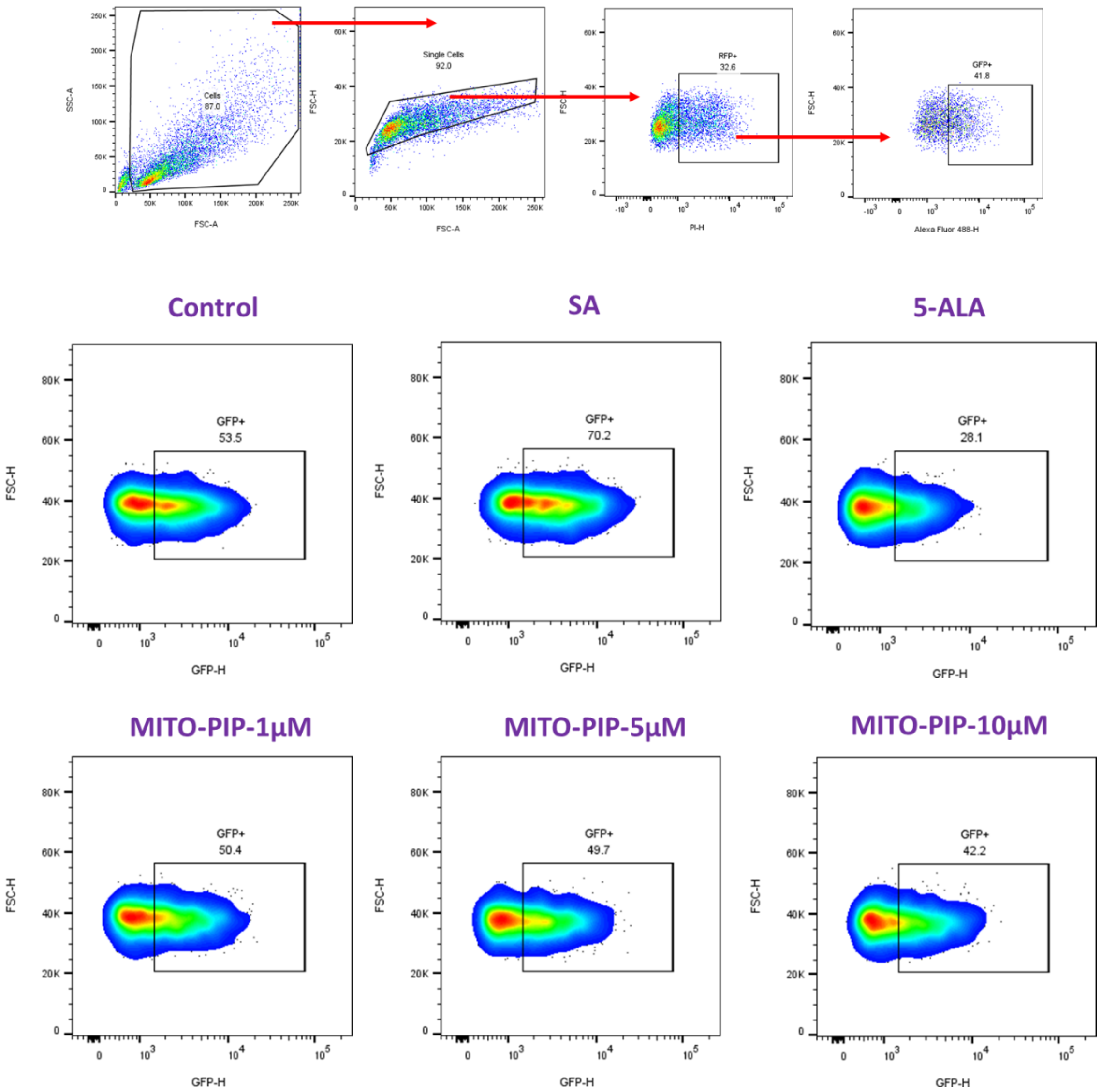

Supplementary Figure S19. Heme sensor targeted towards the mitochondria after transfecting in HEK-293 cells was analysed by flow cytometry. 5-Ala indicates cells supplemented with 350 µM 5-Aminolevulinic acid, SA denotes Heme-depleted FBS supplemented with 0.5mM succinylacetone and MITO-PIP treated at different concentrations for 24 hours

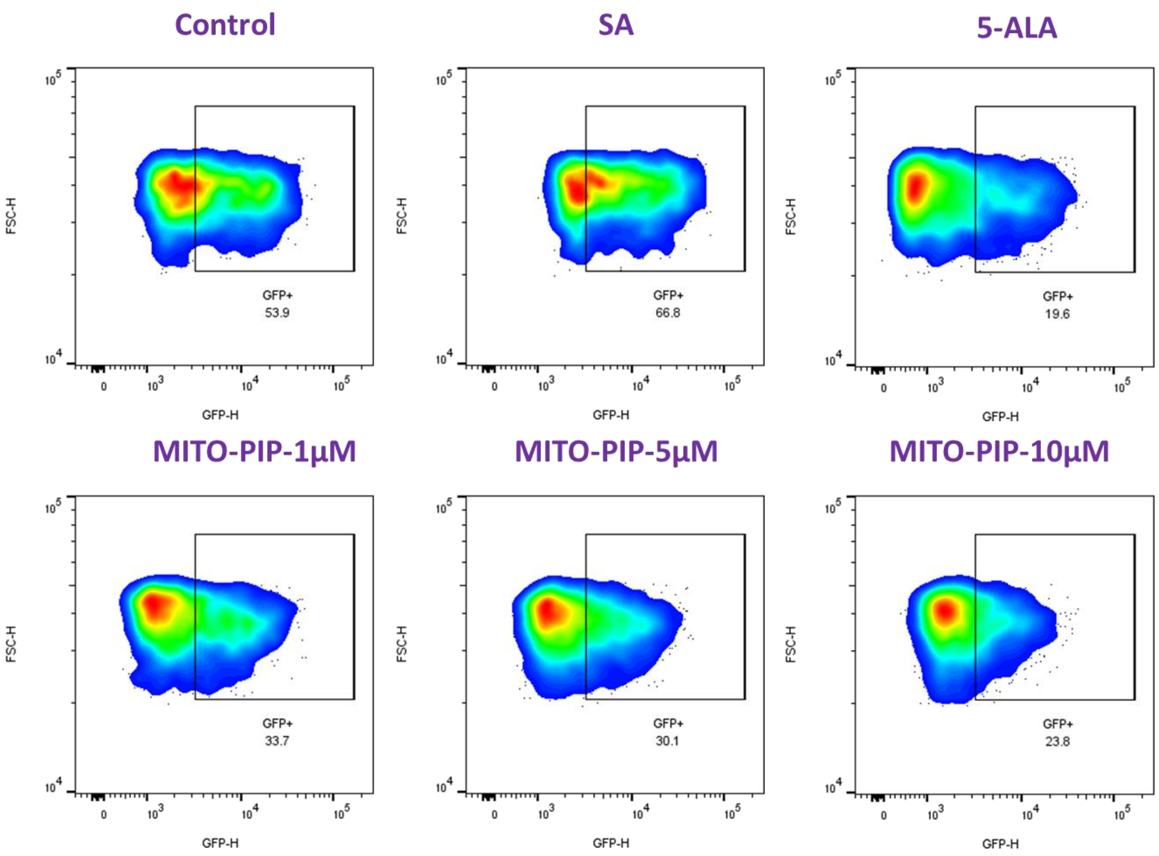

Supplementary Figure S20. Heme sensor targeted towards the mitochondria after transfecting in HeLa cells was analysed by flow cytometry. 5-Ala indicates cells supplemented with 350 µM 5-Aminolevulinic acid, SA denotes Heme-depleted FBS supplemented with 0.5mM succinylacetone and MITO-PIP treated at different concentrations for 24 hours

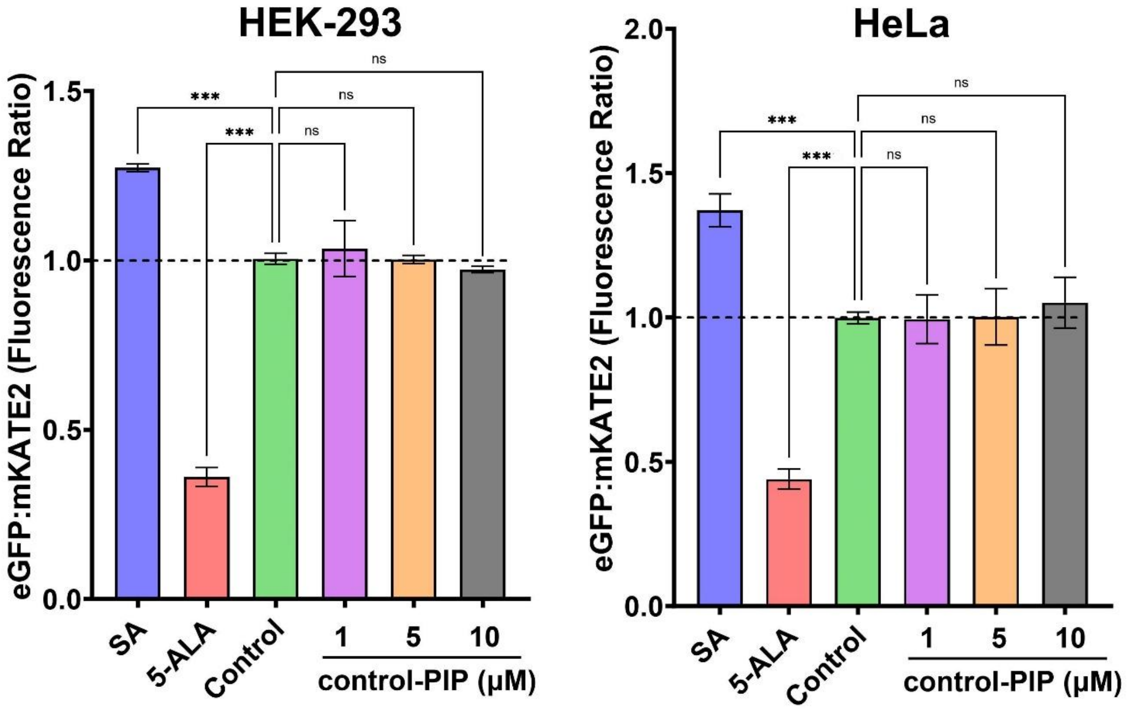

Supplementary Figure S21. Flow cytometry analysis of heme sensor targeted towards mitochondria after transfecting in HEK-293 and HeLa cells. The X-axis indicates the condition (C+5-Ala and control-PIP with indicated concentration) of the cell grown for 24 hours before performing flow cytometry (48 hours in case of HD+SA) The median sensor ratio was obtained by calculating the fluorescence ratio of eGFP to mKATE2. Data shown as mean ± s.d. (n = 2) the statistical significance is represented by asterisks after performing ANOVA (***p<0.001, n.s., not significant - P > 0.05)

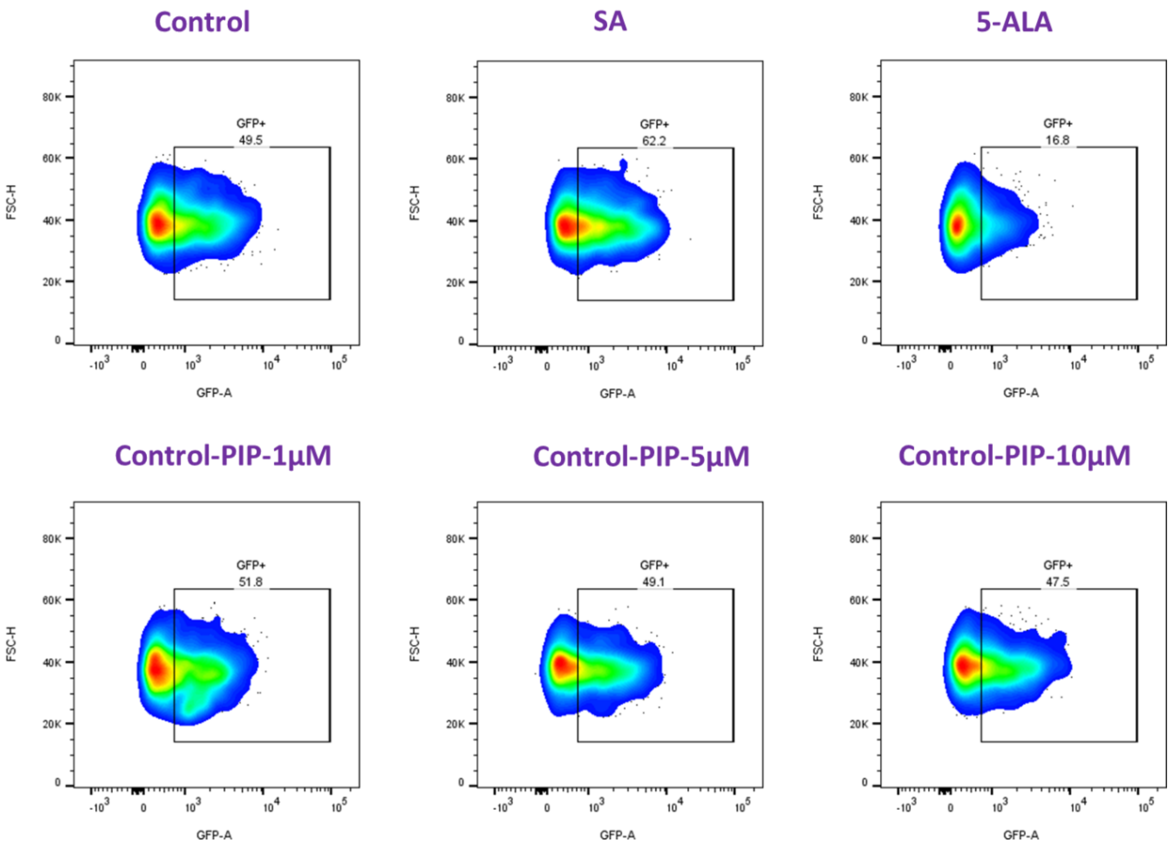

Supplementary Figure S22. Heme sensor targeted towards the mitochondria after transfecting in HEK-293 cells was analysed by flow cytometry. 5-Ala indicates cells supplemented with 350 µM 5-Aminolevulinic acid, SA denotes Heme-depleted FBS supplemented with 0.5mM succinylacetone and Control-PIP treated at different concentrations for 24 hours

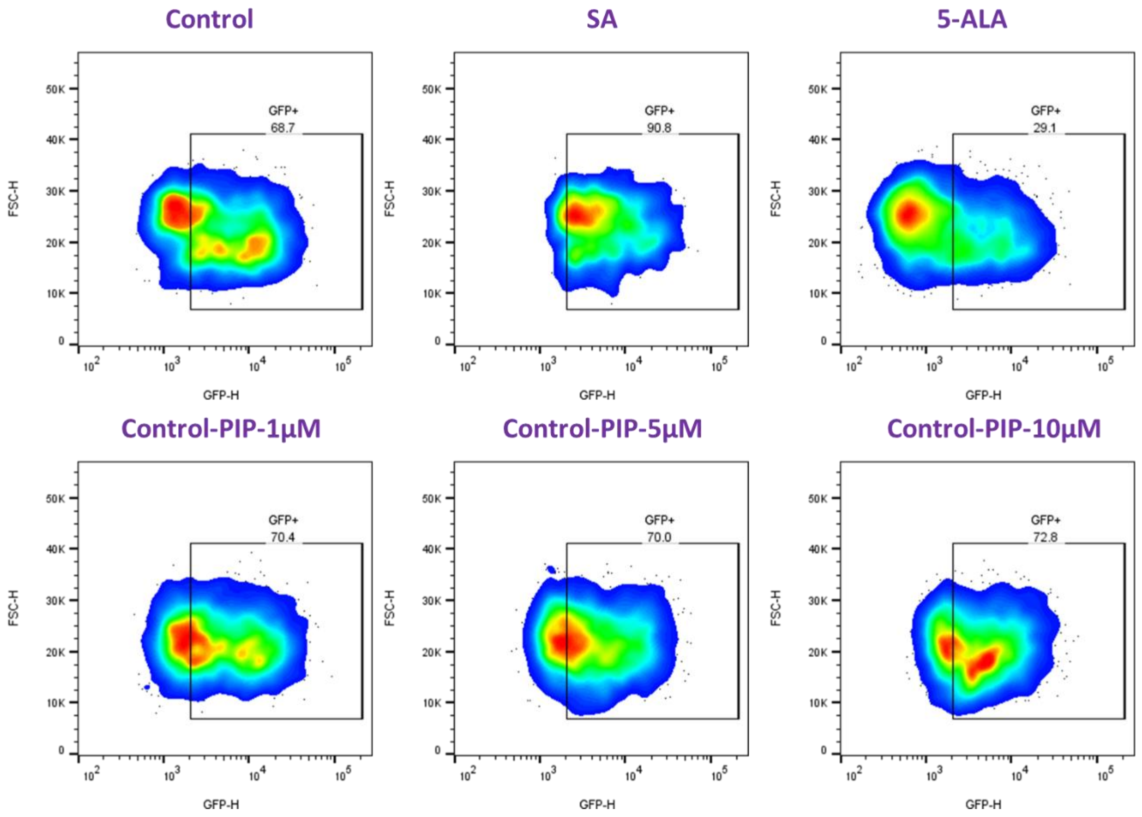

Supplementary Figure S23. Heme sensor targeted towards the mitochondria after transfection in HeLa cells was analysed by flow cytometry. 5-Ala indicates cells supplemented with 350 µM 5-Aminolevulinic acid, SA denotes Heme-depleted FBS supplemented with 0.5mM succinylacetone, and Control-PIP treated at different concentrations for 24 hours

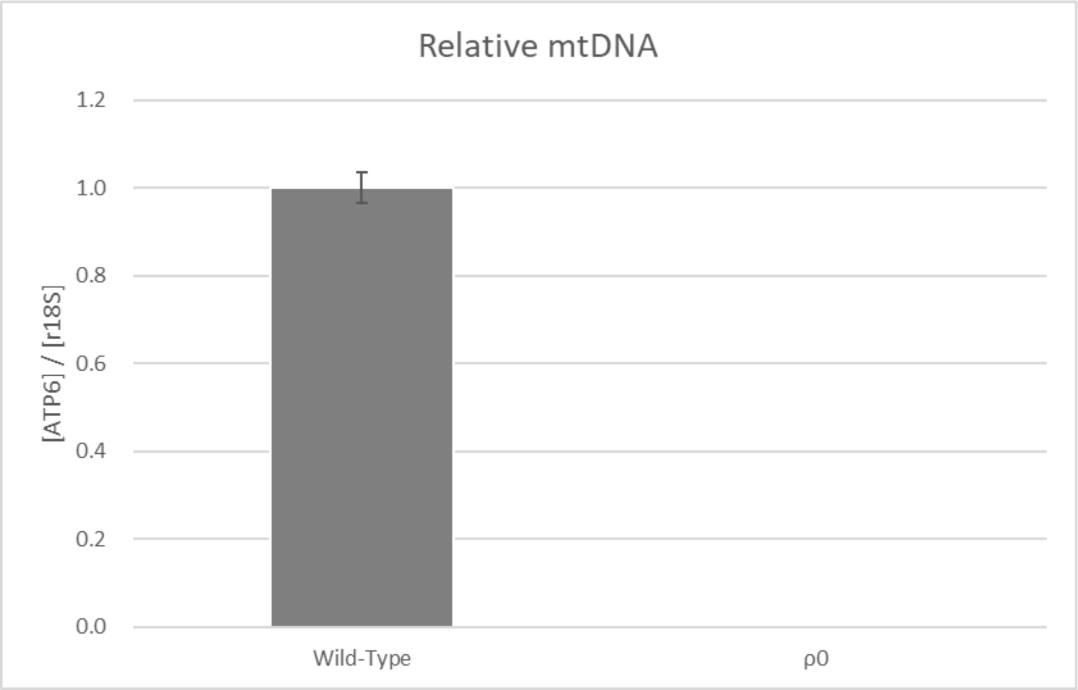

Supplementary Figure S24. qPCR validating the lack of mtDNA in ρ^0 compared to the wild-type HeLa cell line.

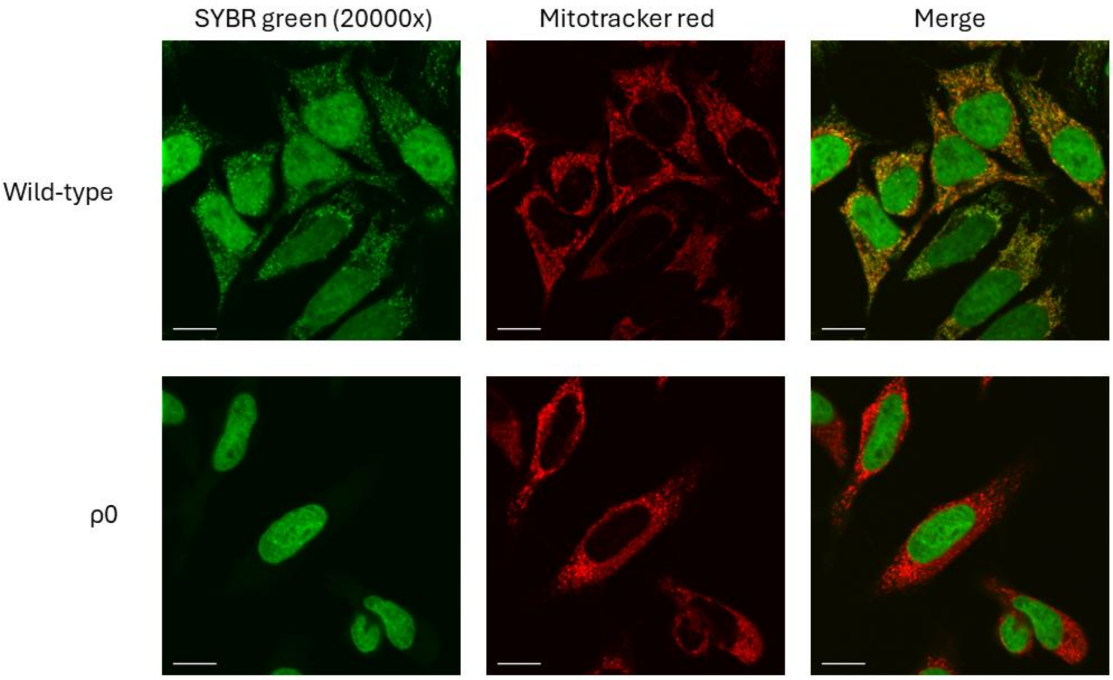

Supplementary Figure S25. Confocal imaging confirming the lack of mtDNA (green) in ρ^0 . Scale bar - 10µM

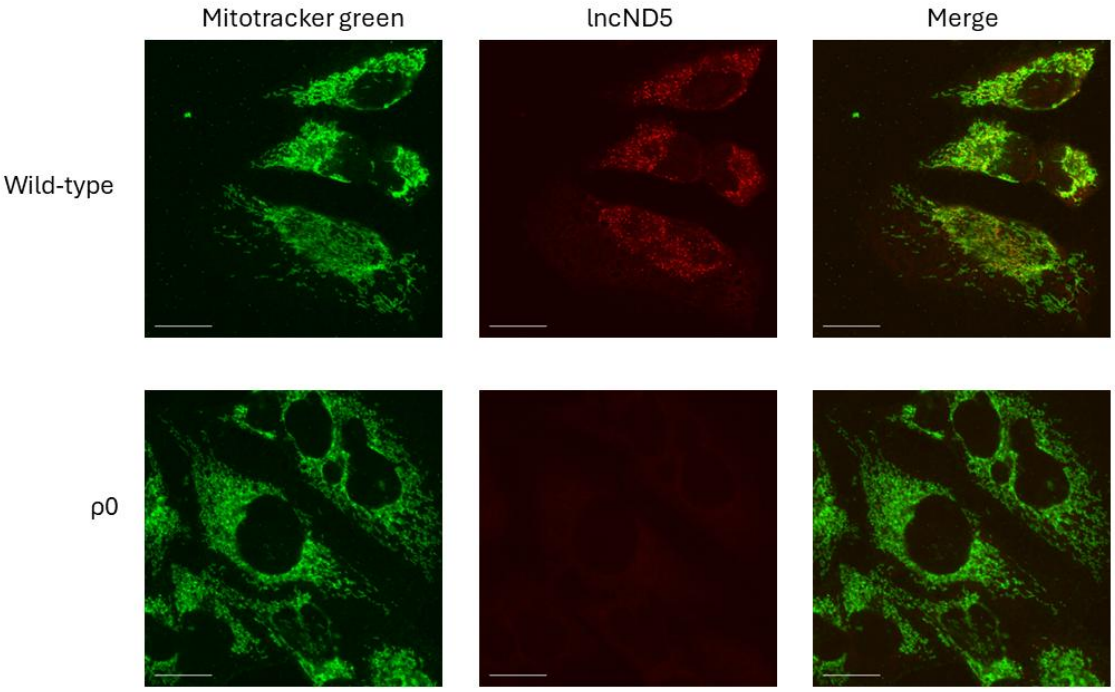

Supplementary Figure S26. Confocal imaging confirming the lack of lncND5 (red) in ρ^0 . Scale bar - 10µM

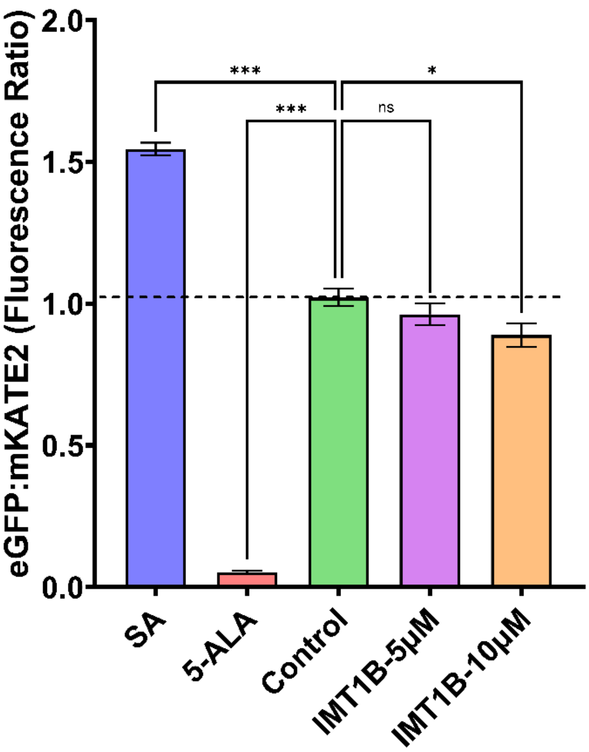

Supplementary Figure S27. Flow cytometry analysis of heme sensor targeted towards mitochondria after transfecting in HEK-293 cells. The X-axis indicates the condition (C+5-Ala and IMT1B with indicated concentration) of the cell grown for 6 hours before performing flow cytometry (24 hours in case of 5-Ala and 48 hours in case of HD+SA) The median sensor ratio was obtained by calculating the fluorescence ratio of eGFP to mKATE2. Data shown as mean ± s.d. (n = 3) the statistical significance is represented by asterisks after performing ANOVA (***p<0.001, n.s., *p < 0.05., not significant - P > 0.05)

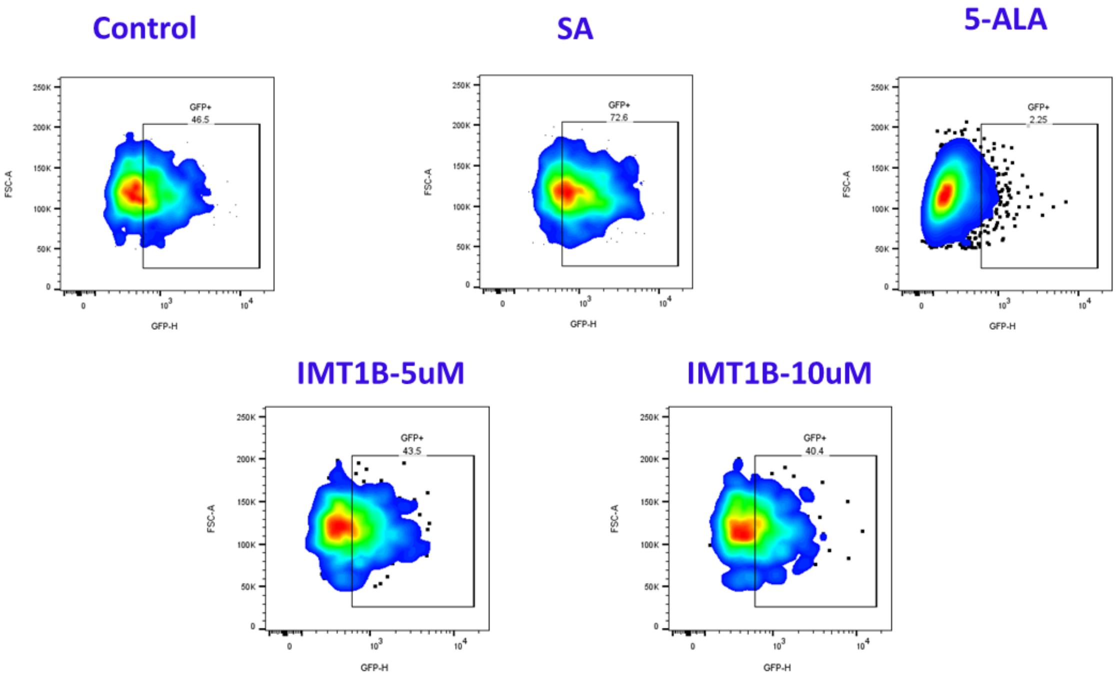

Supplementary Figure S28. Heme sensor targeted towards the mitochondria after transfecting in HEK-293 cells was analysed by flow cytometry. 5-Ala indicates cells supplemented with 350 µM 5-Aminolevulinic acid, SA denotes Heme-depleted FBS supplemented with 0.5mM succinylacetone and IMT1B treated at different concentrations for 6 hours.

a b

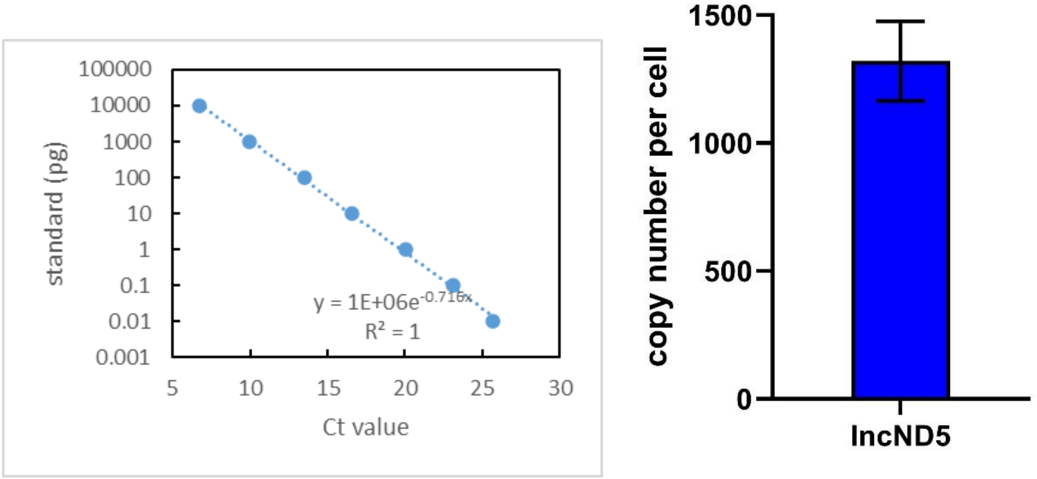

Supplementary Figure S29. **qRT-PCR to determination the copy number of lncND5 RNA** (a) The Ct values of the standard cDNA at known concentration (b) copy number of lncND5 determined using the standard curve. The total RNA levels was used as reported by Han et.al [2]

Assuming that lncND5 is the primary heme buffer in mitochondria, free heme is governed by the following equilibria:

[lncND5-heme] ←→ [lncND5] + [heme]

Thus, the concentration of free heme is governed by the total concentration of heme, total concentration of lncND5 rG4s, and the lncND5-heme dissociation constant. Although heme can be found in two oxidation states, a reduced ferrous state and an oxidized ferric state, a prior study of HEK293 cells found that free heme is largely oxidized [3]. Thus, we assume heme is largely oxidized. The total concentration of mitochondrial heme was previously found to be on the order of ~100 uM [4,5]. The concentration of mitochondrial lncND5 is estimated to span between 20 and 40 uM given that its cellular copy number is estimated to be 1000 using qPCR and assuming it is primarily found in the mitochondria, which have volumes estimated to range between .04 to .08 fL [6],. Since lncND5 can potentially form up to 20 G4s (Fig.S2), the concentration of lncND5-derived G4s spans 20 to 800 uM. This model assumes the heme dissociation constant of lncND5 rG4 to be similar to other rG4s, ~10 nM [7]. Using ChemEQL v3.2 [8] https://www.eawag.ch/en/department/surf/projects/chemeql/, the following speciation plot was generated using the aforementioned assumptions.

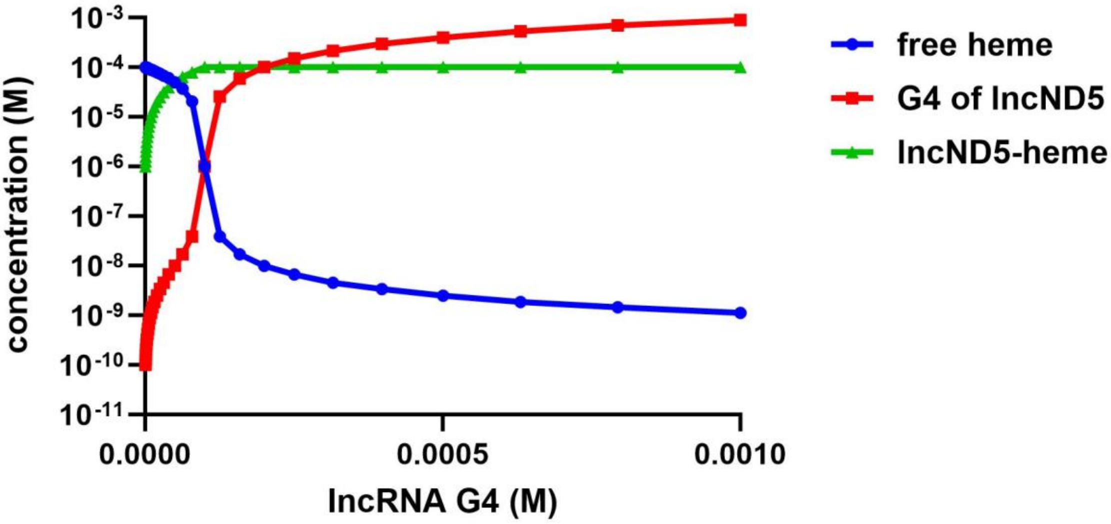

Supplementary Figure S30. **The relationship between concentration of free heme (Y-axis) and concentration of lncRNA G4 (X-axis)** simulated as a function using various concentration using ChemEQL (v. 3.2.1) described above.

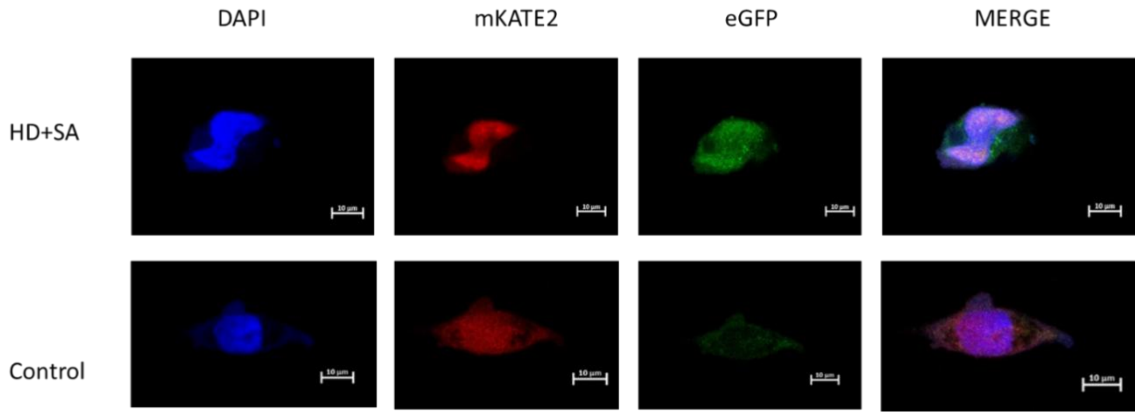

Supplementary Figure S31: Heme sensor targeted towards the nucleus after transfecting in HEK-293 cells HD+SA denotes Heme-depleted FBS supplemented with 0.5 mM succinylacetone. Scale bar, 10µM.

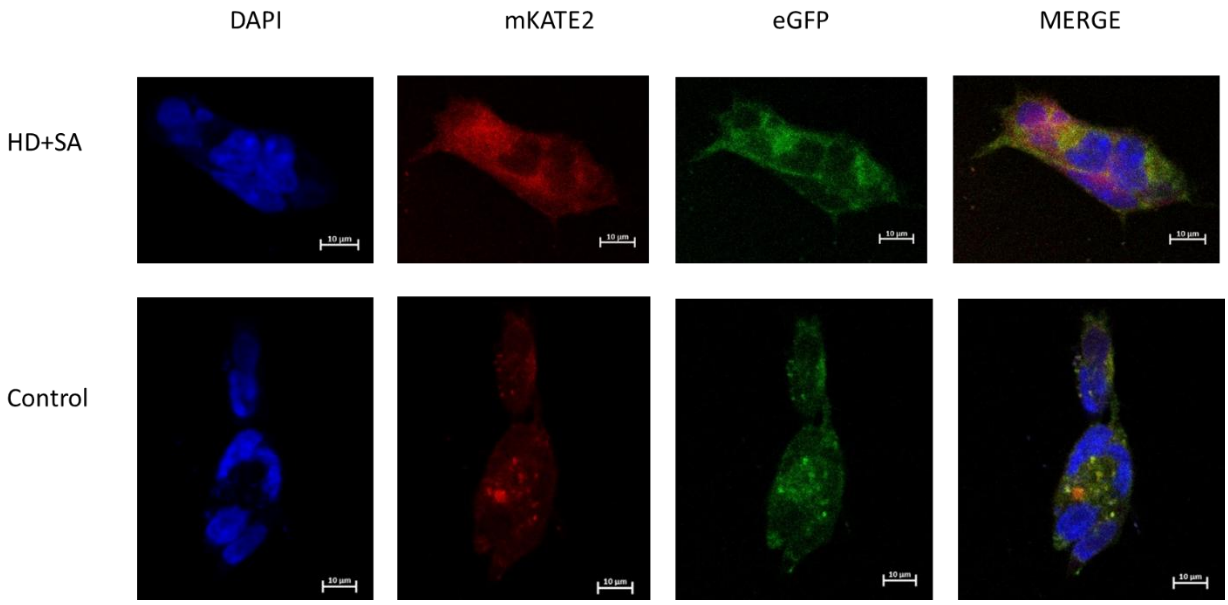

Supplementary Figure S32. Heme sensor targeted towards the cytoplasm after transfecting in HEK-293 cells HD+SA denotes Heme-depleted FBS supplemented with 0.5 mM succinylacetone. . Scale bar, 10μM.

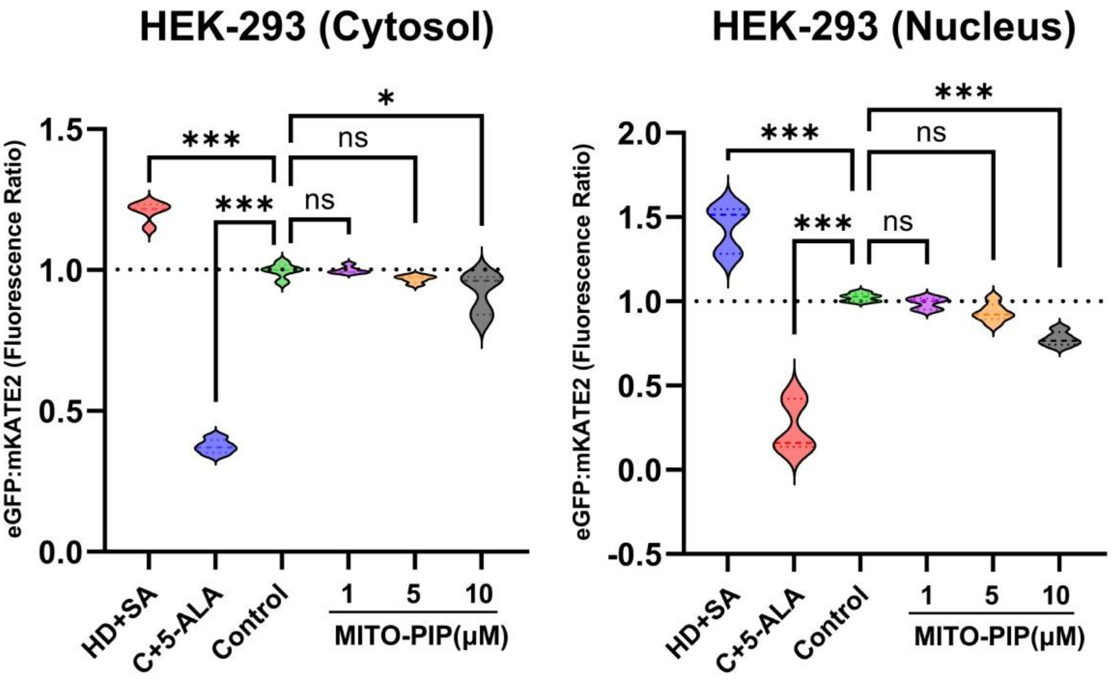

Supplementary Figure S33. Flow cytometry analysis of heme sensor targeted towards cytosol and nuclear compartment after transfection in HEK-293 cells. Data shown as mean ± s.d. (n = 2). The statistical significance is represented by asterisks after performing ANOVA (***p<0.001, **p<0.01, *p < 0.05, n.s., not significant - P > 0.05)

(a)

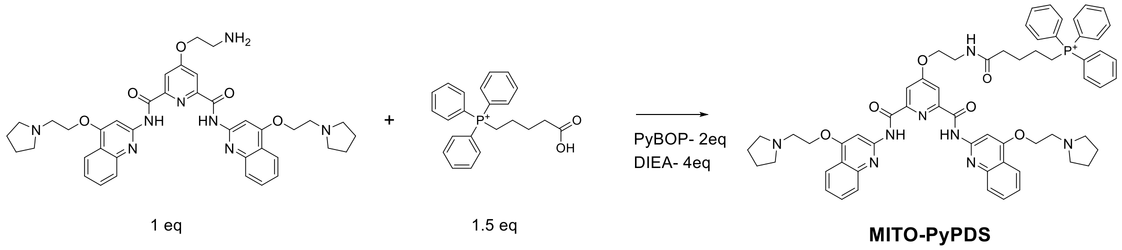

(b)

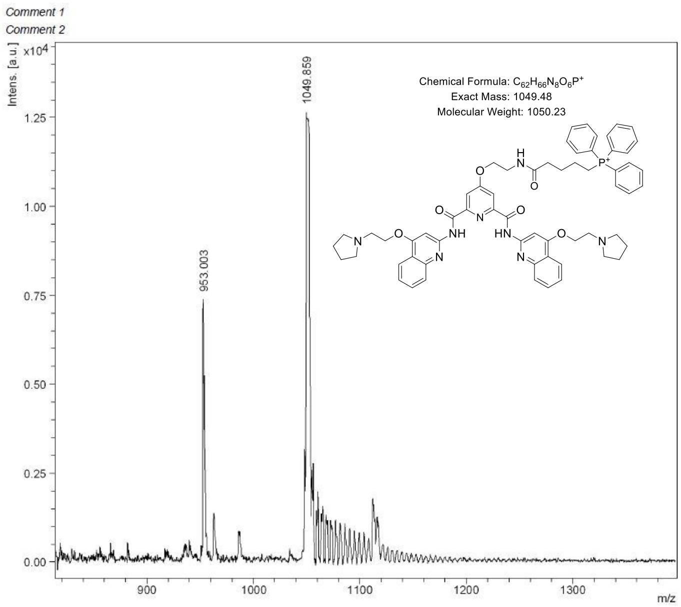

(c)

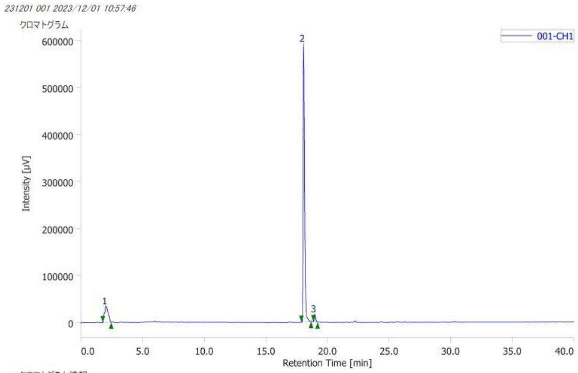

クロマトグラム情報
更新日時 2023/12/01 10:57:39
コメント HPLC-B, ChemcoPaK CHEMCOBOND 5-ODS-H, 40°C, 0–100%, 40min, MeCN/0.1% TFA water, 1.0 mL/min
注入量 2.5 [µL]
サンプル名
取込時間 40.0 [min]
追加情報

ピーク情報

#	tR [min]	面積 [µV·sec]	高さ [µV]	面積%	高さ%	分離度	警告
1	2.033	721265	34607	12.040	5.382	37.482	
2	18.067	5143048	591905	85.850	92.057	4.270	
3	18.983	126390	16467	2.110	2.561	N/A	

Supplementary Figure S34. (a) Synthesis of MITO-PyPDS (b) MALDI-TOF mass of MITO-PyPDS (c) HPLC peak showing the peak of MITO-PyPDS

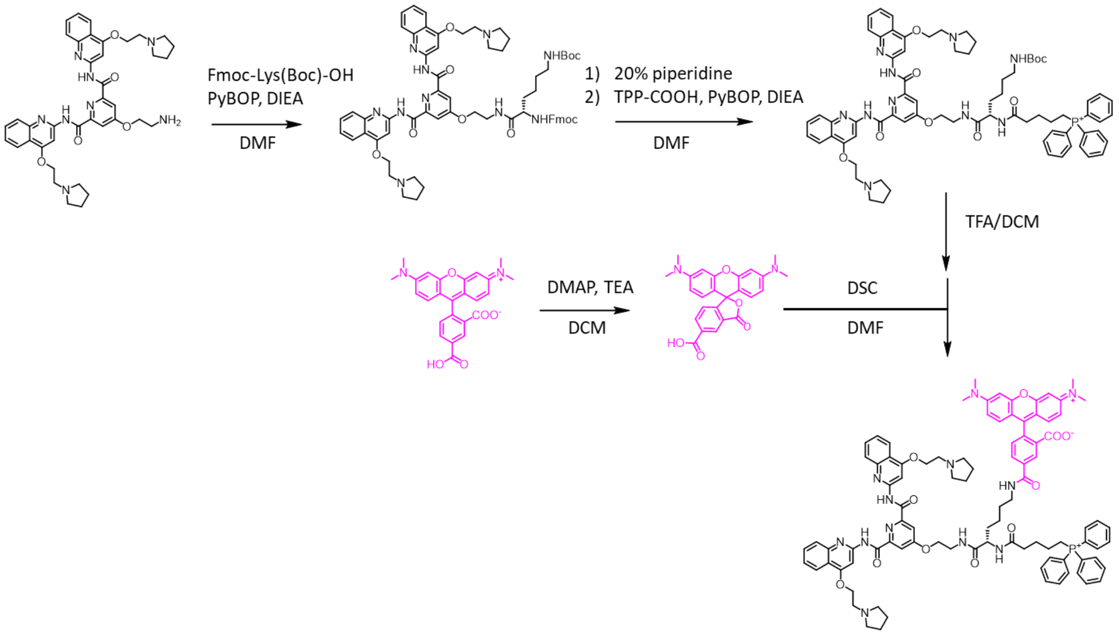

Supplementary Figure S35. **Synthesis of TAMRA-conjugated MITO-PyPDS**

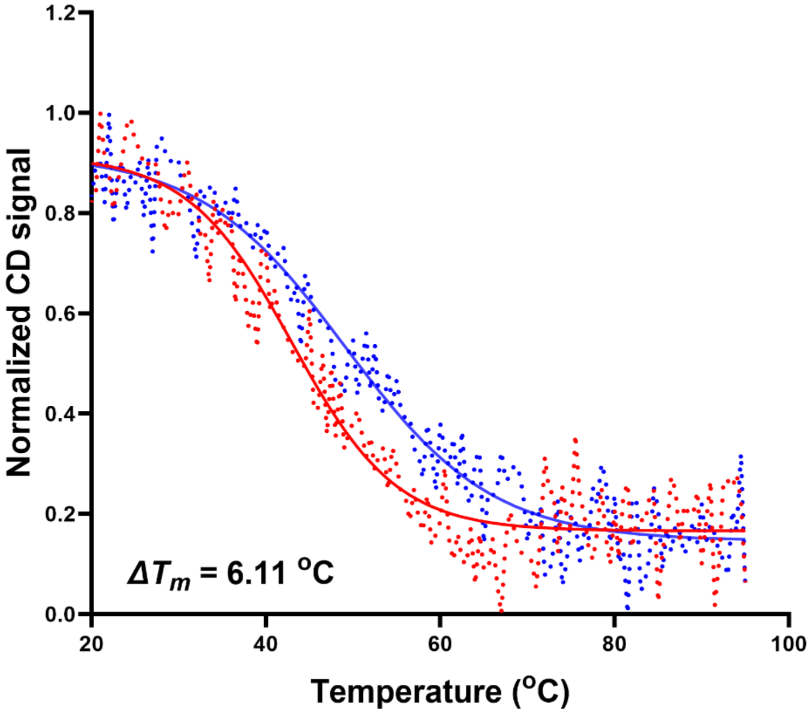

Supplementary Figure S36. Normalized CD melting curves of the RNA sequence (5 μM) in the absence (red) or presence of 3 molar equivalent of MITO-PyPDS (blue)

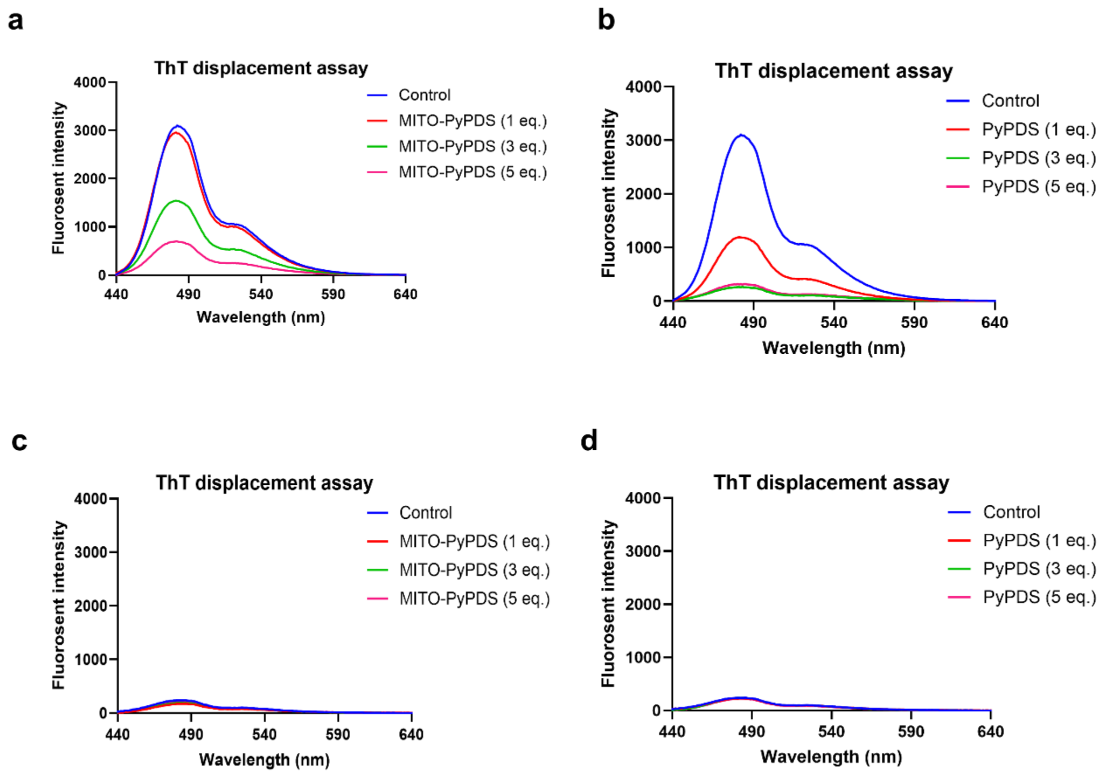

Supplementary Figure S37. Fluorescence intensity of rG4/rG4-mutant sequences with Thioflavin T (ThT) and MITO-PyPDS and PyPDS. a and b The intensity of MITO-PyPDS and PyPDS addition at different equivalents reduced, indicating the displacement of ThT in the presence of the rG4 sequence. c and d The intensity of MITO-PyPDS and PyPDS addition at different equivalents in the rG4 mutant sequence showed no change in the presence of ThT, indicating that the signal of ThT and displacement is mediated by rG4 sequences.

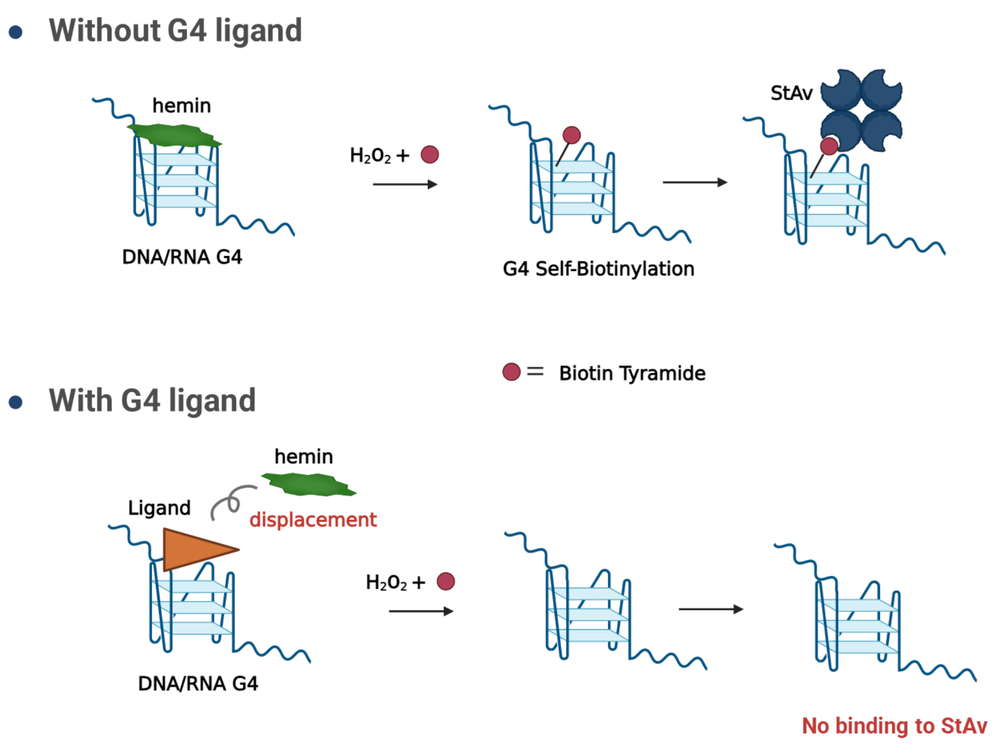

Supplementary Figure S38. Schematic of biotinylation competition experiment.

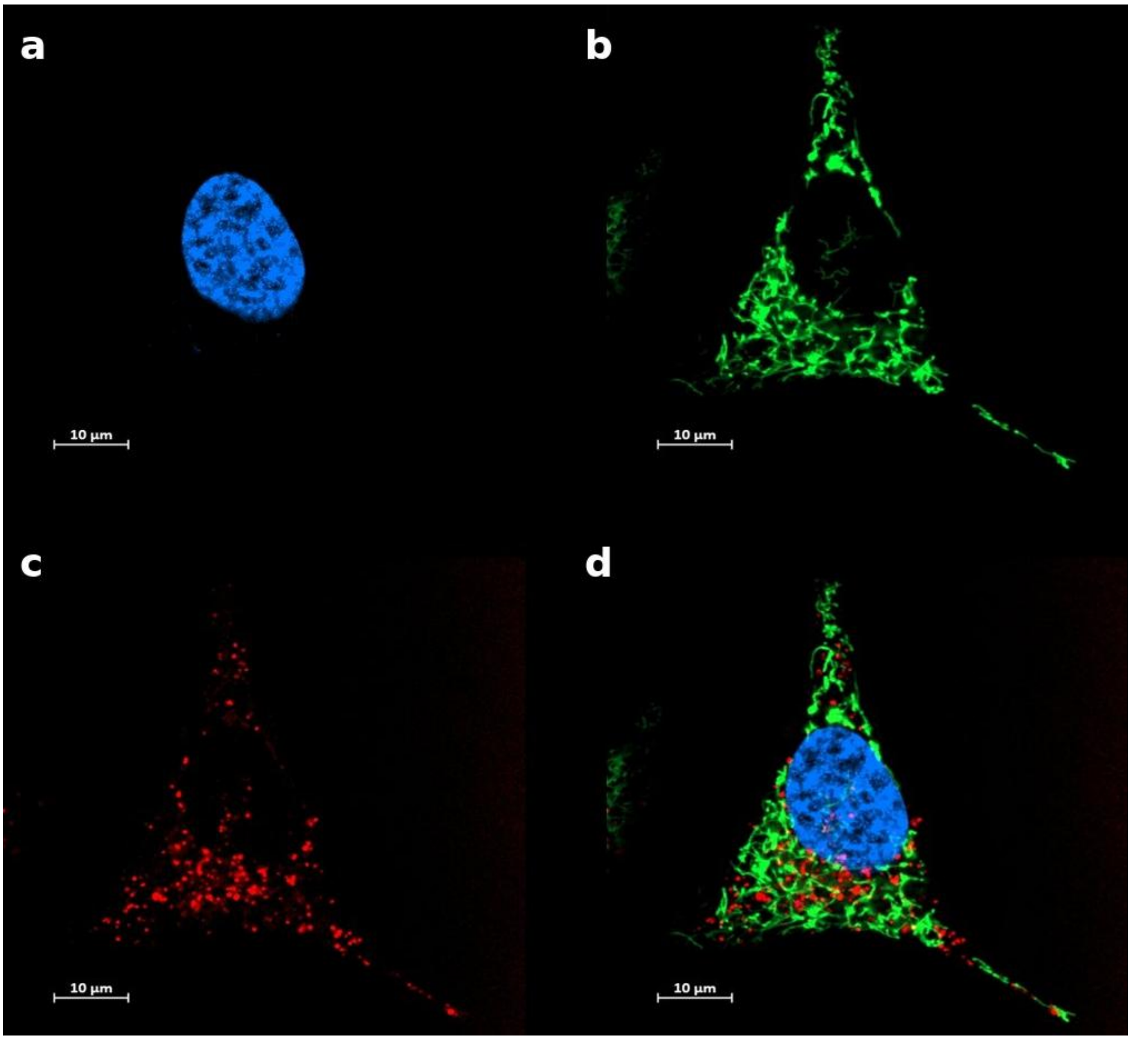

Supplementary Figure S39. Subcellular localization of TAMRA-MITO-PyPDS in live HeLa cells. Confocal microscopy images of HeLa cells treated with 1 µM TAMRA-MITO-PyPDS for 6 hours. **a,** Hoechst-33342 (blue, nucleus). **b,** MitoTracker Deep Red (green, mitochondria). **c,** TAMRA-MITO-PyPDS (red). **d,** Merged image. TAMRA-MITO-PyPDS shows preferential accumulation in the mitochondrial network with clear nuclear exclusion. Scale bar: 10 µm.

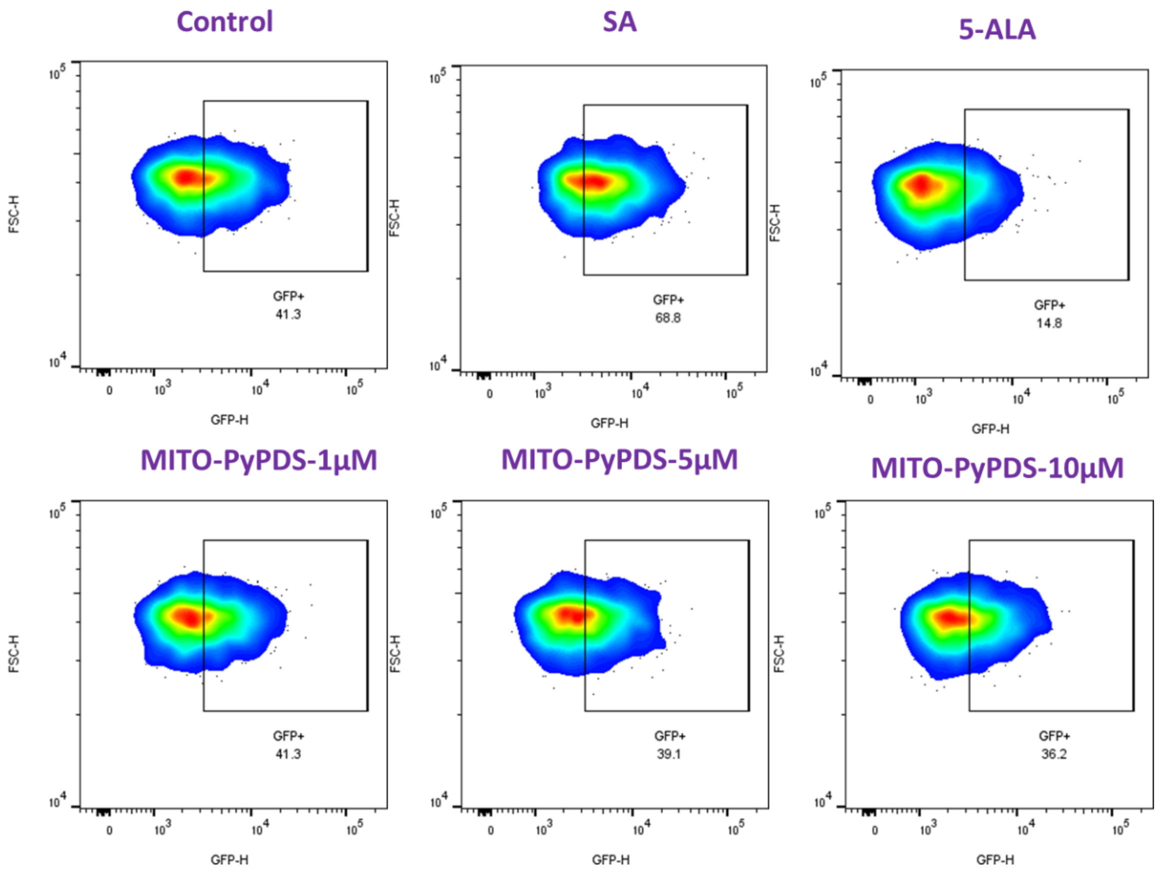

Supplementary Figure S40. Heme sensor targeted towards the mitochondria after transfecting in HEK-293 cells was analysed by flow cytometry. 5-Ala indicates cells supplemented with 350 μM 5-Aminolevulinic acid, SA denotes Heme-depleted FBS supplemented with 0.5mM succinylacetone and MITO-PyPDS treated at different concentrations for 24 hours

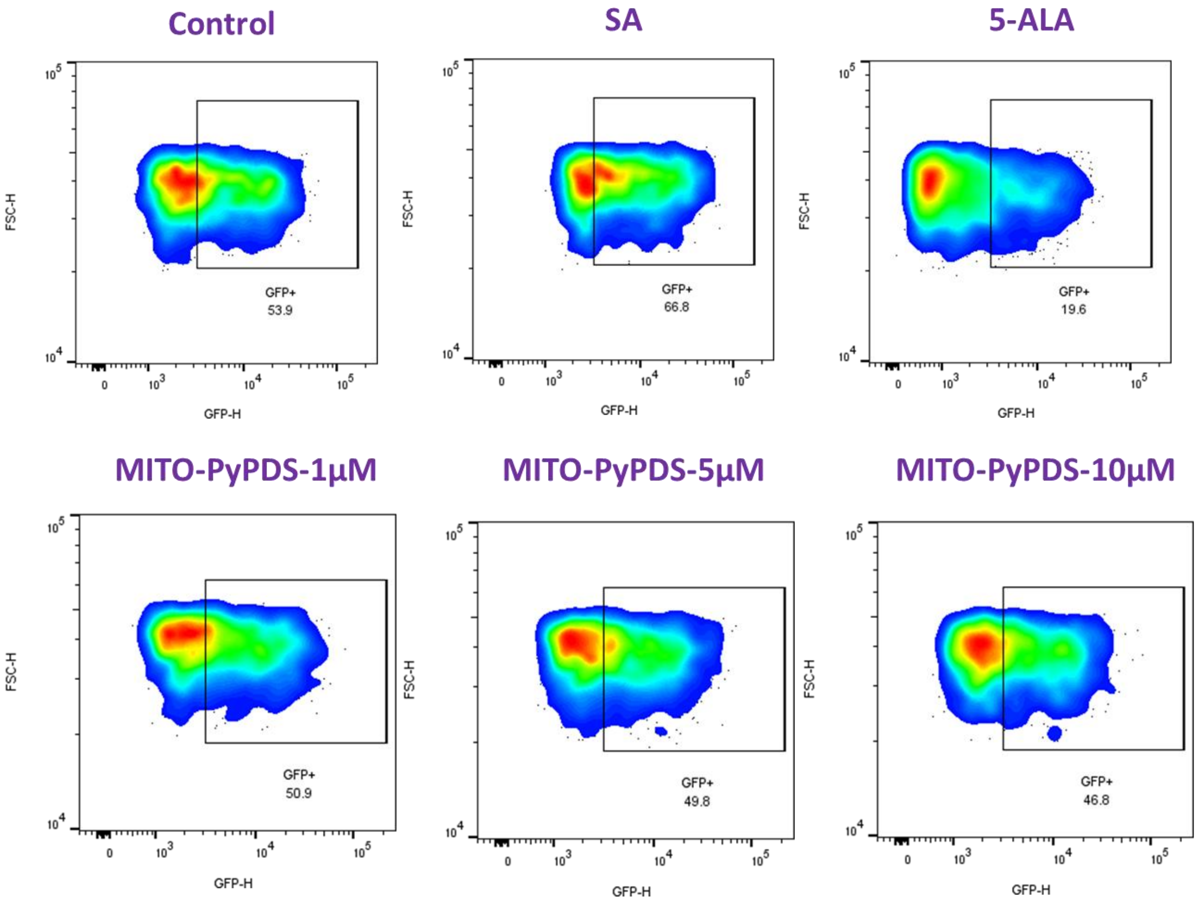

Supplementary Figure S41. Heme sensor targeted towards the mitochondria after transfecting in HeLa cells was analysed by flow cytometry. 5-Ala indicates cells supplemented with 350 µM 5-Aminolevulinic acid, SA denotes Heme-depleted FBS supplemented with 0.5mM succinylacetone and MITO-PyPDS treated at different concentrations for 24 hours

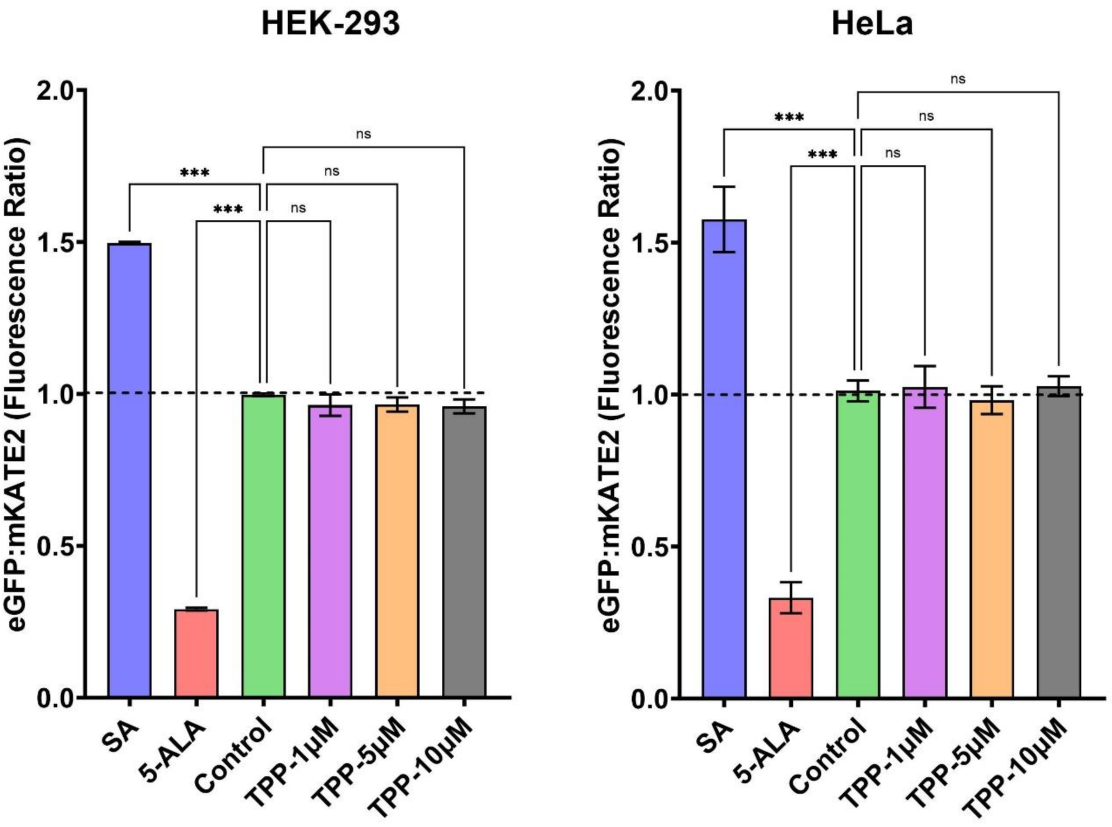

Supplementary Figure S42. Flow cytometry analysis of heme sensor targeted towards mitochondria after transfecting in HEK-293 and HeLa cells. The X-axis indicates the condition (C+5-Ala and TPP with indicated concentration) of the cell grown for 24 hours before performing flow cytometry (48 hours in case of HD+SA) The median sensor ratio was obtained by calculating the fluorescence ratio of eGFP to mKATE2. Data shown as mean ± s.d. (n = 2) the statistical significance is represented by asterisks after performing ANOVA (***p<0.001, n.s., not significant - P > 0.05)

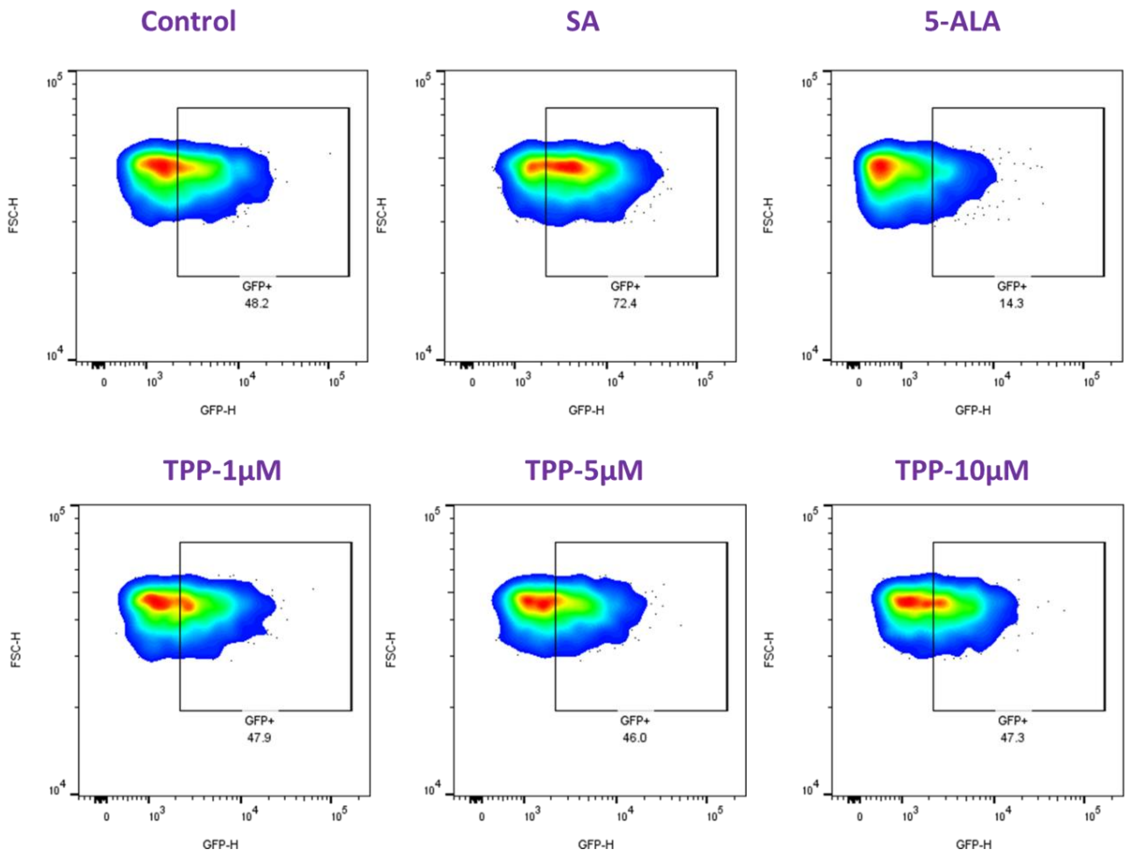

Supplementary Figure S43. Heme sensor targeted towards the mitochondria after transfecting in HEK-293 cells was analysed by flow cytometry. 5-Ala indicates cells supplemented with 350 µM 5-Aminolevulinic acid, SA denotes Heme-depleted FBS supplemented with 0.5mM succinylacetone and TPP treated at different concentrations for 24 hours

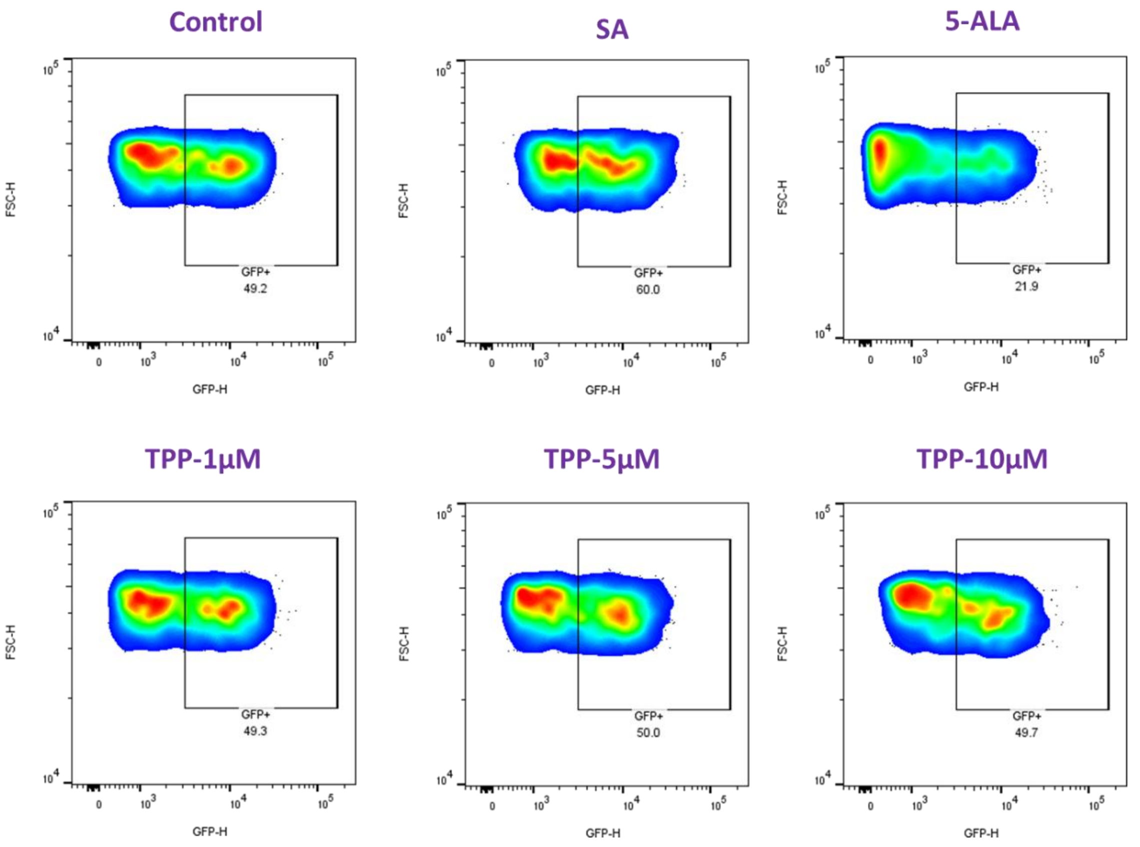

Supplementary Figure S44. Heme sensor targeted towards the mitochondria after transfecting in HeLa cells was analysed by flow cytometry. 5-Ala indicates cells supplemented with 350 µM 5-Aminolevulinic acid, SA denotes Heme-depleted FBS supplemented with 0.5mM succinylacetone and TPP treated at different concentrations for 24 hours

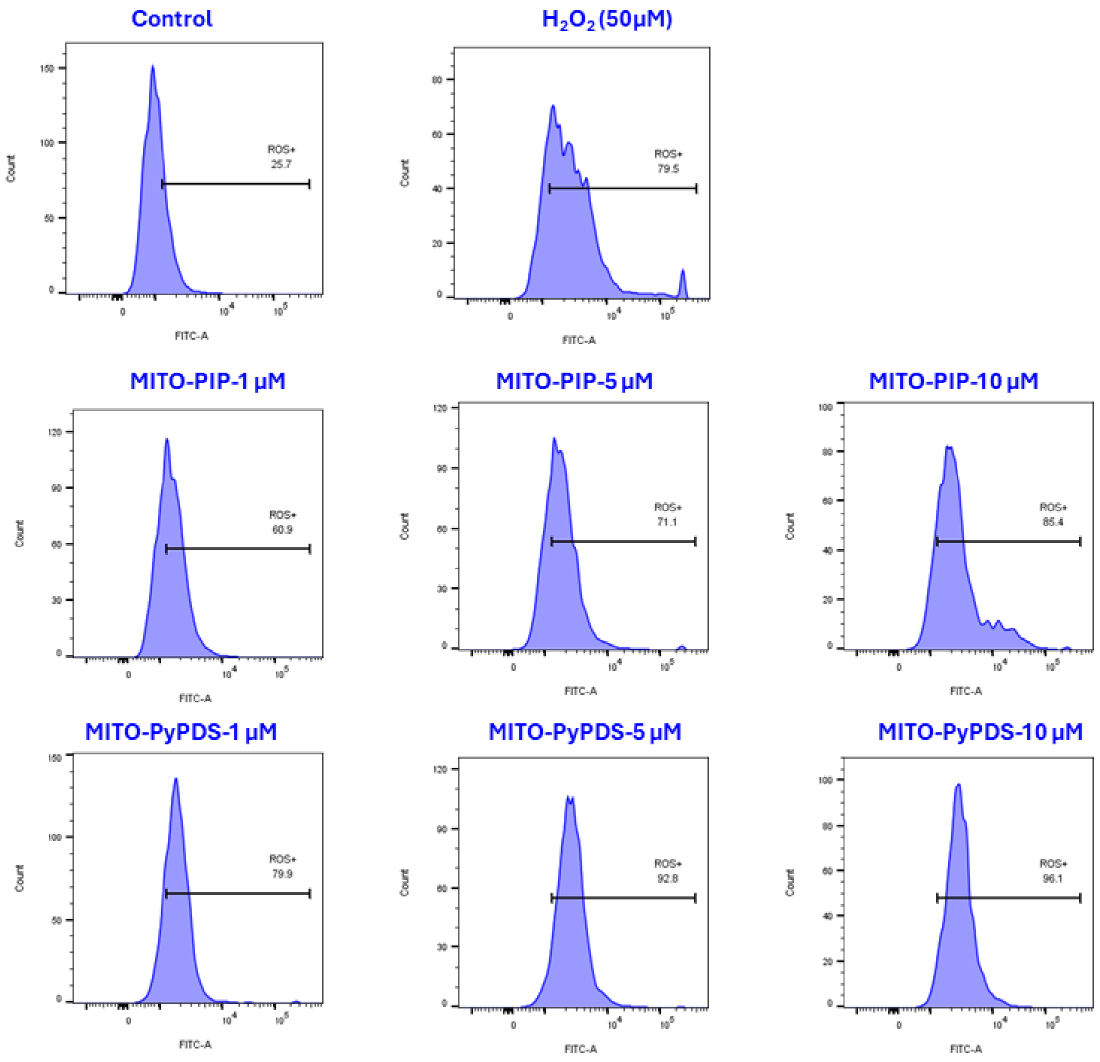

Supplementary Figure S45. Flow cytometry analysis of ROS generation. Indicated compounds treated at indicated concentrations for 24 hours except for H_2O_2 where treatment time is 1 hour followed by measurement of ROS.

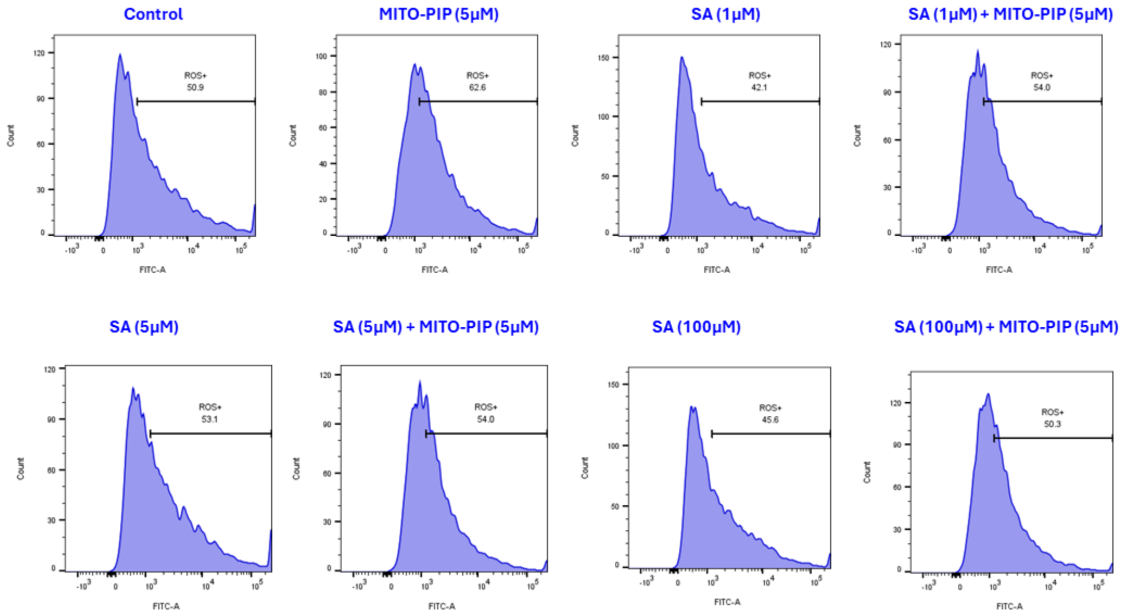

Supplementary Figure S46. Flow cytometry analysis of ROS generation. Indicated compounds treated at indicated concentrations for 24 hours followed by measurement of ROS.

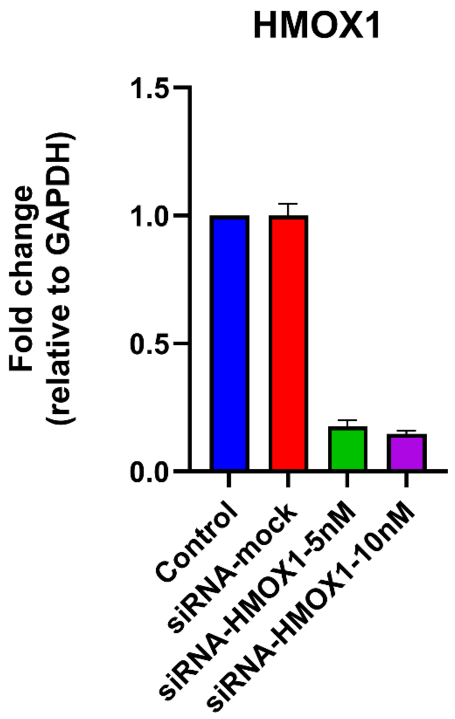

Supplementary Figure S47. HMOX1 levels in HEK-293 cells after being treated with siRNAs at the indicated concentration for 24 hours, followed by qPCR

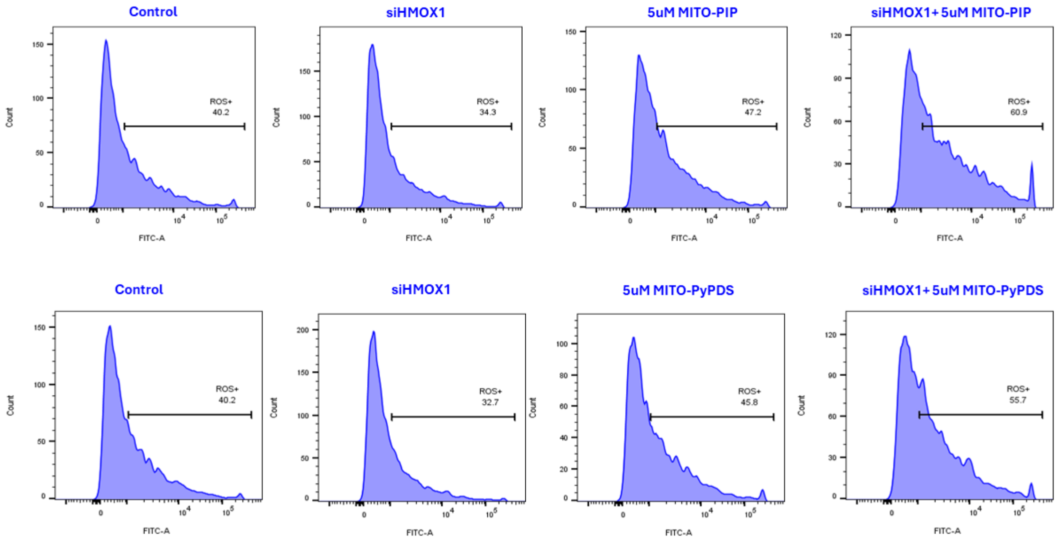

Supplementary Figure S48. Flow cytometry analysis of ROS generation. Indicated compounds together with siHMOX-1 treated at indicated concentrations for 24 hours followed by measurement of ROS.

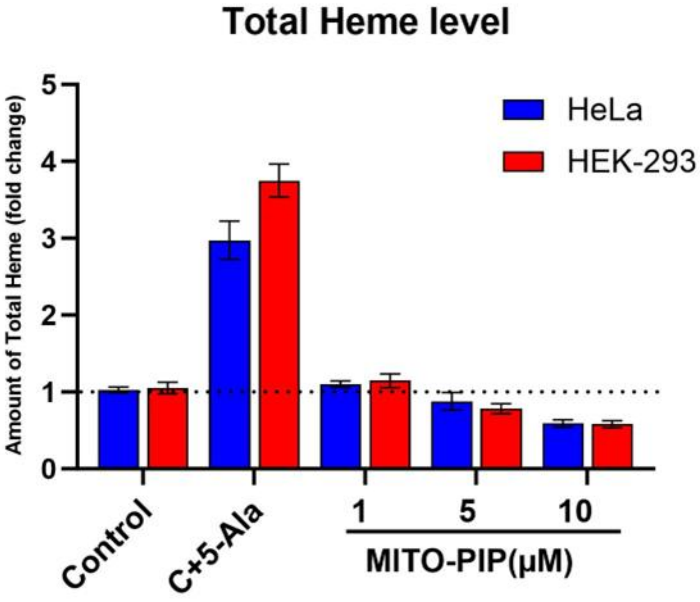

Supplementary Figure S49. Total heme levels in HeLa and HEK-293 cells treated with respective compounds for 24 hours followed quantification. The levels are in fold change compared to control cells without any treatment.

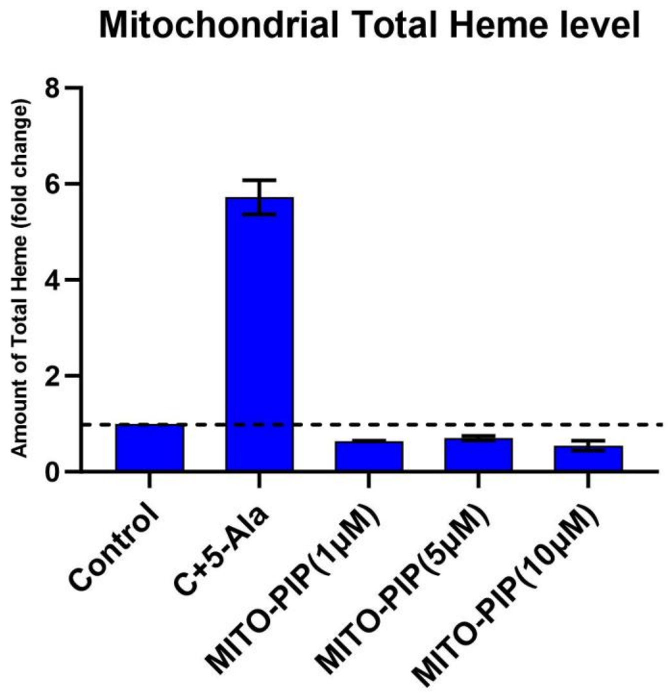

Supplementary Figure S50. Mitochondrial Total heme levels in HeLa cells treated with respective compounds for 24 hours followed by mitochondrial isolation and quantification. The levels are in fold change compared to control cells without any treatment.

Supplementary References

1. Mercer, T.R., Neph, S., Dinger, M.E., Crawford, J., Smith, M.A., Shearwood, A.-M.J., Haugen, E., Bracken, C.P., Rackham, O., Stamatoyannopoulos, J.A., et al. (2011). The human mitochondrial transcriptome. Cell *146*, 645–658.

2. Han, F., and Lillard, S.J. (2000). In-situ sampling and separation of RNA from individual mammalian cells. Anal. Chem. *72*, 4073–4079.

3. Hanna, D.A., Moore, C.M., Liu, L., Yuan, X., Dominic, I.M., Fleischhacker, A.S., Hamza, I., Ragsdale, S.W., and Reddi, A.R. (2022). Heme oxygenase-2 (HO-2) binds and buffers labile ferric heme in human embryonic kidney cells. J. Biol. Chem. *298*, 101549.

4. Ward, D.M., and Cloonan, S.M. (2019). Mitochondrial iron in human health and disease. Annu. Rev. Physiol. *81*, 453–482.

5. Holmes-Hampton, G.P., Jhurry, N.D., McCormick, S.P., and Lindahl, P.A. (2013). Iron content of Saccharomyces cerevisiae cells grown under iron-deficient and iron-overload conditions. Biochemistry *52*, 105–114.

6. Kaasik, A., Safiulina, D., Zharkovsky, A., and Veksler, V. (2007). Regulation of mitochondrial matrix volume. Am. J. Physiol. Cell Physiol. *292*, C157–C163.

7. Mestre-Fos, S., Ito, C., Moore, C.M., Reddi, A.R., and Williams, L.D. (2020). Human ribosomal G-quadruplexes regulate heme bioavailability. J. Biol. Chem. *295*, 14855–14865.

8. Westall, J. (1980). Chemical equilibrium including adsorption on charged surfaces. In Advances in Chemistry Advances in chemistry series. (AMERICAN CHEMICAL SOCIETY), pp. 33–44.
